# Feasibility of GPT-3 and GPT-4 for in-Depth Patient Education Prior to Interventional Radiological Procedures: A Comparative Analysis

**DOI:** 10.1007/s00270-023-03563-2

**Published:** 2023-10-23

**Authors:** Michael Scheschenja, Simon Viniol, Moritz B. Bastian, Joel Wessendorf, Alexander M. König, Andreas H. Mahnken

**Affiliations:** Department of Diagnostic and Interventional Radiology, University Hospital Marburg, Philipps-University of Marburg, Baldingerstrasse 1, 35043 Marburg, DE Germany

**Keywords:** Artificial intelligence, Patient education, Interventional radiology, Chat-GPT, Large language models

## Abstract

**Purpose:**

This study explores the utility of the large language models, GPT-3 and GPT-4, for in-depth patient education prior to interventional radiology procedures. Further, differences in answer accuracy between the models were assessed.

**Materials and methods:**

A total of 133 questions related to three specific interventional radiology procedures (Port implantation, PTA and TACE) covering general information as well as preparation details, risks and complications and post procedural aftercare were compiled. Responses of GPT-3 and GPT-4 were assessed for their accuracy by two board-certified radiologists using a 5-point Likert scale. The performance difference between GPT-3 and GPT-4 was analyzed.

**Results:**

Both GPT-3 and GPT-4 responded with (5) “completely correct” (4) “very good” answers for the majority of questions ((5) 30.8% + (4) 48.1% for GPT-3 and (5) 35.3% + (4) 47.4% for GPT-4). GPT-3 and GPT-4 provided (3) “acceptable” responses 15.8% and 15.0% of the time, respectively. GPT-3 provided (2) “mostly incorrect” responses in 5.3% of instances, while GPT-4 had a lower rate of such occurrences, at just 2.3%. No response was identified as potentially harmful. GPT-4 was found to give significantly more accurate responses than GPT-3 (*p* = 0.043).

**Conclusion:**

GPT-3 and GPT-4 emerge as relatively safe and accurate tools for patient education in interventional radiology. GPT-4 showed a slightly better performance. The feasibility and accuracy of these models suggest their promising role in revolutionizing patient care. Still, users need to be aware of possible limitations.

**Graphical Abstract:**

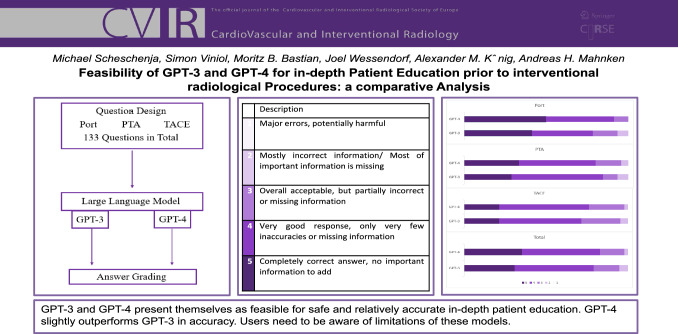

**Supplementary Information:**

The online version contains supplementary material available at 10.1007/s00270-023-03563-2.

## Introduction

As the field of artificial intelligence (AI) continues to evolve, there has been a growing interest in its implementation in healthcare and radiology [1–3]. Introduction of various open-source software enabled the general population to use AI. One such tool is Chat-GPT, a state-of-the-art large language model (LLM) developed by OpenAI (San Francisco, California, USA).

Chat-GPT and other LLMs utilize neural network algorithms trained on vast amounts of data to generate human-like text outputs, providing comprehensive information about various topics. LLMs can be used for health-related inquires by both professionals as well as patients. Still a common limitation of these LLMs is the risk of inaccurate information and so called “hallucinations,” which are outputs that are fabricated or not based on factual training data [4, 5]. Especially, in the area of healthcare, such inaccurate information may disrupt workflows or even be harmful.

### Patient Education in Interventional Radiology

Interventional radiology (IR) is a rapidly growing field that has revolutionized the way medical conditions are diagnosed and treated. Despite its advancements and rising popularity, many patients have limited knowledge and understanding of IR procedures [6, 7]. Patient education in IR is crucial to ensure that individuals are well informed and actively involved in their healthcare decisions [8]. It empowers patients to ask questions, understand potential risks and benefits, and make informed choices about their treatment. Furthermore, informed patients are more likely to comply with post-procedure instructions, which may lead to better overall outcomes [9].

While the use of internet to seek for health information is already a common phenomenon, LLMs may become a more significant source of information for patients. Given mentioned limitations, information provided by these kinds of software has to therefore be validated.

Ensuring sufficient accuracy and safety of information, LLMs like Chat-GPT can help bridging the gap between medical professionals and patients in IR.

This article explores the feasibility of using GPT-3 and GPT-4 for patient education prior to common IR procedures, in this case Port Implantation, percutaneous transluminal angioplasty (PTA) and transarterial chemoembolization (TACE), by asking in-depth questions about procedures and evaluating accuracy of given answers and differences between GPT-3 and GPT-4.

## Materials and Methods

### Study Design

A set of hypothetical patient questions pertaining to three specific IR procedures, namely Port Implantation, PTA, and TACE, was designed. Accuracy of answers to these questions provided by GPT-3 and GPT-4 as well as differences between both LLMs was evaluated.

### Question Design

A total of 133 questions pertaining to three common IR procedures, namely Port Implantation, PTA, and TACE were developed by two radiology residents and validated by a third radiologist with 7 years of experience in IR and patient education. Questions were designed corresponding to information conveyed during consent discussions and typical patient inquiries prior to these procedures. The questions covered various aspects of the procedure including general information, procedure preparation and the procedure itself, risks and complications as well as post-interventional aftercare. Selection of Port, PTA, and TACE as representative interventions was predicated on their status as most frequently executed procedures within our institution and them encompassing different interventional principles. Question portfolio consisted of 46 questions for Port Implantation, 45 questions for PTA, and 42 questions for TACE. Questions are provided as supplementary material together with their corresponding answers.

### Prompting

Prior to inputting the questions into the Chat-GPT-3/-4 system, the software was primed to respond to specific inquiries about the respective procedure. Priming example for PTA: *“Please answer the following questions about percutaneous transluminal angioplasty in peripheral arterial disease.”* All questions related to a particular procedure were asked in English language and in one sitting to maintain consistency. Answers provided by GPT-3 and GPT-4 were documented for further analysis.

### Response Grading

To assess the accuracy and quality of responses generated by GPT-3 and GPT-4, response grading was performed using a 5-point Likert scale (Table 1).

**Table 1:** 5-Point Likert-scale for evaluation of accuracy

	Description
1	Major errors, potentially harmful
2	Mostly incorrect information/Most of important information is missing
3	Overall acceptable, but partially incorrect or missing information
4	Very good response, only very few inaccuracies or missing information
5	Completely correct answer, no important information to add

Each response was independently checked for accuracy, discussed and evaluated by two board-certified radiologists with 4 and 7 years of experience in IR resulting in a unanimous grading. In case of disagreement, a third reader was consulted for a final grade decision. Readers were blinded to the respective LLM.

### Data Analysis

The grading scores assigned to each question were compiled and analyzed. Differences between GPT-3 and GPT-4 were analyzed using Wilcoxon signed-rank test. A *p* value of < 0.05 was considered significant. Statistical analysis was performed using Microsoft Excel (Microsoft, Redmond, Washington, USA) and SPSS (SPSS Version 29, IBM, Armonk, New York, USA).

## Results

A total of 133 Questions were inputted into Chat-GPT-3 and Chat-GPT-4 each.

**Fig. 1:**
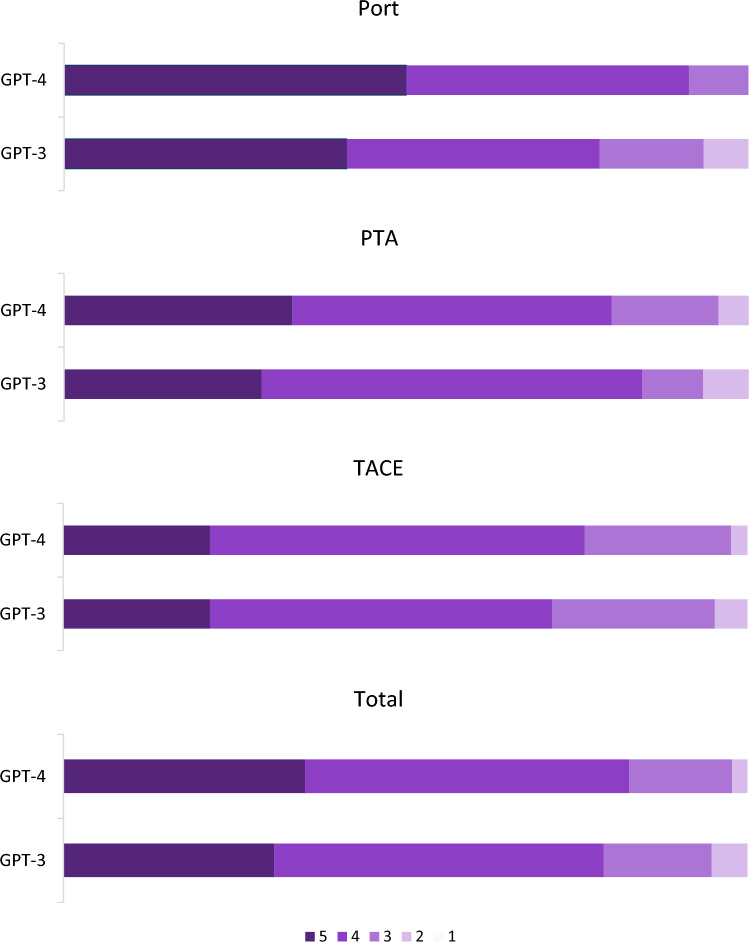
Bar chart to illustrate grading results for Port Implantation, percutaneous transarterial angioplasty (PTA) and transarterial chemoembolization based on a 5-point Likert-scale

Grading results are presented in Table 2 and Fig. 1. According to Wilcoxon signed-rank test, overall accuracy of answers was better in GPT-4 compared to GPT-3 (*p* = 0.043).

**Table 2:** Grading results for responses generated by GPT-3 and GPT-4 to questions related to port implantation, percutaneous transluminal angioplasty (PTA) and transarterial chemoembolization (TACE)

		GPT-3	GPT-4
Port (n = 46)	5	19 (41.3%)	23 (50.0%)
	4	17 (37.0%)	19 (41.3%)
	3	7 (15.2%)	4 (8.7%)
	2	3 (6.5%)	0 (0.0%)
	1	0 (0.0%)	0 (0.0%)
	Average:	4.13	4.4
PTA (n = 45)	5	13 (28.9%)	15 (33.3%)
	4	25 (55.6%)	21 (46.7%)
	3	4 (8.9%)	7 (15.6%)
	2	3 (6.7%)	2 (4.4%)
	1	0 (0.0%)	0 (0.0%)
	Average:	4.1	4.1
TACE (n = 42)	5	9 (21.4%)	9 (21.4%)
	4	21 (50%)	23 (54.8%)
	3	10 (23.8%)	9 (21.4%)
	2	2 (4.8%)	1 (2.4%)
	1	0 (0.0%)	0 (0.0%)
	Average:	3.9	4.0
Total (n = 133)	5	41 (30.8%)	47 (35.3%)
	4	64 (48.1%)	63 (47.4%)
	3	21 (15.8%)	20 (15.0%)
	2	7 (5.2%)	3 (2.3%)
	1	0 (0.0%)	0 (0.0%)
	Average:	4.0	4.2

## Discussion

The results of this study demonstrate the potential of AI-driven language models, notably GPT-3 and GPT-4, as resources for specific patient education in IR. GPT-3, which is already well refined and freely accessible to all, was further surpassed by GPT-4.

Interpreting the results, it is significant to note that both GPT-3 and GPT-4 were able to provide accurate answers to questions, covering general information about the procedures, preparation details, potential risks and complications, and post-interventional aftercare. The fact that a majority of responses were categorized as “completely correct” or “very good” is a testament to the utility of AI-driven language models in healthcare education. The marginally better performance of GPT-4 may reflect its more advanced model, indicating how refining these AI systems contribute to their improved effectiveness. Although there were rare instances where incorrect or incomplete information was provided; reassuringly, there were no responses that could potentially be dangerous. This can also be attributed to Chat-GPT's constant emphasis on discussing important medical questions with healthcare professionals.

In radiology, LLMs like Chat-GPT are already under investigation, showing their feasibilities and limits in clinical education, structured reporting or even automated determination of radiological study protocols [9–13]. A recently published study prompted general questions to Chat-GPT about patient education on IR achieving a satisfying accuracy of 88.5% [14]. This article, in turn, ventured to ask more specific questions, similar to those patients might have before undergoing such procedures.

### Limitations

The study did not incorporate real patients, making it unclear if the average patient could comprehend answers or phrase the right questions, considering that these models require priming input to deliver appropriate responses. Ambiguities may arise, especially when dealing with abbreviations. Still, our research remains pivotal, serving as a foundation in this domain. Future studies should evaluate applicability of these models with real patients. A crucial aspect not evaluated in this study is language comprehensibility of responses. However, it is noteworthy that Chat-GPT offers the flexibility to reformulate responses, making communication dynamic and adaptable. Assessing consistency of responses remains an area for future research. Further, while these models are trained on vast amounts of data, they lack semantic understanding. This deficiency might lead them to struggle in differentiating between best-practice and obsolete information. For future applications, this needs to be addressed.

## Conclusion

GPT-3 and GPT-4 present themselves as feasible for safe and relatively accurate in-depth patient education, still offering the potential for further improvement. GPT-4 slightly outperforms GPT-3 in accuracy. By addressing challenges, LLMs may be expected to obtain enormous applicability in healthcare.

### Supplementary Information

Below is the link to the electronic supplementary material.

Supplementary file1 (PDF 291 kb)

Supplementary file2 (PDF 340 kb)

Supplementary file3 (PDF 310 kb)

Supplementary file4 (PDF 280 kb)

Supplementary file5 (PDF 274 kb)

Supplementary file6 (PDF 246 kb)
