# Supplementary material for: Feasibility of GPT-3 and GPT-4 for in-Depth Patient Education Prior to Interventional Radiological Procedures: A Comparative Analysis
Source: Cardiovasc Intervent Radiol. 2023 Oct 23;47(2):245–50. doi: 10.1007/s00270-023-03563-2 (PMC10844465; doi:10.1007/s00270-023-03563-2)
Supplement: Supplementary file 1 — Supplementary file1 (PDF 291 kb) [file 270_2023_3563_MOESM1_ESM.pdf]

GPT-3 Port:

General Questions:

1. What is a port?

A port, in the context of medical procedures, refers to a device known as a port-a-cath (also called a port or port-catheter). It is a small, implantable device that is placed under the skin, usually in the chest or arm.

The port consists of two main components: a reservoir or chamber and a catheter. The reservoir is a small, round chamber made of a special material (such as titanium or plastic) that can be accessed with a needle. The catheter is a flexible tube that is connected to the reservoir and extends into a large vein near the heart.

The purpose of a port is to provide a secure and convenient access point for medications, fluids, or blood draws. It eliminates the need for repeated needle sticks, as healthcare professionals can access the port using a specialized needle that is inserted through the skin and into the reservoir. This makes it easier to administer chemotherapy, intravenous medications, or receive blood transfusions, especially for individuals who require frequent or long-term treatments.

Overall, a port is a valuable medical device that improves patient comfort and reduces the potential complications associated with repeated needle access.

2. Why do I need a port?

A port may be recommended for various medical reasons. Here are some common situations where a port may be needed:

Long-term intravenous (IV) therapy: If you require frequent or long-term administration of medications, such as chemotherapy, antibiotics, or nutrition, a port can provide a reliable and accessible route for delivering these treatments. It eliminates the need for finding a vein for each session, reducing discomfort and potential complications.

Chemotherapy treatment: Many cancer patients undergoing chemotherapy require multiple treatment sessions over an extended period. A port simplifies the administration of chemotherapy drugs and minimizes the risk of damaging veins from repeated needle sticks.

Blood transfusions: Individuals who need regular blood transfusions, such as those with certain blood disorders or undergoing certain treatments, can benefit from a port. It allows for easier access to veins and facilitates the transfusion process.

Frequent blood draws: Some medical conditions require frequent blood sampling for laboratory tests. A port can streamline this process by providing a convenient and accessible site for blood draws, reducing discomfort and preserving the integrity of peripheral veins.

Long-term medical conditions: For individuals with chronic diseases requiring ongoing intravenous treatments, such as cystic fibrosis or hemophilia, a port can simplify the administration of medications and improve quality of life.

The decision to use a port is typically made by your healthcare provider based on your specific medical needs and treatment plan. They will consider factors such as the duration and frequency of treatment, the potential impact on veins, and your overall health condition before recommending a port placement.

3. What alternatives do I have to a port?

If a port is not suitable for you or not recommended by your healthcare provider, there are alternative options available depending on your specific medical needs. Here are some alternatives to consider:

Peripheral IV line: For short-term IV therapy or occasional treatments, a peripheral IV line may be used. This involves inserting a small catheter into a vein in your hand, arm, or other peripheral location. However, repeated needle insertions may be required for each treatment session, which can be uncomfortable and potentially lead to vein damage over time.

Midline catheter: A midline catheter is a longer catheter that is inserted into a peripheral vein, typically in the upper arm. It is longer than a standard peripheral IV line and can remain in place for a few weeks to several months, depending on the situation. Midline catheters can be used for a variety of treatments, including medications and blood draws. However, they may not be suitable for all situations and may have limitations compared to ports, such as higher risk of complications or limited flow rates for certain treatments.

PICC line (Peripherally Inserted Central Catheter): A PICC line is a longer catheter that is inserted into a peripheral vein and threaded up to a larger vein near the heart. It can remain in place for weeks to months and provides a central access point for medications and blood draws. PICC lines are commonly used for long-term IV therapy or when frequent blood sampling is required. However, they are more prone to complications such as infection or vein thrombosis compared to ports.

External catheter devices: In some cases, external catheter devices or wearable devices can be used for specific treatments. These devices are typically attached externally to the skin and allow for

medication administration or blood sampling without the need for an implanted device. They may be used for certain specialized treatments or situations where other options are not feasible.

It's important to consult with your healthcare provider to discuss the alternatives that are appropriate for your specific medical condition and treatment requirements. They will consider factors such as the duration of treatment, the type of medications or therapies involved, and your individual health status to determine the most suitable alternative for you.

4. What are the advantages of having a port?

Having a port offers several advantages compared to other methods of intravenous access. Here are some key advantages of having a port:

Convenience and ease of access: Ports provide a dedicated and easily accessible site for medication administration, blood draws, or transfusions. Healthcare professionals can quickly access the port using a specialized needle, saving time and reducing discomfort compared to searching for a suitable vein for each treatment session.

Reduced needle sticks: With a port, you can avoid the need for repeated needle sticks. This is particularly beneficial for individuals requiring frequent or long-term treatments, such as chemotherapy. Minimizing the number of needle insertions can enhance patient comfort and reduce the risk of complications associated with multiple needle sticks, such as vein damage or infection.

Preserved peripheral veins: By using a port, the peripheral veins in your arms and hands are spared from the stress of repeated needle punctures. Preserving these veins can be crucial for future medical procedures or emergency situations that may require peripheral venous access.

Improved quality of life: Ports can significantly enhance the quality of life for individuals undergoing long-term treatments. The reduced pain and inconvenience associated with finding and accessing veins can alleviate stress and anxiety, allowing you to focus on your overall well-being.

Optimal medication delivery: Ports provide a direct route to a large central vein near the heart, enabling medications to be administered at higher flow rates. This is particularly important for chemotherapy or other treatments that require the infusion of larger volumes of medications or fluids.

Long-term use: Ports are designed for long-term use, often remaining in place for months or even years. They are durable and reliable, and with proper care, they can withstand repeated access without the need for frequent replacements.

Improved cosmetic appearance: Ports are usually placed under the skin, resulting in a discreet and less visible appearance compared to external catheters or other forms of intravenous access. This can be beneficial for individuals concerned about the aesthetic impact of medical devices.

It's important to note that while ports offer many advantages, they also carry some risks and require regular care and maintenance. Your healthcare provider will provide instructions on how to properly care for your port and monitor for any potential complications.

5. What medication can be administered through a port?

Ports can be used for the administration of a wide range of medications, fluids, and treatments. Some common medications that can be administered through a port include:

Chemotherapy drugs: Ports are frequently used for the administration of chemotherapy medications, which are potent drugs used to treat various types of cancer. The port allows for the infusion of these drugs directly into the bloodstream.

Antibiotics: Ports can be utilized for long-term antibiotic therapy, especially in cases where frequent or prolonged administration is required. This is often seen in individuals with chronic infections or conditions such as cystic fibrosis.

Parenteral nutrition: When a person is unable to consume food orally or through the gastrointestinal tract, a port can be used for the delivery of specialized nutrition solutions directly into the bloodstream. This is known as parenteral nutrition and is typically used in cases of severe malnutrition or gastrointestinal disorders.

Pain management medications: Ports can be employed for the delivery of pain medications, such as opioids, in individuals with chronic pain conditions. This allows for continuous or controlled administration of pain relief medications.

Immunoglobulins: Ports can be used for the infusion of immunoglobulin therapies, which are often administered to individuals with immune deficiencies or autoimmune disorders.

Blood transfusions: Ports can facilitate the administration of blood transfusions in individuals who require regular transfusions due to conditions like anemia, bleeding disorders, or certain cancers.

It's important to note that the specific medications and treatments administered through a port will depend on your individual medical condition and treatment plan. Your healthcare provider will

determine the appropriate medications and fluids to be administered through the port based on your specific needs.

6. How is the medication administered?

Medications are administered through a port using a specific technique. Here's a general overview of how medications are typically administered through a port:

Preparation: The healthcare professional will gather the necessary medications, sterile supplies, and equipment. They will perform hand hygiene and put on sterile gloves to maintain aseptic technique.

Accessing the port: The skin over the port site will be cleaned with an antiseptic solution to prevent infection. The healthcare professional will then use a specialized needle called a Huber needle to access the port. The needle is inserted through the skin and into the port's septum, which is a self-sealing rubber or silicone membrane covering the reservoir.

Flushing: Before medication administration, the port is flushed with a sterile saline solution to ensure it is patent and free from any blockages. This also helps maintain the integrity of the port and prevent blood clot formation.

Medication administration: The prescribed medication is then prepared and attached to the Huber needle or an extension set connected to the port. The medication is slowly infused into the port, allowing it to flow through the catheter and into the bloodstream.

Flushing after medication administration: Once the medication is fully administered, the port is flushed again with a sterile saline solution to ensure complete delivery of the medication and prevent any residual medication from remaining in the catheter or port.

Needle removal and dressing: After the medication administration and flushing, the Huber needle is carefully removed from the port. A sterile dressing or transparent dressing may be applied over the port site to protect it and maintain cleanliness.

It's important to note that the specific administration technique and protocols may vary depending on the healthcare facility, the type of medication being administered, and the individual patient's needs. Your healthcare provider and the nursing staff will provide you with detailed instructions on the specific procedures for medication administration through your port.

7. What does a Port look like?

A port consists of two main components: the reservoir (also known as the chamber or port body) and the catheter. Here's a description of what a typical port looks like:

Reservoir (Port Body): The reservoir is a small, round, and usually flat device made of a biocompatible material such as titanium or plastic. It is typically placed underneath the skin, often in the chest or upper arm area. The port body has a self-sealing septum on the top, which is made of a rubber or silicone material. The septum is where the needle is inserted to access the port for medication administration or blood draws.

Catheter: The catheter is a flexible tube that connects to the reservoir. It extends from the port body and is threaded into a large vein near the heart, usually the superior vena cava or subclavian vein. The catheter is responsible for delivering medications or fluids directly into the bloodstream.

The port body is usually covered with a sterile dressing or a specialized port access kit when not in use to maintain cleanliness and prevent infection. The dressing or kit includes a protective barrier over the port site, which allows for easy access when needed.

It's important to note that there can be variations in the design and appearance of ports, including different shapes, sizes, and access methods. The specific type of port used may depend on factors such as the manufacturer, the individual's anatomy, and the purpose of the port (e.g., chemotherapy port, long-term IV therapy port). Your healthcare provider or a trained healthcare professional will provide you with more detailed information and show you the specific port you will be using.

8. Can blood be drawn through the port as well?

Yes, blood can be drawn through a port. One of the advantages of having a port is that it can serve as a convenient access point for blood draws in addition to medication administration. Here's an overview of how blood draws are typically performed using a port:

Preparation: The healthcare professional will gather the necessary sterile equipment, including a syringe or vacuum tube for blood collection, and perform hand hygiene to maintain aseptic technique.

Accessing the port: The skin over the port site will be cleaned with an antiseptic solution. The healthcare professional will then use a specialized needle called a Huber needle to access the port. The Huber needle is inserted through the skin and into the septum of the port to create a connection.

Blood collection: Once the needle is in place and connected to the port, the healthcare professional will slowly withdraw the required amount of blood using a syringe or attach a vacuum tube to the needle to collect the blood directly into the tube. The blood flows through the catheter of the port and into the syringe or tube for laboratory analysis.

Flushing: After the blood draw, the port is flushed with a sterile saline solution to ensure complete blood collection, prevent clotting, and maintain the patency of the port.

Needle removal and dressing: Once the blood draw is complete, the Huber needle is carefully removed from the port. A sterile dressing or transparent dressing may be applied over the port site to protect it and maintain cleanliness.

It's important to note that blood draws through a port should be performed by trained healthcare professionals who are familiar with port access and blood collection procedures. They will follow proper protocols and guidelines to ensure the accuracy of the blood sample and minimize the risk of infection or complications.

9. Where will a port be implanted?

The exact location of port implantation can vary depending on factors such as the patient's medical condition, treatment plan, and individual anatomy. The most common locations for port implantation are:

Chest: The upper chest, just below the collarbone, is a frequently used location for port placement. This area provides easy access for healthcare professionals and allows for comfortable patient positioning during port access and medication administration.

Upper Arm: Some ports may be implanted in the upper arm, particularly if the chest area is not suitable due to prior surgeries, radiation therapy, or other anatomical considerations. Arm ports can be placed on the inner or outer side of the upper arm.

The specific placement of the port within the chosen location can also vary. The healthcare provider will determine the optimal placement based on factors such as the patient's anatomy, the expected duration of port use, and the accessibility required for proper port access and care.

It's important to note that the decision on port placement will be made by your healthcare provider based on your specific circumstances and treatment needs. They will consider factors such as the type of treatment, patient comfort, and potential impact on daily activities when determining the most suitable location for port implantation.

10. How long does a port remain in place?

The duration for which a port remains in place can vary depending on several factors, including the patient's medical condition, treatment plan, and individual circumstances. Ports are typically designed for long-term use and can remain in place for months to years. Here are some general guidelines:

Temporary use: In some cases, ports may be implanted for temporary use, such as for a specific course of treatment or a defined period of intravenous therapy. After the treatment or therapy is completed, the port may be removed.

Long-term use: Ports can be used for long-term or chronic conditions that require ongoing intravenous access. In such cases, ports can remain in place for extended periods, often ranging from several months to several years. The specific duration depends on the patient's needs and the recommendations of the healthcare provider.

Maintenance and monitoring: Ports require regular care and maintenance to ensure proper functioning and minimize the risk of complications. This includes regular flushing of the port with sterile saline or heparin to prevent blockages and routine dressing changes. Your healthcare provider will provide specific guidelines on port care and monitoring to ensure its optimal performance.

It's important to note that the decision on how long a port remains in place will be determined by your healthcare provider based on your individual medical needs and treatment plan. They will assess factors such as the type of treatment, expected duration of therapy, and the condition of the port and catheter during regular follow-up visits. If there are any concerns or complications with the port, your healthcare provider may decide to remove or replace it as necessary.

Preparation and Procedure:

1. How should I prepare for the surgery?

Preparing for port placement surgery involves several steps to ensure a smooth and successful procedure. Here are some general guidelines to help you prepare:

Consultation and Preoperative Evaluation: Your healthcare provider will schedule a consultation to discuss the need for a port and explain the procedure in detail. They may also perform a preoperative evaluation, which may include blood tests, imaging studies, and a physical examination.

Medical History and Medications: Provide your healthcare provider with a comprehensive medical history, including any chronic conditions, previous surgeries, allergies, and medications you are currently taking. They will guide you on any necessary adjustments to your medications before the surgery, such as temporarily discontinuing blood-thinning medications.

Fasting: You will likely be instructed to refrain from eating or drinking for a specific period before the surgery, typically starting from midnight the night before the procedure. Follow the fasting instructions provided by your healthcare team to ensure your stomach is empty during the surgery.

Anesthesia and Sedation: If the port placement procedure requires anesthesia or sedation, your healthcare provider will provide specific instructions regarding fasting and medication guidelines. They will explain the risks, benefits, and any potential complications associated with anesthesia.

Transportation and Support: Arrange for transportation to and from the hospital or surgical center on the day of the procedure, as you may not be able to drive yourself. It's also helpful to have a family member or friend accompany you for support during the process.

Clothing and Personal Items: Wear comfortable, loose-fitting clothing on the day of the procedure. Avoid wearing any jewelry, makeup, or contact lenses. Follow the instructions provided by the healthcare facility regarding personal items to bring or leave at home.

Follow Preoperative Instructions: Your healthcare provider will provide specific preoperative instructions, such as bathing or showering with a special antiseptic soap the night before or morning of the surgery. Follow these instructions carefully to minimize the risk of infection.

Clarify Concerns and Questions: If you have any concerns or questions about the surgery, anesthesia, or recovery process, don't hesitate to discuss them with your healthcare provider. They are there to address your concerns and provide the necessary information.

Remember that these are general guidelines, and your healthcare provider may provide additional or specific instructions based on your individual case. It's essential to closely follow the instructions provided to ensure a safe and successful surgery.

2. Do I need someone to accompany me on the day of the surgery?

It is generally recommended to have someone accompany you on the day of the port placement surgery. Here's why having a companion can be beneficial:

Support and Comfort: Having a family member or friend with you can provide emotional support and comfort before and after the surgery. They can help alleviate any anxiety or stress you may be experiencing and provide reassurance throughout the process.

Transportation: Following the surgery, you may experience some effects from the anesthesia or sedation, such as drowsiness or grogginess. Having someone available to drive you home is important since you may not be able to operate a vehicle immediately after the procedure.

Communication and Information: Your companion can help communicate with the healthcare team, ask questions on your behalf, and ensure that you have a clear understanding of any post-operative instructions or precautions. They can take notes during discussions with the healthcare provider and provide you with valuable information when needed.

Assistance with Personal Needs: Depending on the level of post-operative discomfort or limitation, you may require assistance with daily activities, such as preparing meals, getting dressed, or managing household chores. Having someone by your side can be helpful during the initial recovery period.

Advocacy: Your companion can serve as an advocate for your needs and ensure that you receive proper care and attention during your hospital stay or outpatient procedure. They can help address any concerns or clarify information on your behalf.

While having someone accompany you is generally recommended, it ultimately depends on your specific circumstances and the policies of the healthcare facility. It's best to check with your healthcare provider or the surgical center to confirm if having a companion is required or strongly encouraged for your port placement surgery.

3. Am I allowed to eat and drink before the surgery?

In most cases, you will be instructed to refrain from eating or drinking for a specific period of time before the port placement surgery. This is done to reduce the risk of complications related to anesthesia, such as aspiration (inhaling stomach contents into the lungs) during the procedure. Here are some general guidelines:

Fasting from Solid Foods: Typically, you will be instructed to avoid consuming solid foods for a certain period before the surgery. This fasting period is typically around 6 to 8 hours, although it may vary depending on the specific instructions provided by your healthcare team. It's important to adhere to this fasting period to ensure your stomach is empty during the surgery.

Clear Liquids: In some cases, you may be allowed to drink clear liquids up to a few hours before the surgery. Clear liquids include water, clear broth, apple juice (without pulp), and black coffee or tea without milk or creamer. However, it's essential to follow the specific instructions provided by your healthcare provider, as they may have specific guidelines regarding clear liquids before surgery.

Medication Guidelines: Your healthcare provider will provide specific instructions regarding the use of medications before the surgery. This may include instructions to take certain medications with a small sip of water or to temporarily discontinue certain medications, especially those that can affect blood clotting or interact with anesthesia.

It's crucial to strictly follow the fasting instructions provided by your healthcare provider to ensure your safety during the procedure. Failure to adhere to the fasting guidelines may result in the surgery being postponed or canceled to prevent potential complications.

It's important to note that these are general guidelines, and specific fasting instructions may vary based on your individual circumstances, the type of anesthesia used, and the policies of the healthcare facility. Always consult your healthcare provider or the surgical center where the procedure will be performed to receive the most accurate and up-to-date instructions regarding fasting before your port placement surgery.

4. Can I take my medications before the surgery?

The administration of medications before port placement surgery can vary depending on the specific medications you take and the instructions provided by your healthcare provider. Here are some general guidelines:

Follow Healthcare Provider's Instructions: It's crucial to follow the instructions given by your healthcare provider regarding the use of medications before the surgery. They will provide specific guidelines tailored to your individual needs and medical condition.

Inform Your Healthcare Provider: Make sure to inform your healthcare provider about all the medications you are currently taking, including prescription medications, over-the-counter medications, vitamins, and supplements. This includes any medications that may not seem directly related to your surgery. Providing this information allows your healthcare provider to make informed decisions about medication management before and after the procedure.

Blood-Thinning Medications: If you are taking blood-thinning medications, such as anticoagulants (e.g., warfarin, heparin) or antiplatelet drugs (e.g., aspirin, clopidogrel), your healthcare provider may provide specific instructions about managing these medications before the surgery. In some cases, you may be advised to temporarily discontinue or adjust the dosage of these medications to reduce the risk of excessive bleeding during and after the procedure. It is essential to follow your healthcare provider's guidance regarding these medications.

Medications with Sips of Water: Your healthcare provider may allow you to take specific medications with a small sip of water on the day of the surgery. This is typically done to ensure that certain essential medications are not abruptly stopped, especially if they are critical for your health and well-being. However, it's important to consult your healthcare provider to confirm which medications can be taken with water and any specific time frames or dosing instructions.

Always consult your healthcare provider or the surgical center where the procedure will be performed to receive clear instructions regarding medication management before your port placement surgery. They will provide you with the most accurate and up-to-date information based on your specific medical condition and the planned surgery. It is important to adhere to these instructions to ensure your safety and optimize the success of the procedure.

5. Which medications am I not allowed to take before the surgery?

The specific medications that you may be instructed to avoid before port placement surgery can vary depending on various factors, including your medical condition, the type of surgery, and the

preferences of your healthcare provider. However, there are certain classes of medications that are commonly associated with increased bleeding risk or interactions with anesthesia. Here are some examples:

Blood-Thinning Medications: Anticoagulants (e.g., warfarin, heparin, enoxaparin) and antiplatelet drugs (e.g., aspirin, clopidogrel, ticagrelor) are medications that can interfere with normal blood clotting. Depending on the specific circumstances, your healthcare provider may instruct you to temporarily discontinue these medications before the surgery. This is done to minimize the risk of excessive bleeding during and after the procedure. However, the decision to stop or adjust these medications should be made in consultation with your healthcare provider, as the potential benefits and risks need to be carefully evaluated.

Nonsteroidal Anti-inflammatory Drugs (NSAIDs): NSAIDs such as ibuprofen, naproxen, and aspirin (at higher doses) can interfere with platelet function and increase the risk of bleeding. Your healthcare provider may advise avoiding NSAIDs for a certain period before the surgery. However, if you are taking NSAIDs for a specific medical condition, it's important to discuss this with your healthcare provider, as the decision to discontinue or adjust these medications should be made on an individual basis.

Herbal Supplements and Alternative Medicines: Some herbal supplements and alternative medicines can have blood-thinning effects or interact with anesthesia. Examples include garlic, ginger, ginkgo biloba, and certain herbal teas. It's important to inform your healthcare provider about any herbal supplements or alternative medicines you are taking so they can provide guidance on which ones to avoid before the surgery.

It's essential to communicate openly with your healthcare provider about all the medications you are taking, including prescription medications, over-the-counter medications, vitamins, and supplements. Your healthcare provider will provide specific instructions regarding which medications to avoid or continue before the surgery based on your individual circumstances. Follow their guidance closely to ensure your safety and optimize the success of the procedure.

6. How does the surgery proceed?

The port placement surgery typically follows a general sequence of steps, although the specific details may vary depending on the surgical technique used and the preferences of the surgeon. Here is a general overview of how the port placement surgery proceeds:

Anesthesia: Before the surgery begins, you will be given anesthesia or sedation to ensure your comfort during the procedure. The type of anesthesia used can vary, and your healthcare provider will discuss the options with you beforehand.

Incision and Pocket Creation: The surgeon will make a small incision in the chosen area for port placement, typically in the upper chest or upper arm. The incision is made in a way that minimizes

visibility and provides easy access to the port. A pocket is then created beneath the skin, where the port will be implanted.

Port Placement: The surgeon will carefully position the port within the pocket, securing it in place using sutures or other fixation techniques. The port consists of a small chamber made of durable materials (such as titanium or plastic) and a catheter, which is a thin flexible tube that is inserted into a large vein.

Catheter Insertion: The catheter is inserted into a vein, often the superior vena cava (a large vein near the heart), using imaging guidance such as fluoroscopy or ultrasound. The catheter is threaded through the vein until the tip reaches the appropriate location, ensuring proper positioning for the administration of medications.

Incision Closure: The incision is carefully closed using sutures or surgical adhesive. The surgeon will take care to ensure proper wound closure and minimize scarring.

Dressing and Bandaging: After the incision is closed, a sterile dressing or bandage is applied to protect the surgical site and minimize the risk of infection. The dressing may include a transparent cover that allows for visual inspection of the port site.

Postoperative Care and Recovery: Once the surgery is complete, you will be monitored in a recovery area to ensure your stability and comfort. Your vital signs will be monitored, and any postoperative pain or discomfort will be addressed. The healthcare team will provide specific instructions on postoperative care, including port care, pain management, activity restrictions, and follow-up appointments.

It's important to note that the above steps are a general overview, and the actual procedure may involve additional or slightly different steps depending on the specific circumstances and surgical technique employed. The surgical team will guide you through the process and ensure your safety and well-being throughout the surgery.

7. Will I be awake during the surgery?

The level of consciousness during port placement surgery can vary depending on the specific circumstances and the type of anesthesia or sedation used. Generally, there are three main options:

General Anesthesia: In many cases, port placement surgery is performed under general anesthesia. With general anesthesia, you will be completely unconscious and unaware during the surgery. The anesthesia will be administered by an anesthesiologist, and you will be closely monitored throughout the procedure.

Local Anesthesia with Sedation: Alternatively, the surgeon may choose to use local anesthesia with sedation. In this case, local anesthesia is used to numb the surgical area, while sedatives are given to induce a state of relaxation and drowsiness. With this combination, you may be semi-conscious or in a "twilight" state during the surgery. You may not feel pain or remember much of the procedure, but you will be able to respond to commands or light stimulation.

Local Anesthesia Only: In some cases, depending on the complexity of the surgery and the patient's overall health, the procedure may be performed under local anesthesia alone. With local anesthesia, the surgical area is numbed, but you remain fully conscious throughout the procedure. You may feel some pressure or mild discomfort, but it should not be painful. The surgical team will ensure your comfort and may provide additional relaxation measures if needed.

The choice of anesthesia or sedation method is based on several factors, including the complexity of the surgery, your medical condition, and your preferences. Your healthcare provider and the surgical team will discuss the options with you beforehand and determine the most suitable approach for your specific case.

It's important to communicate openly with your healthcare provider and ask any questions or express any concerns you may have about anesthesia or sedation before the surgery. They will provide you with detailed information and ensure that you are comfortable and well-informed about the process.

8. Where will the port be implanted?

The port is typically implanted in a specific location based on the individual patient's needs and the recommendations of the healthcare provider. The most common placement sites for a port are:

Upper Chest: The upper chest, usually on the side below the collarbone (clavicle), is a common location for port placement. This area allows for easy access and is convenient for both patients and healthcare providers.

Upper Arm: In some cases, the port may be implanted in the upper arm, specifically in the area between the shoulder and elbow. This location may be chosen for various reasons, such as patient preference, accessibility, or specific medical considerations.

The choice of the placement site depends on factors such as the patient's body anatomy, medical condition, accessibility, and the healthcare provider's preference. The healthcare provider will assess these factors and determine the optimal placement site for the port based on individual needs.

It's important to note that the port is implanted beneath the skin, and the catheter attached to the port is threaded through a vein to reach a larger central vein, often the superior vena cava near the heart. The port itself is not visible on the surface of the skin but can be accessed by healthcare providers using a specialized needle through a small puncture site.

Your healthcare provider will discuss the planned location for port placement with you prior to the surgery, explaining the rationale and answering any questions you may have. They will ensure that the chosen placement site is appropriate for your specific medical condition and treatment requirements.

9. Will the port be implanted on the left or right side?

The decision to implant the port on the left or right side of the body depends on various factors, including the patient's anatomy, medical condition, and the preferences of the healthcare provider. Here are some general considerations:

Dominant Hand: If you are right-handed, the port may be implanted on the left side of the body, and vice versa. This is because the non-dominant side may be preferred to minimize interference with daily activities and to ensure easier access to the port for self-care or medication administration.

Accessibility and Convenience: The placement site for the port should be easily accessible for both the patient and the healthcare provider. The upper chest and upper arm are common locations chosen for their accessibility and convenience. The healthcare provider will assess the patient's body anatomy and determine the most suitable side based on factors such as the location of veins, muscle bulk, and any existing medical conditions.

Previous Surgeries or Medical Procedures: If a patient has previously undergone surgeries or medical procedures on a particular side of the body, the opposite side may be chosen for port placement to minimize any potential complications or interference with previous surgical sites.

Individual Factors: Each patient's case is unique, and individual factors may influence the decision. This can include factors such as personal preferences, medical conditions, or the presence of any anatomical variations that could affect the placement of the port.

Ultimately, the decision regarding the placement of the port (left or right side) is made by the healthcare provider based on a comprehensive assessment of the patient's needs and considerations. Your healthcare provider will discuss the planned placement site with you prior to the surgery and provide a clear explanation of the rationale behind the chosen side. If you have any specific concerns or preferences, it is important to discuss them with your healthcare provider, as they will take your input into consideration when making the final decision.

10. How long does the surgery take?

    The duration of port placement surgery can vary depending on various factors, including the specific technique used, the complexity of the procedure, and individual patient considerations. On average, the surgery may take approximately 1 to 2 hours from the time you enter the operating room to the completion of the procedure. However, it's important to note that this is just an estimate, and the actual duration may be shorter or longer depending on the circumstances.

The surgical team will work diligently to complete the procedure efficiently while ensuring that all necessary steps are performed accurately and safely. The surgeon will inform you about the expected duration of the surgery before the procedure, as it can help you and your accompanying person plan accordingly.

It's worth mentioning that the time spent in the operating room is not the only aspect to consider. Before the surgery, there will be additional time for preoperative preparations, including anesthesia administration, patient positioning, and ensuring all necessary equipment and supplies are ready. After the surgery, you will be transferred to a recovery area where you will be monitored until you are stable and awake.

Remember, these timeframes are approximate, and actual surgery times can vary. It's important to follow the guidance of your healthcare provider and surgical team regarding the specific timeline for your port placement surgery. They will be able to provide you with more accurate information based on your individual case.

11. Is the surgery painful?

During the port placement surgery, you should not experience any pain as you will be under the effect of anesthesia or sedation. The anesthesia will ensure that you are comfortable and do not feel any pain during the procedure. The surgical team will monitor your vital signs and adjust the anesthesia as needed to maintain your comfort throughout the surgery.

After the surgery, it is common to experience some discomfort or mild pain at the surgical site. This can vary from person to person, but it is generally manageable with appropriate pain management strategies. Your healthcare provider will prescribe or recommend pain medications to help alleviate any discomfort following the surgery.

It's important to communicate with your healthcare provider and the surgical team about any pain or discomfort you may experience after the surgery. They can provide you with appropriate pain management options and support to ensure your comfort during the recovery period.

Remember, the primary goal of the surgical team is to minimize your pain and discomfort throughout the entire process. They will take all necessary measures to ensure your well-being and provide you with the necessary support and pain management strategies.

12. Will I typically be admitted as an inpatient for the surgery?

No, typically port placement surgery is not performed as an inpatient procedure. It is commonly done on an outpatient basis, meaning you will not be admitted as an inpatient and can typically go home the same day after the surgery.

Outpatient port placement has become a standard practice for many patients. It offers several advantages, including convenience, reduced healthcare costs, and decreased risk of hospital-acquired infections. Following the surgery, you will be monitored in a recovery area for a period of time to ensure your stability and comfort. Once you have recovered sufficiently and there are no complications, you will be allowed to go home.

However, there may be certain circumstances where your healthcare provider determines that an overnight hospital stay is necessary. This could be due to specific medical conditions, anticipated postoperative complications, or other factors unique to your case. In such cases, your healthcare provider will inform you in advance and make the necessary arrangements for your admission as an inpatient.

It's important to discuss the specific plans for your surgery with your healthcare provider. They will provide you with detailed information about the expected duration of your hospital stay or whether the surgery is planned as an outpatient procedure. They will also give you any specific instructions regarding preoperative and postoperative care to ensure a smooth and successful recovery.

Risks and Complications:

1. How safe is the procedure?

Port placement surgery is generally considered safe, but as with any surgical procedure, there are potential risks and complications. It is important to discuss these risks with your healthcare provider and weigh them against the potential benefits of having a port.

Some potential risks and complications associated with port placement surgery may include:

Infection: There is a risk of infection at the surgical site or along the path of the catheter. To minimize this risk, the surgical team follows strict sterile techniques, and you will be given antibiotics before and/or after the surgery.

Bleeding or Hematoma: There is a small risk of bleeding during or after the procedure, which can lead to the formation of a hematoma (a collection of blood). Proper surgical technique and careful monitoring help minimize this risk.

Catheter-related Complications: The catheter may develop complications such as blockage, kinking, or dislodgement. Regular flushing and proper care can help prevent these complications, but they can still occur and may require intervention.

Catheter-related Blood Clots: In rare cases, a blood clot may form around the catheter or in the vein where it is placed. This can potentially lead to more serious complications, such as deep vein thrombosis (DVT) or pulmonary embolism (PE). Precautions are taken to minimize this risk, such as using anticoagulant medications or periodically flushing the catheter.

Pneumothorax: In some cases, there is a small risk of a pneumothorax (collapsed lung) if the port is placed in the upper chest area. This risk is minimized by using imaging techniques, such as fluoroscopy or ultrasound, during the catheter insertion.

Allergic Reactions or Sensitivities: There is a small possibility of developing an allergic reaction to the materials used in the port or the medications administered through it. If you have known allergies or sensitivities, it is important to inform your healthcare provider beforehand.

These risks are generally low, and the benefits of having a port often outweigh the potential complications. Your healthcare provider will thoroughly evaluate your medical history and specific circumstances to assess the risks and benefits for your individual case.

It's important to discuss any concerns or questions you may have about the safety of the procedure with your healthcare provider. They will provide you with detailed information, address your concerns, and guide you through the decision-making process.

2. What complications can occur during the procedure?

During port placement surgery, complications can occur, although they are relatively rare. Some potential complications that can arise during the procedure include:

Bleeding: There is a risk of bleeding from the surgical site or blood vessels during the procedure. The surgical team takes precautions to minimize bleeding, but it can still occur. In most cases, any bleeding is controlled during the surgery, and additional measures may be taken if necessary.

Injury to Surrounding Structures: There is a small risk of accidental injury to nearby structures, such as blood vessels, nerves, or organs, during the insertion of the port or catheter. Surgeons take great care to minimize this risk, and the use of imaging techniques, such as fluoroscopy or ultrasound, may be employed to guide the procedure and ensure accurate placement.

Pneumothorax: If the port is placed in the upper chest area, there is a small risk of pneumothorax, which is a condition where air enters the space between the lung and the chest wall, causing the lung to partially or completely collapse. The risk is minimized by using imaging techniques to guide the placement of the catheter and avoiding lung tissue during the procedure.

Infection: Although infection is more commonly a postoperative complication, there is a slight risk of infection at the surgical site during the procedure. The surgical team follows strict sterile techniques to minimize this risk, and you will be given antibiotics before and/or after the surgery to further reduce the chance of infection.

It's important to remember that these complications are generally uncommon, and the surgical team takes precautions to minimize them. Surgeons and healthcare providers who perform these procedures are experienced in port placement and are trained to manage any complications that may arise. Your healthcare provider will discuss these potential complications with you before the surgery and answer any questions or concerns you may have.

If you experience any unexpected symptoms or complications during the procedure, it's important to notify the surgical team immediately so that appropriate measures can be taken to address the situation.

3. Are there any specific factors that increase my risk of complications?

There are several factors that can increase the risk of complications during port placement surgery. While these factors may not guarantee complications will occur, they can contribute to a higher likelihood. Some specific factors that may increase the risk of complications include:

Medical Conditions: Certain underlying medical conditions can increase the risk of complications during surgery. Examples include bleeding disorders, clotting disorders, compromised immune system, heart or lung diseases, or other significant medical conditions that may impact healing or increase the risk of infection.

Obesity: Obesity can make the surgical procedure more challenging, increase the risk of surgical site infections, and affect wound healing. It can also make it more difficult to access and place the port in an optimal position.

Previous Surgeries or Scar Tissue: If you have undergone previous surgeries or have significant scar tissue in the area where the port will be placed, it may make the procedure more complex. Scar tissue can affect access, increase the risk of bleeding, and potentially increase the risk of injury to surrounding structures.

Anatomical Variations: Unusual anatomical structures or variations in blood vessel anatomy can increase the complexity of the procedure and may require additional precautions or techniques to ensure proper placement of the port.

Smoking: Smoking can have negative effects on wound healing and increase the risk of infection and complications. It is generally recommended to quit smoking or minimize smoking before and after surgery to reduce these risks.

Medications: Certain medications, such as anticoagulants or antiplatelet drugs, can increase the risk of bleeding during and after surgery. Your healthcare provider will provide guidance on whether you need to stop or adjust any medications prior to the surgery.

Allergies or Sensitivities: If you have known allergies or sensitivities to any materials used in the port or the medications administered through it, it is important to inform your healthcare provider before the procedure to minimize the risk of allergic reactions.

It is crucial to discuss any specific risk factors you may have with your healthcare provider prior to the surgery. They will evaluate your individual circumstances, take necessary precautions, and provide you with appropriate guidance to minimize the risk of complications. They may also modify their approach or techniques during the procedure to account for any risk factors and ensure your safety and well-being.

4. Can I die during the operation?

While every surgical procedure carries some inherent risks, including the potential for serious complications, the risk of death during a port placement operation is generally very low. Port placement surgery is considered a relatively safe procedure, and the occurrence of life-threatening complications leading to death is rare.

The surgical team takes various precautions to minimize risks and ensure patient safety. They follow strict sterile techniques, use imaging guidance during the procedure, and monitor vital signs throughout the surgery. Additionally, healthcare providers are trained to manage any potential complications that may arise during the procedure.

However, it's important to recognize that no surgical procedure is entirely without risk. Factors such as underlying medical conditions, unforeseen complications, or individual patient characteristics can contribute to a small level of risk. Some potential serious complications that could potentially be life-threatening include severe bleeding, infection, cardiac events, or adverse reactions to anesthesia. Nevertheless, these complications are rare, and healthcare providers work diligently to prevent and manage them.

It's crucial to discuss any concerns or fears you may have about the surgery with your healthcare provider. They can provide you with detailed information specific to your case, address your concerns, and help you understand the overall risk-benefit profile of the procedure. They will also inform you about any preoperative evaluations or tests that may be necessary to ensure your safety during the surgery.

5. Can any complications occur after the procedure?

Yes, there is a possibility of complications arising after a port placement procedure. While the majority of patients experience a smooth recovery without significant complications, it's important to

be aware of potential postoperative complications. Some possible complications that may occur after port placement include:

Infection: Infection is a potential complication that can develop at the surgical site or along the catheter. Signs of infection may include redness, swelling, warmth, tenderness, or drainage at the port insertion site. Fever, chills, or general malaise may also indicate an infection. Prompt medical attention is necessary if an infection is suspected.

Catheter-related Complications: The catheter itself can experience complications such as blockage, dislodgment, or damage. These complications may require intervention, such as catheter flushing, repositioning, or replacement.

Thrombosis: Blood clots can form within the catheter or in the veins surrounding the port. This can potentially lead to complications such as deep vein thrombosis (DVT) or pulmonary embolism (PE). Symptoms of thrombosis may include pain, swelling, or redness in the arm or chest area. If you experience any of these symptoms, it's important to seek medical attention promptly.

Extravasation: In rare cases, leakage of medication or fluids into the surrounding tissues can occur. This can cause localized pain, swelling, or tissue damage. If you notice any changes or discomfort at the port site during infusion, inform your healthcare provider immediately.

Skin Irritation or Breakdown: Prolonged use or friction from clothing or adhesive dressings can potentially irritate the skin around the port site. In some cases, skin breakdown or ulceration can occur. Proper care and monitoring of the port site can help prevent or manage skin-related complications.

Allergic Reactions: Some individuals may experience allergic reactions to the materials used in the port or the medications administered through it. Allergic reactions can range from mild skin irritation to more severe systemic reactions. If you develop any signs of an allergic reaction, such as rash, itching, swelling, or difficulty breathing, seek immediate medical attention.

It's important to follow postoperative care instructions provided by your healthcare provider, keep the port site clean and dry, and report any concerning symptoms or complications promptly. Regular follow-up appointments with your healthcare provider will also help monitor the function of the port and identify any potential complications early on.

Remember, while complications can occur, they are generally infrequent. Your healthcare provider will provide you with detailed instructions on postoperative care, signs of complications to watch for, and how to address them.

6. What are typical side effects of the procedure?

After a port placement procedure, you may experience some common side effects that are typically temporary and resolve as you recover. These side effects can vary from person to person but may include:

Discomfort or Pain: It is common to experience mild to moderate discomfort or soreness at the surgical site. This can be managed with over-the-counter pain medications as prescribed by your healthcare provider. The level of discomfort can vary, but it typically subsides within a few days to a week.

Swelling and Bruising: Swelling and bruising around the port insertion site are common and typically resolve over time. Applying ice packs or cold compresses to the area as directed by your healthcare provider can help reduce swelling and alleviate discomfort.

Limited Range of Motion: You may experience some limitation in the range of motion of your arm or shoulder on the side where the port is placed. This is temporary and usually improves as the surgical site heals.

Slight Scarring: A small scar will be present at the port insertion site. The scar will fade over time and become less noticeable.

Fatigue: After the procedure, you may feel fatigued or have reduced energy levels for a few days. This is a normal response to the surgical process and anesthesia.

Numbness or Tingling: Some individuals may experience temporary numbness or tingling sensations near the port site or along the distribution of nerves in the area. This is typically due to irritation or manipulation of nerves during the procedure and tends to resolve with time.

It's important to follow the postoperative care instructions provided by your healthcare provider, including keeping the port site clean and dry and avoiding any activities that may strain or irritate the area. If you experience any unusual or severe side effects, such as excessive pain, persistent swelling, signs of infection, or other concerns, it is important to contact your healthcare provider for further evaluation and guidance.

Remember, side effects and their duration can vary among individuals. Your healthcare provider will provide personalized instructions and support to help manage any side effects you may experience.

7. Will I lose blood during the procedure?

During a port placement procedure, it is possible to experience some blood loss. However, the amount of blood loss is typically minimal and is carefully controlled by the surgical team.

The surgeon will use techniques to minimize bleeding during the procedure, such as cauterization (using heat to seal blood vessels) or suturing to control any bleeding points. They will also monitor your blood pressure and vital signs throughout the surgery to ensure stability and address any issues promptly.

In most cases, the blood loss during a port placement procedure is well-tolerated and does not require blood transfusion or other interventions. However, it is important to note that individual circumstances can vary, and the amount of blood loss may depend on factors such as the complexity of the procedure, your overall health, and any underlying bleeding disorders you may have.

Your healthcare provider will discuss the potential risks and benefits of the procedure with you beforehand and address any concerns you may have about blood loss. If you have specific questions or if you are concerned about your individual risk, it is best to discuss them with your healthcare provider, who can provide you with more specific information based on your unique circumstances.

8. What structures can be injured during the procedure?

During a port placement procedure, there is a small risk of injury to surrounding structures. The surgical team takes precautions to minimize these risks, but it's important to be aware of potential structures that could be affected. Some of the structures that could be at risk of injury during the procedure include:

Blood vessels: The surgical team must navigate around blood vessels in the area where the port is being placed. Although rare, there is a risk of injury to blood vessels, which can lead to bleeding. Surgeons use imaging techniques, such as fluoroscopy or ultrasound, to guide the procedure and avoid damaging blood vessels.

Nerves: Nerves in the vicinity of the port insertion site could potentially be affected. Injury to nerves may cause temporary or, in rare cases, long-term sensory changes, such as numbness, tingling, or altered sensation in the surrounding areas. The surgical team takes care to minimize the risk of nerve injury, but it cannot be entirely eliminated.

Lung or pleura: If the port is placed in the upper chest area, there is a slight risk of accidentally puncturing the lung or pleura (the lining of the lung). This is known as pneumothorax. Surgeons use imaging guidance and follow proper techniques to minimize the risk of lung or pleural injury.

Organs: Depending on the location of the port placement, nearby organs, such as the heart, liver, or spleen, may be at risk of injury. However, these complications are extremely rare, and the surgical team takes precautions to avoid damaging organs during the procedure.

It's important to note that the occurrence of these complications is infrequent. Surgeons who perform port placement procedures are experienced and trained to navigate around these structures with precision. They use imaging techniques to guide the procedure and minimize the risk of injury.

Your healthcare provider will discuss the potential risks and benefits of the procedure with you beforehand. They will also evaluate your individual anatomy and medical history to assess any specific risks that may be applicable to your case. If you have concerns or questions about potential injuries to specific structures, it is best to discuss them with your healthcare provider, who can provide you with more specific information based on your unique circumstances.

9. What happens in a pneumothorax?

A pneumothorax is a condition that occurs when air accumulates in the space between the lung and the chest wall, known as the pleural space. In the context of a port placement procedure, a pneumothorax can occur if there is accidental puncture or injury to the lung or pleura during the surgery.

When a pneumothorax happens, the presence of air in the pleural space can disrupt the normal pressure balance, causing the lung to collapse partially or completely. This can lead to symptoms such as:

Sudden sharp or stabbing chest pain: The pain is typically on the side of the pneumothorax and may worsen with deep breathing or coughing.

Shortness of breath: As the lung collapses, it may become difficult to take deep breaths or breathe normally. You may feel breathless or experience a sense of tightness in the chest.

Rapid breathing: In response to the decreased lung function, your breathing rate may increase.

Cyanosis: In severe cases, where a significant portion of the lung is affected, a bluish discoloration of the lips, fingers, or skin may occur due to decreased oxygen levels.

If a pneumothorax occurs during a port placement procedure, the surgical team will promptly recognize the situation and take appropriate steps to manage it. They may use imaging techniques, such as fluoroscopy or X-rays, to confirm the diagnosis. The treatment for a pneumothorax may involve:

Observation: If the pneumothorax is small and not causing significant symptoms, the surgical team may choose to monitor it closely to see if it resolves on its own.

Chest tube insertion: In more severe cases or when the pneumothorax is causing symptoms or a significant lung collapse, a chest tube may be inserted to drain the air from the pleural space. This allows the lung to re-expand and relieves the symptoms.

Surgical intervention: In rare cases where other interventions are unsuccessful or the pneumothorax is severe, surgery may be required to repair the injured lung or pleura.

It's important to note that while a pneumothorax is a potential complication of a port placement procedure, it is relatively rare. The surgical team takes precautions to minimize the risk of this complication, such as using imaging guidance and following proper techniques. In the event of a pneumothorax, prompt recognition and appropriate management are crucial for ensuring patient safety and a favorable outcome.

10. How is a pneumothorax treated?

The treatment of a pneumothorax, which is the presence of air in the pleural space causing lung collapse, depends on the severity of the condition and the symptoms it is causing. The treatment options for a pneumothorax may include:

Observation: If the pneumothorax is small and not causing significant symptoms, a wait-and-see approach may be taken. Your healthcare provider will monitor the condition closely through regular check-ups and imaging, such as chest X-rays. In many cases, small pneumothoraces resolve on their own without further intervention.

Chest Tube Insertion: If the pneumothorax is large, causing significant lung collapse or symptoms such as shortness of breath, a chest tube may be inserted to remove the air from the pleural space. This is known as a chest tube thoracostomy. The chest tube is placed through a small incision in the chest wall and is connected to a drainage system. The tube helps re-expand the lung by allowing the trapped air to escape. Once the lung is re-expanded, the chest tube is typically left in place for a period of time to ensure complete resolution of the pneumothorax.

Needle Aspiration: In some cases, if the pneumothorax is small and causing symptoms, needle aspiration may be performed. A needle is inserted through the chest wall into the pleural space to remove the air. This procedure is typically used for smaller pneumothoraces and may be followed by observation or chest tube insertion, depending on the response.

Surgical Intervention: In rare cases or when other interventions are unsuccessful, surgery may be necessary to repair the source of the air leak or address underlying lung conditions contributing to recurrent pneumothoraces. Surgery may involve sealing or removing blebs or bullae (small air-filled sacs on the lung surface) or pleurodesis (creating adhesions between the lung and chest wall to prevent recurrence).

The specific treatment approach will depend on several factors, including the size of the pneumothorax, the severity of symptoms, your overall health, and the preference of your healthcare provider. It's important to consult with your healthcare provider to determine the most appropriate treatment option for your individual case.

Prompt medical attention is essential if you experience symptoms of a pneumothorax, such as sudden chest pain, shortness of breath, or rapid breathing. Timely diagnosis and appropriate treatment can help resolve the pneumothorax and prevent further complications.

11. How is a Port-Infection treated?

If a port becomes infected, prompt and appropriate treatment is necessary to prevent the spread of infection and potential complications. The treatment of a port infection typically involves a combination of the following approaches:

Antibiotics: Antibiotic therapy is a key component in treating a port infection. The specific choice of antibiotics will depend on the type of infection, the causative bacteria or organism, and the sensitivity of the bacteria to different antibiotics. In some cases, oral or intravenous antibiotics may be prescribed, while in more severe infections, intravenous antibiotics may be administered in a hospital setting.

Port Removal: In many cases of port infection, the infected port may need to be removed. This is done to eliminate the source of the infection and prevent further complications. The removal of the infected port is typically followed by a period of antibiotic treatment to ensure eradication of the infection.

Catheter Salvage: In some cases, if the infection is caught early and the catheter portion of the port is not involved, it may be possible to salvage the catheter while removing and replacing the infected port body. This approach is typically considered when the infection is localized to the port pocket and has not spread to the catheter.

Surgical Drainage: In certain situations where there is a collection of pus or abscess around the port pocket, surgical drainage may be necessary. This involves making an incision to drain the accumulated pus and removing any infected tissue.

Cultures and Sensitivity Testing: Cultures of the infected area, such as the port pocket or catheter tip, may be collected to identify the specific bacteria causing the infection. This helps guide antibiotic treatment by determining which antibiotics are most effective against the identified bacteria.

It's important to note that the management of port infections should be tailored to the individual patient and the specific circumstances. The treatment plan will be determined by your healthcare provider, who will consider factors such as the severity of the infection, your overall health, and the presence of any underlying conditions.

If you suspect that your port is infected or experience symptoms such as redness, swelling, warmth, pain, or drainage around the port site, it is crucial to contact your healthcare provider promptly. Early recognition and treatment of port infections are important to prevent further complications and ensure a successful outcome.

Postinterventional aftercare:

1. Will I have pain after the surgery?

It is common to experience some degree of pain or discomfort after a port placement surgery. The level of pain can vary depending on several factors, including individual pain tolerance, the specific surgical technique used, and any underlying health conditions. However, the pain is typically manageable with appropriate pain management strategies.

After the surgery, your healthcare provider will prescribe pain medications, such as oral analgesics or intravenous pain relievers, to help alleviate any post-operative pain. It is important to follow their instructions regarding pain medication usage, including dosages and timing.

In addition to pain medications, your healthcare provider may recommend other measures to help manage post-surgical pain, such as:

Local anesthesia: Local anesthesia may be used during the surgery to numb the area and provide immediate pain relief. This effect may continue for a few hours after the surgery.

Cold packs: Applying cold packs to the surgical area can help reduce swelling and provide temporary pain relief. Ensure that the cold pack is wrapped in a cloth or towel to protect the skin from direct contact with the cold.

Rest and elevation: Resting and keeping the surgical area elevated can help reduce pain and swelling.

Activity modification: Avoiding strenuous activities and movements that strain the surgical area can minimize post-operative pain.

It's important to communicate with your healthcare provider about your pain levels and any concerns you have. They can provide guidance on pain management techniques specific to your situation and adjust the pain medication regimen if needed.

While it is normal to experience some discomfort after the surgery, severe or worsening pain, or pain accompanied by other concerning symptoms, should be reported to your healthcare provider immediately, as it may indicate a complication or infection.

Remember to follow the post-operative care instructions provided by your healthcare team, as proper wound care and pain management are essential for a smooth recovery process.

2. How long does the local anaesthesia last?

The duration of local anesthesia can vary depending on several factors, including the specific anesthetic used, the site of administration, and individual variations in metabolism. In the case of port placement surgery, local anesthesia is typically used to numb the area around the surgical site.

Local anesthesia is designed to provide temporary pain relief by blocking nerve signals in the specific area where it is administered. The duration of its effect can range from a few hours to several hours, depending on the type and concentration of the anesthetic used. The most commonly used local anesthetics for surgical procedures include lidocaine and bupivacaine.

Lidocaine, for example, typically provides local anesthesia for a duration of approximately 1-2 hours. If a longer duration of anesthesia is desired, bupivacaine may be used, which can provide local pain relief for up to 6-8 hours. These timeframes are general estimates, and the actual duration of anesthesia can vary among individuals.

It's important to note that while local anesthesia can provide temporary pain relief immediately after the surgery, it will eventually wear off. As the effects of local anesthesia diminish, you may start to experience some discomfort or pain in the surgical area. This is why your healthcare provider will prescribe pain medications to manage post-operative pain as the local anesthesia wears off.

It's essential to follow your healthcare provider's instructions regarding pain management and take any prescribed pain medications as directed to ensure a comfortable recovery. If you have concerns about pain relief or the duration of anesthesia, it is best to discuss them with your healthcare provider, who can provide specific information based on the anesthesia plan for your surgery.

3. Can I drive a car after the surgery?

In general, it is not recommended to drive a car immediately after surgery, including port placement surgery. The effects of anesthesia, pain medications, and the physical stress of the surgery can impair your coordination, reflexes, and judgment, making it unsafe to operate a vehicle.

Post-surgery, it's important to prioritize rest and allow your body to recover. Driving too soon after surgery can pose risks not only to yourself but also to others on the road. Your ability to react quickly and make appropriate decisions may be compromised, increasing the likelihood of accidents.

The specific timeframe for when you can resume driving after surgery varies depending on factors such as the type of surgery, your individual recovery process, and any guidelines provided by your healthcare provider. It is best to consult with your healthcare provider or surgeon to get personalized advice regarding when it is safe for you to drive again.

They will consider your specific surgical procedure, the use of anesthesia and pain medications, your overall recovery progress, and any post-operative restrictions or precautions that may affect your ability to drive safely. They will provide guidance based on your individual case and ensure that you have fully recovered and are no longer experiencing any impairment before you resume driving.

Remember, it's crucial to prioritize your safety and the safety of others on the road. If you are unsure about your ability to drive or have concerns about the impact of surgery on your driving, it's best to wait until you have been cleared by your healthcare provider or until you feel confident in your ability to drive safely.

4. When can I start eating again?

The timing for resuming eating after surgery, including port placement surgery, can vary depending on the specific procedure performed, your individual recovery progress, and any specific instructions provided by your healthcare provider.

In most cases, after the surgery, you will initially be restricted from eating or drinking for a period of time to allow your body to recover from the effects of anesthesia and to ensure a safe transition from the surgical setting. This period of fasting is typically referred to as the "NPO" (nil per os) period.

The duration of the NPO period can vary, but it is commonly a few hours to overnight. Your healthcare provider or the surgical team will provide specific instructions regarding the length of the fasting period and when you can start consuming liquids and solids again.

Once the NPO period is over and you are cleared to resume eating, your healthcare provider will likely start you on a gradual progression of a clear liquid diet. Clear liquids are easily digested and include items such as water, broth, clear juices, and gelatin. This allows your digestive system to gradually resume normal function.

From there, your diet will typically progress to include soft or pureed foods and then advance to a regular solid diet based on your tolerance and the specific instructions from your healthcare provider. The timing for each diet progression can vary depending on your individual case and the recommendations of your healthcare team.

It is important to follow the dietary instructions provided by your healthcare provider or nutritionist to ensure proper healing and minimize any post-operative complications. If you have any concerns or questions about your diet after surgery, it is best to consult with your healthcare provider for personalized guidance and recommendations based on your specific needs and recovery progress.

5. When can the port be used?

   The timing for using the port after placement can vary depending on the specific surgical procedure, individual recovery progress, and the recommendations of your healthcare provider. Typically, there is a healing period after port placement during which the port site and surrounding tissues need time to heal and secure the port in place.

   Your healthcare provider will provide specific instructions regarding when the port can be used for various purposes, such as receiving medications, chemotherapy, or blood draws. In most cases, the port can be accessed and used once the healing process is well underway, and any surgical incisions have had time to heal and seal properly.

   It is common for a follow-up appointment to be scheduled after the surgery, during which your healthcare provider will assess the healing progress and determine when the port can be accessed and used. They may examine the port site, check for any signs of infection or complications, and ensure that the port is functioning correctly.

   It's important to follow your healthcare provider's instructions regarding the use of the port and any specific guidelines they provide. They will outline the appropriate timing and procedures for accessing and using the port, as well as any precautions or care instructions you need to follow to maintain the port's integrity and prevent complications.

   If you have any questions or concerns about using the port or the timeline for its usage, it is best to reach out to your healthcare provider for clarification and guidance. They will provide you with personalized information based on your specific case and ensure that you have a clear understanding of when and how to utilize the port effectively.

6. Will the port be visible after the surgery?

In most cases, the port itself will not be visibly protruding or visible on the surface of the skin after the surgery. The port is typically placed under the skin, usually in the chest or upper arm area, and is accessed using a needle through the skin.

The port is designed to have a low profile and is implanted in a way that minimizes its visibility. The port is often placed in a subcutaneous pocket, which helps to conceal it under the skin. The access point for the port, known as the septum or reservoir, is typically a small, self-sealing silicone or rubber disc that remains just beneath the skin surface.

After the surgical incision has healed, the port site may appear as a small, raised area or a subtle bump under the skin. However, it is usually not easily noticeable unless you specifically look for it. The port site may also be marked with a small scar, which can fade over time.

It's important to note that individual experiences may vary, and the visibility of the port can depend on factors such as body type, the specific placement site, and how well the healing progresses. If you have any concerns about the visibility of the port after surgery, it is best to discuss them with your healthcare provider, who can provide more specific information based on your individual case.

Remember, proper care and maintenance of the port, including keeping the area clean and following any instructions provided by your healthcare provider, can help minimize the risk of complications and ensure the longevity and effectiveness of the port.

7. Can I engage in sports with the port?

Engaging in sports or physical activities with a port is possible, but it is important to take certain precautions and follow the guidance provided by your healthcare provider.

The port is designed to withstand normal daily activities, including light exercise and physical movements. However, certain high-impact or contact sports may pose a risk of injury to the port or the surrounding area. Activities that involve direct impact to the port site, such as boxing, football, or rugby, should generally be avoided or approached with caution.

It is recommended to consult with your healthcare provider or surgeon to discuss the specific sports or physical activities you wish to engage in. They can provide personalized advice based on your individual situation, the type of port you have, and any restrictions or precautions that may be necessary.

In many cases, modifications can be made to accommodate your participation in sports. This may involve using protective padding or clothing to cover and cushion the port site during physical activity. Your healthcare provider may also provide specific guidelines on how to secure and protect the port during exercise or recommend alternative activities that are less likely to put the port at risk.

Additionally, it is important to listen to your body and pay attention to any discomfort, pain, or signs of strain during physical activity. If you experience any unusual symptoms or concerns related to the port or its functionality, it is crucial to stop the activity and consult with your healthcare provider.

Remember, the primary goal is to ensure the safety and integrity of the port while allowing you to maintain an active and healthy lifestyle. By working closely with your healthcare provider and following their recommendations, you can find a balance between participating in sports and protecting the port.

8. Will I have a scar?

It is possible to have a scar after port placement surgery, although the appearance and size of the scar can vary depending on factors such as the surgical technique used, individual healing capabilities, and the specific location of the incision.

During the port placement surgery, one or more small incisions are made to create a pocket under the skin where the port will be implanted. The incisions are typically small and closed with sutures or surgical adhesive.

After the surgery, the incisions will go through a healing process. Initially, the incision sites may appear as small, raised areas with redness and swelling. Over time, the incisions will gradually heal, and the scar tissue will form.

The appearance of the scar can vary from person to person. In some cases, the scar may be barely noticeable, appearing as a thin, faint line that blends with the surrounding skin. In other cases, the scar may be more visible, slightly raised, or have a different texture compared to the surrounding skin.

The scar may fade over time with proper care and healing, but it is important to note that individual healing processes and scar formation can vary. Taking care of the incision sites by keeping them clean, avoiding excessive sun exposure, and following any post-operative care instructions provided by your healthcare provider can help promote optimal healing and minimize the appearance of the scar.

If you have specific concerns about scarring or the appearance of the incision sites, it is best to discuss them with your healthcare provider. They can provide you with more personalized information based on your specific surgery and offer guidance on scar management techniques, such as scar massage or topical treatments, if necessary.

9. How should the port be cared for?

Proper care of the port is essential to maintain its functionality and minimize the risk of complications. Here are some general guidelines for port care:

Keep the port site clean: Wash your hands thoroughly before handling the port. Cleanse the area around the port with mild soap and water, or as instructed by your healthcare provider. Avoid using harsh chemicals or abrasive materials on the port site.

Avoid direct pressure or trauma: Be cautious when engaging in activities that may put direct pressure or trauma on the port site. Protect the area during physical activities by using padding or wearing protective clothing if recommended by your healthcare provider.

Avoid excessive pulling or tugging: Refrain from pulling or tugging on the port or the catheter connected to it. Be mindful of any movements or actions that could dislodge or damage the port.

Avoid submerging the port in water: Unless instructed otherwise by your healthcare provider, avoid submerging the port in water, such as during swimming or bathing. Take precautions to keep the port site dry and covered when exposed to water.

Regular flushing and maintenance: Follow the flushing and maintenance schedule prescribed by your healthcare provider. Flushing the port with a saline solution or heparin solution helps keep the catheter clear and prevents clot formation. Your healthcare provider will provide specific instructions on the frequency and technique for flushing the port.

Protect the port from infection: It is important to minimize the risk of infection. Avoid touching the port site unnecessarily, and maintain good hygiene when accessing the port or changing dressings. Follow any additional infection prevention measures provided by your healthcare provider.

Regular follow-up appointments: Attend all scheduled follow-up appointments with your healthcare provider. They will assess the port site, monitor its function, and address any concerns or complications that may arise.

It's important to note that the specific care instructions for your port may vary based on the type of port you have, the underlying condition being treated, and your healthcare provider's recommendations. Always follow the guidance provided by your healthcare team, ask questions if you are uncertain about any aspect of port care, and promptly report any signs of infection, discomfort, or issues with the port to your healthcare provider.

10. Will I feel the port under the skin?

After the port placement surgery, it is normal to have a sensation or awareness of the port under the skin. The port is typically implanted in a subcutaneous pocket, which means it is positioned beneath the layers of skin and tissue.

In the initial days or weeks following the surgery, you may feel some discomfort, tenderness, or mild pain in the area where the port is implanted. This is a common response to the surgical procedure and the presence of a foreign object within the body.

Over time, as the healing progresses and your body adjusts to the presence of the port, the discomfort and awareness of the port may diminish. However, it is possible that you may continue to have a sense of the port's presence or be able to feel it to some extent, especially when touching or pressing on the area directly above the port.

The extent to which you can feel the port can vary from person to person. Factors such as individual sensitivity, body composition, and the specific location of the port can influence the level of awareness or sensation.

If you have concerns about the sensations associated with the port or if you experience any significant pain, persistent discomfort, or changes in the way the port feels, it is important to communicate with your healthcare provider. They can evaluate the port and address any potential issues or complications that may arise.

It is worth noting that the presence of the port should not significantly hinder your daily activities or cause significant pain or discomfort. However, if you have any concerns or questions about the sensations related to the port, it is always best to consult with your healthcare provider for personalized guidance based on your specific circumstances.

11. Does it hurt to access the port?

The process of accessing the port for medication administration or blood draws typically involves inserting a needle into the port's septum or reservoir. While everyone's pain tolerance and experience may vary, accessing the port is generally well-tolerated by most individuals and is not overly painful.

Before accessing the port, a local anesthetic may be used to numb the area around the port site, which helps minimize discomfort during the procedure. The anesthetic is typically administered through a small needle, similar to the one used for accessing the port.

During the actual access of the port, you may feel some pressure or mild discomfort as the needle is inserted through the skin and into the port. However, the level of pain experienced is often minimal, especially with the use of local anesthesia.

It is important to communicate with your healthcare provider during the procedure and let them know if you experience any significant pain or discomfort. They can adjust the technique or provide additional measures to ensure your comfort.

If you have concerns about the potential pain associated with accessing the port or if you have a low pain threshold, you can discuss this with your healthcare provider. They can provide reassurance, offer pain management strategies, or explore alternative options to optimize your comfort during port access.

Remember, open communication with your healthcare team is crucial, as they can address any concerns you may have and work with you to ensure that the process of accessing the port is as comfortable as possible for you.

12. How long can I keep the port?

The duration for which a port can remain in place varies depending on individual circumstances, the underlying medical condition, and the specific treatment plan. In general, ports are intended to be long-term access devices, providing reliable access for medication administration and blood draws over an extended period.

Ports can typically remain in place for months to several years, depending on the individual's needs. The decision to remove the port is usually made in consultation with your healthcare provider and will depend on factors such as the completion of treatment, resolution of the underlying medical condition, or if the port is no longer needed.

Regular follow-up appointments with your healthcare provider will help assess the ongoing need for the port and monitor its functionality and condition. They will consider various factors, including your medical progress, treatment requirements, and the overall benefit-risk ratio, to determine the appropriate timing for removing the port.

If you have any concerns or questions about the duration of port placement in your specific case, it is best to discuss them with your healthcare provider. They can provide you with more personalized information and guidance based on your individual circumstances and treatment plan.

13. Can I undergo CT or MRI with the port?

The ability to undergo CT (computed tomography) or MRI (magnetic resonance imaging) scans with a port will depend on the specific type of port you have and its compatibility with these imaging techniques.

Many modern ports are designed to be compatible with CT and MRI scans, but it is important to inform the healthcare professionals performing the scan about the presence of the port beforehand. This allows them to take appropriate precautions and ensure the safety and functionality of the port during the imaging procedure.

In the case of CT scans, some ports may require specific protocols or adjustments to be made to the imaging parameters to minimize interference or artifacts caused by the port. The radiology department or healthcare provider can provide guidance on the specific requirements for your port.

For MRI scans, certain types of ports are MRI-compatible, while others may pose risks or have restrictions. MRI compatibility is typically determined by the materials used in the port's construction, such as the presence of any ferromagnetic components. If your port is MRI-compatible, it should be explicitly stated by the manufacturer. However, if your port is not MRI-compatible, alternative imaging options or protocols may need to be considered.

It is crucial to communicate with your healthcare provider, radiologist, or imaging technician to ensure that they are aware of your port and its specific characteristics. They can then determine the best course of action and make any necessary arrangements to accommodate the presence of the port during CT or MRI scans.

Remember, the decision to undergo CT or MRI scans with a port should be made in collaboration with your healthcare team, considering the potential benefits, risks, and any alternative options available for imaging.
