# Supplementary material for: Feasibility of GPT-3 and GPT-4 for in-Depth Patient Education Prior to Interventional Radiological Procedures: A Comparative Analysis
Source: Cardiovasc Intervent Radiol. 2023 Oct 23;47(2):245–50. doi: 10.1007/s00270-023-03563-2 (PMC10844465; doi:10.1007/s00270-023-03563-2)
Supplement: Supplementary file 2 — Supplementary file2 (PDF 340 kb) [file 270_2023_3563_MOESM2_ESM.pdf]

GPT-3 PTA

General Questions:

1. What is a PTA?

Percutaneous transluminal angioplasty (PTA) is a minimally invasive procedure used to treat narrowed or blocked blood vessels, typically in the arteries. It is commonly performed to improve blood flow in the coronary arteries (coronary angioplasty) or peripheral arteries (peripheral angioplasty).

During a PTA procedure, a thin, flexible tube called a catheter is inserted into a blood vessel, usually through a small incision in the groin or wrist. The catheter is guided through the blood vessels to the site of the blockage or narrowing. At the target location, a small balloon attached to the catheter is inflated to widen the artery and restore blood flow.

The inflation of the balloon compresses the plaque or fatty deposits against the arterial walls, effectively widening the vessel and improving blood flow. In some cases, a stent, a mesh-like tube, may be inserted and expanded at the site to provide structural support and prevent re-narrowing of the artery.

Overall, PTA is a minimally invasive alternative to traditional open surgery for treating arterial blockages, offering shorter recovery times, reduced risks, and avoiding the need for large surgical incisions.

2. How is a stenosis treated in PTA?
   A stenosis, which refers to the narrowing of an artery, is typically treated during PTA (percutaneous transluminal angioplasty) using the following steps:

   Catheter insertion: A thin catheter is inserted into the affected artery through a small incision or puncture site, usually in the groin or arm.

   Guidewire placement: A guidewire is threaded through the catheter and advanced to the site of the stenosis. The guidewire acts as a guide for the catheter and helps navigate through the blood vessels.

   Balloon inflation: Once the guidewire is in place, a balloon-tipped catheter is advanced over the guidewire to the stenosis. The balloon is positioned within the narrowed segment of the artery.

   Balloon inflation: The balloon is inflated, exerting pressure on the plaque and the arterial walls. This pressure compresses the plaque, widening the artery, and restoring blood flow.

   Balloon deflation and removal: After an adequate period of inflation, the balloon is deflated and removed from the artery.

Possible stent placement: In some cases, a stent may be inserted after the balloon inflation to help maintain the newly widened artery. The stent is a mesh-like tube made of metal or other materials that acts as a scaffold to hold the artery open. It is placed in the treated area and expanded using a balloon.

Post-procedure assessment: After the PTA procedure, the blood flow is assessed using imaging techniques, such as angiography, to ensure that the stenosis has been adequately treated and blood flow has improved.

It's important to note that the specific steps and techniques used during PTA may vary depending on the location and complexity of the stenosis, as well as the judgment of the treating physician. Additionally, the decision to place a stent is based on the characteristics of the stenosis and individual patient factors.

3. What is a stent?

A stent is a medical device designed to help keep a narrowed or blocked artery open and maintain proper blood flow. It is commonly used in the treatment of conditions like coronary artery disease (CAD), peripheral arterial disease (PAD), and other vascular conditions. Stents are typically made of metal, such as stainless steel or cobalt-chromium alloys, or they can be coated with medications (drug-eluting stents) that help prevent the re-narrowing of the artery.

When a stent is used in a medical procedure, it is usually in the form of a mesh-like tube. The stent is delivered to the affected area using a catheter, which is inserted into the artery through a small incision or puncture site. The catheter is guided to the narrowed or blocked segment of the artery, and the stent is positioned in that area.

Once in place, the stent is expanded using a balloon, a process called balloon angioplasty. The balloon inflates and presses the stent against the arterial walls, causing it to expand and push the plaque or blockage against the artery wall, effectively widening the artery. Afterward, the balloon is deflated and removed, leaving the stent in place to hold the artery open.

Stents can be either bare metal stents (BMS) or drug-eluting stents (DES). Bare metal stents are made solely of metal and serve as a scaffold to keep the artery open. Drug-eluting stents, on the other hand, have a special coating that slowly releases medications (typically anti-proliferative drugs) to prevent the re-narrowing of the artery. This drug release helps reduce the risk of restenosis, which is the reoccurrence of narrowing in the treated artery.

Stents can effectively improve blood flow, relieve symptoms, and prevent further complications in conditions where arteries are narrowed or blocked. However, it's important to note that stents are not suitable for all patients or all types of blockages. The decision to use a stent depends on factors such as the location, length, and severity of the blockage, as well as the overall health of the individual. A healthcare professional will evaluate each case and determine the most appropriate treatment plan, which may or may not include stent placement.

4. What can be treated with a PTA?

Percutaneous transluminal angioplasty (PTA) can be used to treat various conditions caused by narrowed or blocked blood vessels, particularly in the arteries. Here are some conditions that can be treated with PTA:

Coronary Artery Disease: PTA, also known as coronary angioplasty, is commonly used to treat coronary artery disease, which involves the narrowing of the coronary arteries supplying blood to the heart muscle. PTA can improve blood flow to the heart, relieve chest pain (angina), and in some cases, it may be performed during a heart attack to restore blood flow.

Peripheral Artery Disease (PAD): PTA can be used to treat peripheral artery disease, a condition where arteries in the legs, arms, or other peripheral areas become narrowed or blocked. PTA in peripheral arteries can relieve symptoms like leg pain or cramping during exercise (claudication) and improve blood circulation.

Renal Artery Stenosis: This condition involves narrowing of the arteries that supply blood to the kidneys. PTA can be performed to open up the renal arteries and restore proper blood flow to the kidneys, which can help manage high blood pressure or improve kidney function.

Carotid Artery Stenosis: Carotid arteries are the major blood vessels that supply blood to the brain. When these arteries become narrowed or blocked, it can increase the risk of stroke. PTA with or without stenting can be used to open up the carotid arteries and reduce the risk of stroke.

Access Site Stenosis: PTA can also be used to treat stenosis or blockages that develop at the site where dialysis or other catheters are inserted into blood vessels for long-term access.

It's important to note that PTA is typically performed by a specialized interventional radiologist or an interventional cardiologist. The specific applicability of PTA depends on the individual patient's condition, the location and severity of the blockage, and other factors, which are assessed by the healthcare professional to determine the most appropriate treatment approach.

5. Can my peripheral arterial disease (pAD) be improved through a PTA?

   Yes, percutaneous transluminal angioplasty (PTA) can often improve peripheral arterial disease (PAD) by restoring blood flow to the affected areas. PAD is a condition where there is a narrowing or blockage of the arteries that supply blood to the legs, arms, or other peripheral areas.

   During a PTA procedure for PAD, a catheter with a small balloon at its tip is guided to the site of the blockage or narrowing in the affected artery. The balloon is then inflated, which compresses the plaque or fatty deposits against the arterial walls, widening the vessel and improving blood flow. In some cases, a stent (a mesh-like tube) may also be placed at the site to provide structural support and prevent re-narrowing of the artery.

PTA for PAD can alleviate symptoms such as leg pain or cramping during exercise (claudication) by improving circulation to the affected limb. It can also help heal non-healing wounds or ulcers on the legs or feet caused by poor blood flow.

It's important to note that the effectiveness of PTA in treating PAD can vary depending on factors such as the location and severity of the blockage, the extent of underlying arterial disease, and the overall health of the patient. In some cases, additional treatments or interventions may be necessary to manage PAD, such as lifestyle modifications (e.g., exercise, smoking cessation) or medications. The decision to undergo PTA or any other treatment for PAD should be made in consultation with a healthcare professional who can assess your specific condition and provide personalized recommendations.

6. Can my peripheral arterial disease (pAD) be cured through a PTA?

While percutaneous transluminal angioplasty (PTA) can effectively improve symptoms and restore blood flow in peripheral arterial disease (PAD), it is important to understand that PAD is a chronic condition and cannot be completely cured by a single PTA procedure.

PAD is caused by atherosclerosis, a systemic disease characterized by the build-up of plaque within the arteries. While PTA can open narrowed or blocked arteries and provide relief from symptoms, it does not address the underlying cause of atherosclerosis or prevent the development of new blockages in other arteries.

Furthermore, even after a successful PTA procedure, there is a possibility of restenosis, which is the re-narrowing of the treated artery over time. Restenosis can occur in a certain percentage of patients, requiring additional interventions or treatments.

To effectively manage PAD, a comprehensive approach is typically employed. This may include lifestyle modifications such as regular exercise, a healthy diet, smoking cessation, and controlling underlying risk factors like high blood pressure, high cholesterol, and diabetes. Medications may also be prescribed to manage symptoms and reduce the risk of cardiovascular complications.

In some cases, PTA with the placement of a stent may be combined with other interventions or procedures, such as atherectomy (removal of plaque) or bypass surgery, depending on the severity and location of the blockages.

The goal of PAD management is to alleviate symptoms, improve quality of life, prevent complications, and slow down the progression of the disease. Regular follow-up with healthcare professionals is essential to monitor the condition, adjust treatment plans as needed, and provide ongoing care to manage PAD effectively.

7. When is a PTA performed?

Percutaneous transluminal angioplasty (PTA) is performed when there is a need to treat narrowed or blocked blood vessels in specific clinical scenarios. The timing of a PTA procedure depends on various factors, including the patient's symptoms, the severity of the blockage, and the overall clinical judgment of the healthcare provider. Here are some situations where a PTA may be performed:

Symptomatic Blockages: PTA is commonly performed when a patient exhibits symptoms related to reduced blood flow caused by arterial blockages. For example, in coronary artery disease, PTA may be considered if a patient experiences angina (chest pain) that is not adequately controlled with medication or if they have a heart attack due to a blocked coronary artery.

Non-emergent Cases: In some cases, PTA may be performed in a planned or non-emergent manner. For example, if a patient with peripheral artery disease (PAD) experiences symptoms such as leg pain or difficulty walking (claudication), and conservative treatments have not been effective, a PTA procedure may be scheduled to improve blood flow and alleviate symptoms.

Acute Emergencies: PTA can also be performed urgently or as an emergency procedure. For instance, in the case of a severe heart attack (ST-segment elevation myocardial infarction, or STEMI), PTA may be performed immediately to restore blood flow to the affected coronary artery and minimize damage to the heart muscle.

Surveillance and Maintenance: In some cases, PTA may be performed as part of ongoing surveillance and maintenance of previously treated arterial blockages. For example, in patients who have undergone PTA with stenting, follow-up procedures may be scheduled to monitor stent patency, assess any re-narrowing (restenosis), or treat any recurrent blockages that may develop over time.

The decision to perform a PTA is based on a comprehensive evaluation of the patient's medical history, diagnostic tests (such as angiography or imaging studies), and clinical presentation. The healthcare provider will determine the appropriate timing and necessity of the procedure based on individual patient factors and the severity of the condition.

8. What are the alternatives to a PTA in pAD?

There are alternative treatment options available for peripheral arterial disease (PAD) if percutaneous transluminal angioplasty (PTA) is not suitable or effective for a particular patient. Here are some alternatives:

Medications: Certain medications can be prescribed to manage the symptoms and underlying conditions associated with PAD. These may include antiplatelet drugs (such as aspirin or clopidogrel) to reduce the risk of blood clots, cholesterol-lowering medications (such as statins) to control high cholesterol levels, and medications to control blood pressure or manage diabetes.

Lifestyle Modifications: Making lifestyle changes is an important aspect of managing PAD. This can include regular exercise, such as supervised walking programs, as well as quitting smoking, maintaining a healthy diet, and managing other risk factors like obesity and diabetes.

Supervised Exercise Programs: Participating in supervised exercise programs, such as structured walking programs, can help improve symptoms, increase walking distance, and promote overall cardiovascular health in individuals with PAD.

Endovascular Procedures: In addition to PTA, there are other endovascular procedures that can be considered for the treatment of PAD. These include:

a. Atherectomy: This procedure involves the removal of plaque from the artery using specialized devices such as rotating blades, lasers, or cutting balloons.

b. Stent Placement: In cases where PTA alone may not be sufficient to keep the artery open, a stent—a mesh-like tube—can be placed to provide structural support and maintain vessel patency.

c. Drug-Coated Balloons: These balloons are coated with medication that is released at the site of the blockage during angioplasty, reducing the risk of restenosis.

Bypass Surgery: In some cases, when the blockages are severe or extensive, bypass surgery may be recommended. This involves creating a new pathway for blood flow by using a graft to bypass the blocked artery.

The choice of alternative treatments depends on various factors, including the location and severity of the blockages, the overall health of the patient, and the expertise of the healthcare provider. It's important to consult with a healthcare professional who specializes in the treatment of PAD to determine the most appropriate alternative treatment option for your specific condition.

9. What are the success rates of a PTA in pAD?

The success rates of percutaneous transluminal angioplasty (PTA) in peripheral arterial disease (PAD) can vary depending on several factors, including the location and severity of the disease, the characteristics of the treated artery, and individual patient factors. It's important to note that success rates can be defined differently based on various parameters, such as immediate procedural success, short-term outcomes, or long-term outcomes. Here are some general insights:

Immediate procedural success: PTA can often achieve immediate procedural success, meaning the balloon angioplasty successfully widens the narrowed artery and restores blood flow. This is typically assessed during the procedure itself, and the success rate can be quite high, ranging from 80% to 95% or higher in certain cases.

Short-term outcomes: In terms of short-term outcomes, PTA can effectively relieve symptoms associated with PAD, such as leg pain and cramping during physical activity. Immediate improvements in blood flow and symptom relief are commonly observed. However, it's worth noting that the re-narrowing of the treated artery, known as restenosis, can occur in some cases, leading to recurrent symptoms. The use of adjunctive techniques like stenting can help reduce the risk of restenosis.

Long-term outcomes: The long-term success rates of PTA in PAD can be influenced by various factors, including the extent of the underlying disease, the presence of other risk factors (e.g., smoking, diabetes), and lifestyle modifications. Long-term studies have reported mixed outcomes, with some indicating durable results and others showing a higher likelihood of restenosis over time. The overall success rates beyond the immediate procedural success can range from 60% to 80% in terms of sustained symptom relief and avoidance of major adverse limb events.

It's important to emphasize that each case is unique, and the success of PTA depends on individual patient characteristics and the specific circumstances of the disease. Additionally, a comprehensive treatment approach that includes lifestyle modifications, medication management, and ongoing medical care can play a significant role in improving long-term outcomes for individuals with PAD.

When considering PTA or any other medical procedure, it's crucial to consult with a healthcare professional who can provide personalized advice and discuss the potential benefits, risks, and expected outcomes based on your specific situation.

10. What are contraindications for a PTA?

While percutaneous transluminal angioplasty (PTA) is generally a safe and effective procedure for treating peripheral arterial disease (PAD), there are certain contraindications and factors that may make PTA unsuitable for some individuals. The contraindications for PTA can include:

Severe allergy or adverse reaction to the contrast dye: PTA often involves the use of contrast dye during imaging procedures. If a person has a known severe allergy or has previously experienced a life-threatening reaction to contrast dye, alternative treatment options may need to be considered.

Active infection: If there is an active infection present in the area being treated or systemically, it may be necessary to postpone the PTA procedure until the infection is adequately controlled or resolved.

Severe kidney disease: PTA requires the use of contrast dye, which can potentially affect kidney function. If an individual has severe kidney disease or impaired renal function, alternative strategies may be necessary to minimize the risk of further kidney damage.

Inability to cooperate or lie still during the procedure: PTA involves the use of X-ray imaging and requires the patient to lie still during the procedure. Individuals who are unable to cooperate or lie still due to cognitive impairment, agitation, or other factors may not be suitable candidates for PTA.

Advanced arterial disease or extensive calcification: In some cases, the presence of advanced arterial disease or extensive calcification in the treated artery can make PTA technically challenging or less effective. Alternative treatment options, such as surgical bypass grafting, may be considered in such situations.

It's important to note that contraindications may vary depending on individual patient factors, the specific characteristics of the disease, and the judgment of the treating physician. A comprehensive evaluation by a healthcare professional is essential to determine the suitability of PTA and to explore alternative treatment options if needed.

It's always recommended to consult with a healthcare provider who can assess your specific situation, consider your medical history, and provide personalized advice regarding the potential risks, benefits, and alternatives associated with PTA.

Preparation and Procedure:

1. How should I prepare for my percutaneous transluminal angioplasty (PTA)?

To prepare for your percutaneous transluminal angioplasty (PTA) procedure, it is important to follow the instructions provided by your healthcare provider. Here are some general guidelines that may apply:

Medical evaluation: Your healthcare provider will perform a thorough medical evaluation to assess your overall health and the severity of your peripheral arterial disease (PAD). They may review your medical history, conduct physical examinations, and order necessary tests, such as blood work or imaging studies, to gather important information before the procedure.

Medications: Inform your healthcare provider about any medications you are currently taking, including prescription drugs, over-the-counter medications, and supplements. They will provide specific instructions about which medications you should continue or discontinue before the procedure. In some cases, you may need to temporarily stop medications that can increase the risk of bleeding or interact with the contrast dye used during the procedure.

Fasting: You may be instructed to fast for a certain period of time before the procedure. Typically, you will need to refrain from eating or drinking anything (including water) for a specified duration prior to the PTA. Fasting is necessary to ensure an empty stomach, which reduces the risk of complications during the procedure, especially if sedation or anesthesia is involved.

Clothing and personal items: Wear comfortable clothing and avoid wearing jewelry, watches, or other accessories that may interfere with the procedure. You may be provided with a hospital gown to wear during the PTA. Additionally, it's advisable to bring a list of your medications, any relevant medical documents, and insurance information to the hospital or clinic.

Transportation: Arrange for someone to accompany you to the hospital or clinic and drive you home afterward. PTA procedures often involve the use of sedation or anesthesia, which can temporarily impair your ability to drive and make it unsafe to operate a vehicle. Having a support person with you is important for your safety and well-being.

Clarify doubts: If you have any concerns or questions about the PTA procedure or the pre-procedure instructions, it's crucial to communicate with your healthcare provider. They will be able to provide you with the necessary information and address any concerns you may have.

Remember, these are general guidelines, and specific instructions may vary depending on your individual case and the protocols followed by your healthcare provider. Always follow the instructions provided by your healthcare team to ensure the best possible preparation for your PTA procedure.

2. Will I need to fast before the procedure?

Fasting requirements before a percutaneous transluminal angioplasty (PTA) procedure can vary depending on several factors, including the specific instructions provided by your healthcare provider and the planned timing of the procedure. It's essential to follow the specific fasting guidelines given to you by your healthcare team. Here are some general considerations:

Fasting instructions: Your healthcare provider will inform you about the fasting requirements for your PTA procedure. In many cases, you will be instructed to refrain from eating or drinking anything (including water) for a specified duration before the procedure. This fasting period ensures an empty stomach, reducing the risk of complications during the procedure, particularly if sedation or anesthesia is involved.

Duration of fasting: The duration of fasting may vary depending on the specific instructions provided. Commonly, you may be asked to fast for at least six hours or more before the scheduled procedure. However, it's important to follow the exact instructions provided by your healthcare provider, as they may have specific protocols in place based on your individual case and other factors.

Medication guidelines: Your healthcare provider will provide instructions regarding the medications you should or should not take before the procedure. In some cases, you may be asked to temporarily discontinue certain medications before the PTA. However, it's crucial to consult your healthcare provider about medication management during the fasting period, as they can provide specific guidance based on your medical condition and prescribed medications.

Clear liquids: It's common for healthcare providers to allow the consumption of clear liquids up to a certain time before the procedure. Clear liquids are usually defined as transparent liquids that do not contain solid particles, such as water, clear juices without pulp, or black coffee without creamer. However, it's important to follow the specific instructions given by your healthcare provider, as they may have variations in the fasting requirements.

Communication with your healthcare provider: If you have any doubts or concerns about fasting before the PTA procedure, it's essential to communicate with your healthcare provider. They can provide you with the precise instructions based on your specific case and address any questions you may have.

To ensure the best preparation for your PTA procedure, carefully follow the fasting instructions provided by your healthcare provider. Adhering to these guidelines will help minimize the risk of complications and ensure the procedure can be performed safely and effectively.

3. Should I continue taking my medications before PTA?

Whether or not you should continue taking your medications before a percutaneous transluminal angioplasty (PTA) procedure depends on the specific medications you are taking and the instructions provided by your healthcare provider. Here are some general considerations:

Communicate with your healthcare provider: It's crucial to inform your healthcare provider about all the medications you are currently taking, including prescription drugs, over-the-counter medications, and supplements. Your healthcare provider will review your medication list and provide specific instructions regarding which medications you should continue or discontinue before the PTA procedure.

Anticoagulant and antiplatelet medications: Medications such as aspirin, clopidogrel (Plavix), or other anticoagulants or antiplatelet drugs are commonly prescribed to prevent blood clots. These medications are often continued before PTA procedures to reduce the risk of clot formation during or after the procedure. However, in some cases, your healthcare provider may adjust the dose or provide specific instructions regarding these medications based on your individual situation.

Medications that may increase bleeding risk: Certain medications, such as nonsteroidal anti-inflammatory drugs (NSAIDs) like ibuprofen or blood-thinning medications like warfarin, may increase the risk of bleeding during the PTA procedure. Your healthcare provider may ask you to temporarily discontinue these medications or adjust their dosage before the procedure to minimize the risk of bleeding complications.

Other medications: The impact of other medications on the PTA procedure can vary. Your healthcare provider will assess the potential interactions or effects of your medications and provide instructions accordingly. It's important to follow their recommendations regarding the timing and dosage of your medications before the procedure.

Discuss concerns and questions: If you have any concerns or questions about the medications you are currently taking and how they may affect the PTA procedure, it's essential to communicate with your healthcare provider. They can provide you with personalized guidance and clarify any doubts you may have.

Always follow the specific instructions provided by your healthcare provider regarding medication management before the PTA procedure. It's important to balance the benefits of continuing certain medications with the potential risks and interactions they may pose during the procedure. Your healthcare provider will consider your individual case and provide the most appropriate recommendations for your specific situation.

4. Will I be awake during the procedure?

The level of consciousness or sedation during a percutaneous transluminal angioplasty (PTA) procedure can vary depending on several factors, including the complexity of the procedure, the preferences of the healthcare team, and the patient's overall health. Here are the potential options:

Local anesthesia: In some cases, a PTA procedure can be performed using local anesthesia. This means that only the specific area being treated will be numbed, and you will be awake and conscious throughout the procedure. You may feel some pressure or discomfort, but the local anesthesia helps minimize pain. Your healthcare team will provide instructions and communicate with you during the procedure.

Conscious sedation: Conscious sedation involves the use of medications to help you relax and feel drowsy during the procedure. With conscious sedation, you will still be able to respond to commands and communicate, but you may not remember the details of the procedure. This sedation can make the experience more comfortable and reduce anxiety.

General anesthesia: In certain cases, particularly for more complex or lengthy procedures, general anesthesia may be used. General anesthesia induces a state of complete unconsciousness, and you will not be aware or remember anything during the procedure. This type of anesthesia requires the presence of an anesthesiologist or a certified anesthetist who will monitor your vital signs and administer the medications.

The choice of anesthesia or sedation method depends on various factors, including the nature of the procedure, your medical condition, and your preferences. Your healthcare provider will discuss the anesthesia or sedation options with you, explain the associated risks and benefits, and help determine the most appropriate approach for your PTA procedure.

It's important to note that regardless of the level of consciousness during the procedure, a healthcare team will be present to monitor your vital signs, ensure your comfort, and address any concerns or

questions you may have. They will provide appropriate pain management and take all necessary measures to ensure your safety throughout the procedure.

5. How is the procedure performed in patients with peripheral arterial disease?

The procedure for percutaneous transluminal angioplasty (PTA) in patients with peripheral arterial disease (PAD) involves the following general steps:

Pre-procedure preparation: Before the procedure, you will be taken to the angiography suite or a similar specialized room. You may be asked to change into a hospital gown and lie on an examination table.

Local anesthesia or sedation: Depending on the complexity of the procedure and your specific case, you may receive local anesthesia to numb the area being treated or conscious sedation to help you relax and feel drowsy during the procedure.

Arterial access: The healthcare provider will identify an appropriate artery to access the affected area. Typically, the access site is in the groin or sometimes in the wrist or upper arm. The area will be cleaned and sterilized, and a local anesthetic will be administered to numb the area.

Guidewire and catheter placement: A small incision is made in the skin, and a needle is inserted into the artery. A guidewire is then threaded through the needle and advanced to the site of the blockage or narrowing. Following the guidewire, a catheter is inserted and guided to the affected area using X-ray imaging guidance.

Angiography: Once the catheter is in position, a contrast dye is injected through the catheter, and X-ray images (angiograms) are taken. These images help visualize the blockage or narrowing in the artery and guide the treatment.

Balloon angioplasty: To treat the stenosis (narrowing) in the artery, a balloon-tipped catheter is advanced to the site of the blockage. The balloon is inflated, which pushes against the plaque or deposits in the artery, widening the vessel and improving blood flow. The balloon is then deflated and removed.

Stent placement (if necessary): In some cases, a stent may be required to help keep the artery open and maintain blood flow. A stent is a small metal mesh tube that is placed in the treated area. It provides structural support and helps prevent the artery from narrowing again (restenosis).

Post-procedure evaluation and closure: After the balloon angioplasty and/or stent placement, the healthcare provider will evaluate the blood flow and the results of the procedure using angiography. If there are no further issues, the catheter is removed, and pressure is applied to the access site to

prevent bleeding. In some cases, a closure device or manual pressure may be used to seal the access site.

Recovery and observation: You will be moved to a recovery area, where healthcare professionals will monitor your vital signs and the access site. Depending on the specific circumstances, you may need to stay in the hospital for observation or be discharged the same day with instructions for post-procedure care and follow-up.

It's important to note that the specific details of the procedure may vary based on the individual patient and the characteristics of the peripheral arterial disease being treated. The healthcare team will tailor the procedure to your specific needs and provide you with detailed instructions and information before and after the PTA procedure.

6. How long will the procedure take?

The duration of a percutaneous transluminal angioplasty (PTA) procedure can vary depending on several factors, including the complexity of the case, the number of arterial lesions being treated, and the specific techniques employed. Generally, the procedure duration can range from 30 minutes to a few hours. Here are some factors that can influence the overall procedure time:

Complexity of the disease: The severity and complexity of the peripheral arterial disease (PAD) can impact the procedure duration. If there are multiple arterial lesions or if the disease is advanced, the procedure may take longer to address each area of stenosis or blockage.

Number of treated areas: If there are multiple areas requiring treatment, such as different arteries or segments of the same artery, the procedure may take longer as each site needs to be addressed individually.

Additional interventions: Sometimes, additional procedures may be performed during the PTA. For example, if a stent placement is necessary or if there are complications that need to be addressed, it can extend the procedure time.

Operator expertise: The experience and expertise of the healthcare provider performing the PTA can also influence the procedure duration. Skilled operators may be able to complete the procedure more efficiently.

Patient factors: Individual patient factors, such as the patient's anatomy, overall health, and response to the procedure, can also impact the duration. Each patient's case is unique, and the procedure time may be influenced by their specific circumstances.

It's important to note that while the procedure itself may take a relatively short amount of time, additional time may be required for pre-procedure preparations and post-procedure recovery and observation. The healthcare team will provide you with more accurate estimates of the procedure duration based on your specific case.

During the PTA procedure, you will be under the care and observation of healthcare professionals who will ensure your safety and comfort. They will provide updates and information throughout the procedure to keep you informed about the progress.

7. Will I feel any pain during PTA?

During a percutaneous transluminal angioplasty (PTA) procedure, measures are taken to minimize pain and discomfort as much as possible. The level of pain or discomfort experienced can vary depending on several factors, including the specific techniques used, the individual's pain tolerance, and the areas being treated. Here's what you can generally expect:

Local anesthesia: Before the PTA procedure begins, a local anesthetic will be administered to numb the area where the catheter is inserted. This helps minimize pain or discomfort at the access site. You may feel a brief sting or pinch from the injection of the anesthetic, but it should quickly subside as the numbing effect takes place.

Pressure and sensation: During the procedure, you may feel some pressure or a sensation of movement as the catheter is guided through the blood vessels to the treatment site. You might also experience a warm or flushing sensation when the contrast dye is injected.

Balloon inflation: When the balloon-tipped catheter is inflated to widen the narrowed artery, you may experience a sensation of pressure or discomfort at the treatment site. It is common to feel a temporary sensation of tightness or pain during this part of the procedure.

Sedation or pain relief: Depending on the complexity of the procedure and your individual needs, you may be given conscious sedation or pain-relieving medications to help you relax and manage any discomfort. Conscious sedation can make you feel drowsy and less aware of the procedure, reducing any potential pain or anxiety.

Communication with the healthcare team: It's essential to communicate any discomfort or pain you experience during the procedure to the healthcare team. They can adjust pain medications, provide reassurance, and take appropriate measures to address your concerns and ensure your comfort.

It's important to note that every individual's experience during a PTA procedure can vary. Some individuals may experience minimal discomfort, while others may have a higher sensitivity to pain. The healthcare team will make efforts to minimize pain and ensure your well-being throughout the procedure. If at any time during the procedure you experience excessive pain or discomfort, inform the healthcare team immediately so that appropriate measures can be taken.

Overall, the goal of the healthcare team is to perform the PTA procedure safely and with minimal pain or discomfort. They will do their best to ensure your comfort and address any concerns you may have.

8. What happens if a complication is discovered during the procedure?

During a percutaneous transluminal angioplasty (PTA) procedure, complications can rarely occur. The healthcare team performing the procedure is trained to recognize and manage these complications promptly. If a complication is discovered during the procedure, the following actions may be taken:

Immediate assessment: The healthcare team will carefully assess the nature and severity of the complication. This may involve additional imaging, such as angiography or other diagnostic tests, to gather more information about the situation.

Decision-making: Based on the assessment of the complication, the healthcare team will determine the appropriate course of action. The specific actions taken will depend on the nature of the complication and may vary from case to case.

Intervention or correction: The healthcare team will take steps to address the complication and mitigate any risks to the patient. The intervention can range from adjusting the procedure technique, administering medications, or deploying additional medical devices to manage the situation.

Communication and informed consent: If a significant complication arises that necessitates a change in the treatment plan or introduces additional risks, the healthcare team will communicate with the patient or their representative. They will provide information about the complication, explain the available options, and obtain informed consent before proceeding with any necessary interventions.

Specialist consultation: In some cases, the healthcare team may seek the assistance of specialists, such as interventional radiologists, vascular surgeons, or other relevant experts, to provide additional guidance and expertise in managing the complication.

Documentation and monitoring: The details of the complication, the actions taken, and the patient's response will be documented in the medical records. Close monitoring of the patient's condition will continue to ensure appropriate management and to detect any further complications.

It's important to note that while complications during a PTA procedure are relatively rare, they can potentially occur due to factors such as underlying disease severity, anatomy, or unforeseen events. The healthcare team is prepared to handle such situations promptly and effectively to ensure the best possible outcome for the patient.

If you have any concerns or questions about potential complications or how they would be managed during the PTA procedure, it's important to discuss them with your healthcare provider. They can provide you with specific information based on your individual case and address any concerns you may have.

9. Will I be able to feel or sense anything in my leg during the procedure?

During a percutaneous transluminal angioplasty (PTA) procedure, it is common for patients to feel sensations in their leg, particularly in the area where the catheter is being manipulated. Here are some sensations you may experience:

Pressure and movement: As the catheter is advanced through the blood vessels towards the treatment site, you may feel a sensation of pressure or movement in your leg. This is a normal sensation caused by the catheter navigating through the arteries.

Warmth or flushing: When the contrast dye is injected to visualize the blood vessels during angiography, you may experience a temporary warm or flushing sensation in your leg. This sensation is normal and should subside shortly after the dye is injected.

Discomfort during balloon inflation: If balloon angioplasty is performed to widen the narrowed artery, you may feel some discomfort or pressure at the treatment site when the balloon is inflated. This sensation is temporary and should resolve once the balloon is deflated.

Numbness due to local anesthesia: Before the procedure begins, local anesthesia will be administered to numb the area where the catheter is inserted. This can result in temporary numbness or reduced sensation in the immediate vicinity of the access site.

It's important to communicate any sensations you experience during the procedure to the healthcare team. They can provide reassurance and adjust pain medications or techniques to ensure your comfort. If at any point during the procedure you experience severe pain or unusual sensations, it is important to inform the healthcare team immediately.

While you may feel some sensations during the procedure, the goal of the healthcare team is to minimize any discomfort and ensure your well-being throughout the PTA procedure. They will take measures to keep you as comfortable as possible and address any concerns you may have.

10. What type of anesthesia or sedation can be used during PTA?

During a percutaneous transluminal angioplasty (PTA) procedure, different types of anesthesia or sedation can be used depending on the complexity of the procedure, patient preferences, and the healthcare team's recommendations. Here are the common options:

Local anesthesia: Local anesthesia involves the injection of an anesthetic medication into the area where the catheter is inserted (usually the groin or sometimes the wrist or upper arm). It numbs the specific area being treated, providing pain relief during the procedure. With local anesthesia, you remain awake and conscious throughout the procedure, but you should not feel any pain or discomfort at the access site. This option may be suitable for less complex procedures or for patients who prefer to be awake during the procedure.

Conscious sedation: Conscious sedation involves the administration of medications to help you relax and feel drowsy during the procedure. It is typically administered through an intravenous (IV) line. The medications used for conscious sedation may include benzodiazepines or opioids, which provide relaxation, pain relief, and potential amnesia during the procedure. With conscious sedation, you may be less aware of the procedure and experience minimal discomfort or anxiety. While you may be conscious, you may not remember the details of the procedure. The healthcare team monitors your vital signs and adjusts the medication dosage as needed to maintain your comfort and safety.

General anesthesia: In some cases, particularly for more complex procedures or if the patient has specific medical conditions, general anesthesia may be used. General anesthesia induces a state of complete unconsciousness, and you will be unaware and have no memory of the procedure. This type of anesthesia is typically administered by an anesthesiologist or a certified anesthetist, who closely monitors your vital signs and manages your airway throughout the procedure.

The choice of anesthesia or sedation depends on various factors, including the complexity of the procedure, patient preferences, medical conditions, and the healthcare team's expertise. The healthcare provider will discuss the options with you, explain the associated risks and benefits, and help determine the most appropriate approach for your PTA procedure.

It's important to note that regardless of the type of anesthesia or sedation used, the healthcare team will take all necessary measures to ensure your comfort, monitor your vital signs, and provide pain management during the PTA procedure.

11. Will I need to stay overnight in the hospital after the procedure?

Whether you need to stay overnight in the hospital after a percutaneous transluminal angioplasty (PTA) procedure depends on various factors, including the complexity of the procedure, your overall health, and the healthcare team's assessment of your condition. Here are some considerations:

Same-day discharge: Many PTA procedures are performed on an outpatient basis, meaning you can go home on the same day as the procedure. If your PTA is straightforward, uncomplicated, and there are no significant underlying medical conditions, you may be discharged a few hours after the procedure. However, this decision will be made by your healthcare team based on your specific circumstances.

Observation period: In some cases, the healthcare team may prefer to observe you for a certain period after the PTA procedure. This is to ensure there are no immediate complications and to

monitor your vital signs, access site, and overall recovery. If an observation period is necessary, you may need to stay in the hospital overnight.

Complex procedures or underlying health conditions: If your PTA procedure is more complex or if you have underlying health conditions that require closer monitoring, the healthcare team may recommend an overnight stay in the hospital. This allows for continued observation, pain management, and any necessary interventions or treatments.

Access site considerations: The location of the access site for the PTA procedure can also impact the decision for overnight stay. If the access site is in the groin, there may be a higher risk of bleeding or complications that require monitoring for a longer period.

It's important to discuss with your healthcare team whether an overnight hospital stay is anticipated for your specific PTA procedure. They will consider the procedure complexity, your medical history, and individual factors to make an informed decision about your post-procedure care.

If you are discharged on the same day as the PTA procedure, the healthcare team will provide you with detailed instructions for at-home care, including any restrictions, medication management, and signs of potential complications. Regardless of whether you stay overnight or go home the same day, it's important to follow the post-procedure instructions provided by your healthcare team and attend any scheduled follow-up appointments.

12. How will I be monitored during the procedure?

During a percutaneous transluminal angioplasty (PTA) procedure, you will be closely monitored by a healthcare team to ensure your safety and well-being. Here are some common monitoring methods used during the procedure:

Vital signs monitoring: Throughout the procedure, your vital signs will be continuously monitored. This typically includes measuring your blood pressure, heart rate, and oxygen saturation levels. Monitoring these vital signs helps the healthcare team assess your cardiovascular stability and overall well-being during the procedure.

Electrocardiogram (ECG): An ECG is commonly used to monitor your heart's electrical activity during the procedure. Electrode patches or leads will be placed on your chest, and the ECG will provide continuous information about your heart's rhythm and any potential changes or abnormalities.

Oxygen monitoring: Your blood oxygen levels will be monitored using a pulse oximeter, which is usually attached to your finger or earlobe. This non-invasive device measures the oxygen saturation in your blood, ensuring that you are receiving adequate oxygenation throughout the procedure.

End-tidal CO_2 monitoring: In some cases, end-tidal CO_2 monitoring may be used to measure the carbon dioxide levels in your exhaled breath. This monitoring method helps assess your respiratory function during the procedure.

Pain and comfort assessment: The healthcare team will regularly communicate with you during the procedure to assess your comfort level and any pain or discomfort you may be experiencing. They will adjust pain medications or techniques as needed to ensure your comfort.

Angiography monitoring: If angiography is performed during the procedure to visualize the blood vessels, real-time X-ray images will be continuously monitored to guide the catheter placement and assess the results of the PTA.

The specific monitoring methods used during a PTA procedure may vary based on the facility, the complexity of the procedure, and your individual needs. The healthcare team will closely observe and assess your condition throughout the procedure to detect any changes, complications, or adverse reactions promptly.

It's important to communicate with the healthcare team during the procedure if you experience any significant discomfort, pain, or concerns. They are there to ensure your safety and well-being and will address any issues promptly.

Risks and Complications:

1. What are possible risks and complications of percutaneous transluminal angioplasty (PTA)?

Percutaneous transluminal angioplasty (PTA) is generally considered a safe and effective procedure for treating peripheral arterial disease. However, as with any medical procedure, there are potential risks and complications that can arise. It's important to discuss these risks with your healthcare provider before undergoing PTA. Here are some possible risks and complications:

Access site complications: PTA typically involves accessing the blood vessels through an artery, most commonly in the groin area. There is a risk of complications at the access site, including bleeding, hematoma (collection of blood), infection, or damage to the blood vessels or nerves.

Artery injury or dissection: During the procedure, there is a small risk of injury to the treated artery, which could result in an arterial dissection (tear) or perforation. This may require additional intervention or surgery to repair the damage.

Restenosis: After successful PTA, there is a possibility of restenosis, which is the re-narrowing or recurrence of the arterial blockage over time. This can require additional treatments or procedures to maintain adequate blood flow.

Blood clots: PTA can trigger the formation of blood clots within the treated artery or at the access site. These clots may partially or completely block blood flow, potentially leading to complications such as ischemia (lack of blood supply) or thrombosis (complete blockage).

Allergic reactions: Some patients may have an allergic reaction to the contrast dye used during the procedure. Although rare, this can range from mild skin reactions to severe allergic responses. Inform your healthcare team if you have a known allergy to contrast agents.

Embolization: During PTA, small particles or debris can break loose from the treated artery and travel downstream, causing blockages in smaller blood vessels. This can lead to tissue damage or ischemia in the affected area.

Kidney damage: The contrast dye used during angiography can potentially affect kidney function, particularly in individuals with pre-existing kidney problems. The risk is generally low, but precautions may be taken for high-risk individuals.

Infection: Although uncommon, there is a risk of infection associated with PTA, such as access site infections or systemic infections. Proper sterile techniques and antibiotic prophylaxis are typically used to minimize this risk.

Rare complications: Rare complications of PTA may include allergic reactions to medications, adverse reactions to anesthesia or sedation, cardiac arrhythmias, stroke, or damage to other structures near the treated area.

It's important to note that the overall risk of complications during PTA is generally low. However, the specific risks can vary depending on individual factors, such as the extent of arterial disease, overall health, and other underlying medical conditions.

Your healthcare provider will thoroughly assess your individual situation, discuss the potential risks and benefits of PTA, and take appropriate measures to minimize the risks and optimize the outcomes of the procedure.

2. How safe is PTA for pAD?

Percutaneous transluminal angioplasty (PTA) is generally considered a safe and effective treatment option for peripheral arterial disease (PAD). The safety of PTA depends on various factors, including

the patient's overall health, the extent and severity of PAD, and the expertise of the healthcare team performing the procedure. Here are some points to consider regarding the safety of PTA for PAD:

Low complication rates: PTA is associated with low complication rates. Most patients undergo the procedure without experiencing any major complications.

Minimally invasive approach: PTA is a minimally invasive procedure, performed through a small incision or puncture in the skin. This reduces the risks associated with traditional open surgical procedures.

Local anesthesia: PTA is often performed under local anesthesia, reducing the risks associated with general anesthesia.

Improved techniques and technology: Advances in interventional radiology and endovascular techniques have contributed to the safety and efficacy of PTA. These advancements have allowed for more precise and targeted treatments with better outcomes.

Individualized approach: The healthcare team assesses each patient's specific condition, taking into account factors such as the location and severity of arterial blockages, overall health, and other individual characteristics. This personalized approach helps optimize the safety and effectiveness of the procedure.

Expertise of the healthcare team: The safety of PTA greatly relies on the expertise and experience of the healthcare team performing the procedure. Skilled interventional radiologists, vascular surgeons, or other specialists with training in endovascular techniques can minimize the risks and handle any potential complications effectively.

Potential risks and complications: Although PTA is generally safe, it's important to be aware of potential risks and complications, as discussed in the previous question. These risks, while relatively low, can vary depending on individual factors and the specific circumstances of each case.

It's crucial to have a thorough discussion with your healthcare provider about the potential risks and benefits of PTA for your specific PAD condition. They can provide you with personalized information, address any concerns you may have, and help you make an informed decision about the most suitable treatment approach for your individual situation.

3. Are there any specific factors that increase my risk of complications?

Yes, certain factors can increase the risk of complications associated with percutaneous transluminal angioplasty (PTA) for peripheral arterial disease (PAD). It's important to discuss these factors with

your healthcare provider, as they can help determine the best course of action for your specific situation. Here are some factors that may increase the risk of complications:

Severe PAD: If you have advanced or severe PAD with extensive arterial blockages, the procedure may be technically challenging, and the risk of complications, such as arterial dissection or perforation, may be higher.

Underlying health conditions: Certain medical conditions can increase the risk of complications during PTA. These may include uncontrolled diabetes, kidney disease, severe heart disease, lung disease, or clotting disorders. These conditions can affect the body's ability to heal or tolerate the procedure.

Age: Older age alone does not necessarily increase the risk of complications, but older individuals often have more underlying health conditions that may contribute to a higher risk.

Obesity: Obesity can pose challenges during the procedure, such as difficulties with access site closure and increased risk of bleeding or infection.

Previous interventions or surgeries: If you have had prior interventions or surgeries in the same area, there may be scar tissue or anatomical changes that can increase the complexity of the procedure and the risk of complications.

Smoking: Smoking is a significant risk factor for PAD and can also increase the risk of complications during and after PTA. Smoking affects blood vessel health and healing, making it important to quit smoking before the procedure.

Allergies or sensitivities: If you have known allergies or sensitivities to contrast agents or medications used during the procedure, this can increase the risk of allergic reactions or other adverse events.

Medications: Certain medications, such as blood thinners or antiplatelet drugs, can increase the risk of bleeding or affect the body's response to the procedure. It's important to discuss your medications with your healthcare provider prior to the procedure.

Limited mobility or impaired circulation: If you have limited mobility or impaired circulation in the affected limb, it may affect the procedure's success and increase the risk of complications.

These are just some of the factors that can potentially increase the risk of complications during PTA. Your healthcare provider will evaluate your individual circumstances, consider these factors, and take necessary precautions to minimize the risks associated with the procedure. It's important to have

open and thorough discussions with your healthcare provider to ensure a comprehensive assessment of your risk profile and to make informed decisions about your treatment options.

4. What steps are taken to minimize the risks during the procedure?

During a percutaneous transluminal angioplasty (PTA) procedure, several steps are taken to minimize the risks and ensure the safety of the patient. These steps include:

Pre-procedure assessment: Before the procedure, a comprehensive evaluation is performed to assess the patient's overall health, medical history, and specific condition. This evaluation helps identify any potential risks or contraindications and allows for appropriate planning and preparation.

Expert healthcare team: PTA is typically performed by a skilled and experienced healthcare team, including interventional radiologists, vascular surgeons, or other specialists trained in endovascular procedures. Their expertise and familiarity with the procedure help minimize risks and handle any complications effectively.

Patient preparation: The patient may be required to undergo certain pre-procedure preparations, such as fasting for a specific period, discontinuing certain medications, or adjusting the dosage of anticoagulants or antiplatelet drugs. These steps help minimize the risk of complications during and after the procedure.

Patient monitoring: Throughout the procedure, the patient's vital signs, including blood pressure, heart rate, and oxygen saturation, are continuously monitored to ensure stability and detect any changes or complications promptly. Electrocardiogram (ECG) monitoring may also be used to monitor heart rhythm.

Local anesthesia or sedation: PTA is often performed under local anesthesia, which numbs the area being treated. In some cases, conscious sedation may be used to help the patient relax and remain comfortable during the procedure. The type and level of anesthesia or sedation used depend on the individual patient and the complexity of the procedure.

Sterile techniques: The procedure is performed using sterile techniques to minimize the risk of infection. The healthcare team follows strict protocols for maintaining a sterile environment, including wearing sterile gloves, gowns, and masks, and using sterile equipment and drapes.

Fluoroscopy and imaging guidance: PTA is performed using real-time X-ray imaging guidance, known as fluoroscopy. This allows the healthcare team to visualize the blood vessels and guide the catheter and other instruments with precision. The use of imaging guidance helps minimize the risk of accidental injury or damage to surrounding structures.

Minimizing radiation exposure: Measures are taken to minimize radiation exposure to both the patient and the healthcare team during the fluoroscopy-guided procedure. Lead aprons and shields are used to protect the patient and healthcare providers from unnecessary radiation exposure.

Medication management: Medications may be administered during the procedure to prevent blood clots, manage pain, or address any other specific needs. The type and dosage of medications are carefully determined based on the patient's condition and individual requirements.

Post-procedure care and monitoring: After the procedure, the patient is monitored for a period to ensure stability and detect any potential complications. Instructions are provided for post-procedure care, including wound care, activity restrictions, medication management, and follow-up appointments.

These steps, among others, are implemented to minimize risks and optimize the safety of PTA procedures. The specific measures taken may vary depending on the patient's individual circumstances, the complexity of the procedure, and the healthcare facility's protocols. Open communication with the healthcare team is essential to address any concerns and ensure a well-coordinated and safe procedure.

5. Can I die during the procedure?

While percutaneous transluminal angioplasty (PTA) is generally considered a safe procedure, it's important to understand that there is always a small risk of complications during any medical procedure, including PTA. While rare, severe complications can potentially lead to life-threatening situations. These risks can vary depending on individual factors, the extent and severity of peripheral arterial disease (PAD), and other medical conditions. It's essential to have an open and thorough discussion with your healthcare provider regarding the potential risks and benefits specific to your situation.

It's worth noting that the healthcare team performing the procedure takes numerous precautions to minimize risks and ensure patient safety. They are trained to manage any complications that may arise during the procedure promptly. Furthermore, PTA is typically performed in a controlled and monitored environment with access to emergency equipment and personnel.

In the unlikely event of a severe complication, such as an arterial dissection, perforation, or a major allergic reaction, there is a possibility of life-threatening consequences. However, it's important to remember that the overall risk of such complications is relatively low.

Your healthcare provider will evaluate your individual circumstances, assess your risk profile, and discuss any specific concerns or potential risks associated with the procedure. They will also inform you of any emergency protocols in place during the procedure to address any complications swiftly and effectively.

Remember to ask your healthcare provider any questions or concerns you may have about the risks and potential complications associated with PTA. They are best equipped to provide you with personalized information and help you make an informed decision about your treatment.

6. Are there any complications that can occur after the procedure?

Yes, there are potential complications that can occur after a percutaneous transluminal angioplasty (PTA) procedure. While most patients recover well without experiencing significant complications, it's important to be aware of potential risks and promptly report any concerning symptoms to your healthcare provider. Here are some possible complications that can occur after PTA:

Restenosis: Restenosis refers to the recurrence or re-narrowing of the treated artery after PTA. Although PTA can effectively open up blocked arteries, there is a risk that the artery may become narrowed again over time. The rate of restenosis can vary depending on various factors, such as the location of the treated artery and the severity of the underlying arterial disease.

Thrombosis: Blood clot formation within the treated artery, known as thrombosis, can occur after PTA. This can partially or completely block the blood flow, potentially leading to symptoms such as pain, ischemia, or tissue damage. Antiplatelet or anticoagulant medications are often prescribed after the procedure to reduce the risk of clot formation.

Hematoma or bleeding: A hematoma, which is a collection of blood, can form at the access site or in the surrounding tissues. In some cases, bleeding from the access site may occur. Prompt medical attention is necessary if there is significant bleeding or if the hematoma enlarges, causing pain or pressure.

Infection: Although uncommon, there is a risk of infection at the access site or in the treated artery. Signs of infection may include increased pain, redness, swelling, or drainage from the site. If you develop any signs of infection, it's important to notify your healthcare provider promptly.

Arterial dissection or injury: In rare cases, the PTA procedure itself can cause injury to the treated artery, leading to arterial dissection (tear) or perforation. These complications may require further intervention or surgery to address the damage.

Allergic reactions: Some individuals may have an allergic reaction to the contrast dye used during the procedure. While uncommon, allergic reactions can range from mild to severe and may require immediate medical attention.

Kidney problems: The contrast dye used during angiography can potentially affect kidney function, particularly in individuals with pre-existing kidney problems. Your healthcare provider will take precautions and monitor your kidney function if you are at risk.

Other rare complications: Rare complications of PTA may include blood vessel rupture, embolization of particles or debris to other blood vessels, cardiac arrhythmias, stroke, or damage to nearby structures.

It's important to follow post-procedure instructions provided by your healthcare provider, including taking prescribed medications, monitoring the access site, and attending follow-up appointments. If you experience any concerning symptoms, such as worsening pain, bleeding, swelling, or signs of infection, it's crucial to seek medical attention promptly.

Discussing potential complications and post-procedure care with your healthcare provider will help you understand what to expect and how to manage any possible complications effectively.

7. How are intraprocedural complications treated?

Intraprocedural complications during a percutaneous transluminal angioplasty (PTA) procedure for peripheral arterial disease (PAD) are typically managed promptly by the healthcare team performing the procedure. The specific treatment approach depends on the nature and severity of the complication. Here are some common intraprocedural complications and their potential treatments:

Arterial dissection or perforation: If an arterial dissection (tear) or perforation occurs during the procedure, the healthcare team will assess the severity and location of the injury. Treatment options may include:

Additional angioplasty: In some cases, the dissection or perforation can be managed by further angioplasty or stenting to repair the artery and restore blood flow.

Balloon tamponade: In situations where the bleeding is significant, a balloon catheter may be temporarily inflated to apply pressure against the bleeding vessel, helping to stop the bleeding and stabilize the situation.

Covered stent or graft placement: If the dissection or perforation is severe, a covered stent or graft may be placed to seal the damaged artery and provide a conduit for blood flow.

Conversion to surgery: In rare instances where endovascular techniques are insufficient to address the complication, the procedure may be halted, and the patient may be taken to the operating room for open surgical repair.

Embolization: If particles or debris are dislodged during the procedure and pose a risk of embolizing (traveling) to other blood vessels, the healthcare team will take steps to retrieve or remove the emboli. This may involve the use of specialized retrieval devices or techniques to capture and remove the emboli from the bloodstream.

Allergic reactions: If an allergic reaction to contrast dye or other medications occurs, immediate treatment is necessary. The healthcare team is prepared to manage allergic reactions with medications, such as antihistamines or corticosteroids, and in severe cases, with emergency measures like administering epinephrine and providing respiratory support.

Cardiac arrhythmias or hemodynamic instability: In the event of cardiac arrhythmias or significant changes in blood pressure or heart rate, the healthcare team will closely monitor the patient's condition and take appropriate measures to stabilize the heart rhythm or manage hemodynamic instability. This may involve medications, electrical cardioversion, or other interventions to restore normal cardiac function.

It's important to note that the management of intraprocedure complications requires the expertise and experience of the healthcare team performing the procedure. They are trained to identify and respond to complications swiftly to ensure patient safety. Your healthcare team will take all necessary precautions to minimize the risk of complications, but it's important to be aware that complications can occur despite these efforts.

During the informed consent process, your healthcare provider will discuss potential complications with you and explain the steps they will take to manage them. It's crucial to have open communication with your healthcare provider and trust in their expertise to address any complications that may arise during the procedure.

8. Will there be blood loss during the procedure?

During a percutaneous transluminal angioplasty (PTA) procedure, there may be minimal blood loss, but it is generally limited. The procedure is designed to minimize bleeding and blood loss as much as possible. Here are some factors that contribute to the limited blood loss during PTA:

Local anesthesia: Before the procedure, the access site where the catheter is inserted is typically numbed with a local anesthetic. This helps minimize discomfort during the procedure and reduces the likelihood of significant bleeding from the access site.

Small incision or puncture: PTA is a minimally invasive procedure that involves making a small incision or puncture at the access site, usually in the groin or arm. The incision or puncture site is typically small, which helps minimize blood loss.

Hemostatic techniques: During and after the procedure, various hemostatic techniques may be employed to control bleeding and promote clot formation. These techniques can include the application of pressure at the access site, the use of closure devices, or the administration of medications that promote blood clotting.

Vascular closure devices: In some cases, vascular closure devices may be used to seal the access site and facilitate faster hemostasis. These devices help minimize the time required for manual compression and reduce the risk of bleeding.

Close monitoring: Throughout the procedure, the healthcare team closely monitors the patient's vital signs, including blood pressure and heart rate. Any changes indicating significant bleeding would be promptly addressed.

It's important to note that while blood loss during PTA is generally limited, it can vary depending on individual factors, the complexity of the procedure, and the patient's underlying medical condition. In rare cases, complications such as arterial dissection or perforation can result in more significant bleeding.

Your healthcare provider and the healthcare team performing the procedure will take all necessary precautions to minimize blood loss and manage any potential bleeding. They are experienced in ensuring patient safety and will closely monitor you during the procedure to address any bleeding that may occur promptly.

If you have concerns about blood loss or bleeding during the procedure, it's important to discuss them with your healthcare provider beforehand. They can provide you with personalized information based on your specific situation and address any questions or concerns you may have.

9. What structures can be injured during the procedure?

During a percutaneous transluminal angioplasty (PTA) procedure, there is a potential risk of injury to certain structures near the treated area. The specific structures that can be at risk of injury during PTA depend on the location of the arterial blockage and the approach used for accessing the blocked artery. Here are some structures that can be at risk of injury:

Arteries and blood vessels: While PTA aims to treat arterial blockages, there is a small risk of injuring the treated artery or adjacent blood vessels during the procedure. This can include arterial dissection (tear) or perforation, which may require additional interventions to address.

Nerves: In certain cases, nerves near the treatment site may be at risk of injury. This can potentially lead to nerve damage and result in symptoms such as numbness, tingling, or pain in the affected area. However, nerve injuries during PTA are relatively rare.

Muscles and soft tissues: During the insertion of the catheter or other interventional devices, there is a possibility of inadvertently injuring muscles or other soft tissues near the access site or within the treated area. This can result in localized pain, swelling, or bruising.

Organs or structures adjacent to the treated area: Depending on the location of the arterial blockage, there may be organs or structures adjacent to the treated artery that can be at risk of injury. For example, if the procedure involves arteries in the abdomen or pelvis, nearby organs such as the intestines, bladder, or reproductive organs may be at risk.

It's important to note that while the risk of injury to these structures exists, healthcare professionals performing the procedure are trained to minimize such risks and take precautions to avoid complications. They use imaging guidance, such as fluoroscopy or angiography, to visualize the blood vessels and guide the placement of the catheter or other devices.

Before the procedure, your healthcare provider will review your medical history, perform a physical examination, and evaluate imaging studies to assess the potential risks and benefits specific to your situation. They will take necessary precautions to minimize the risk of injury and carefully navigate the treatment area during the procedure.

If you have concerns about potential injury to specific structures during PTA, it's important to discuss them with your healthcare provider. They can provide you with personalized information and address any questions or concerns you may have regarding the procedure.

Postinterventional aftercare:

1. What do I need to consider after the procedure?

After undergoing percutaneous transluminal angioplasty (PTA) for peripheral arterial disease (PAD), there are several important considerations to keep in mind during the recovery phase. Here are some key points to consider:

Follow post-procedure instructions: It is crucial to carefully follow the instructions provided by your healthcare provider after the surgery. These instructions may include specific guidelines for wound care, medication management, physical activity, and follow-up appointments. Adhering to these instructions promotes proper healing and reduces the risk of complications.

Monitor the access site: Keep a close eye on the access site where the catheter was inserted. Look for any signs of infection, such as redness, swelling, increased pain, or drainage. Follow the recommended care instructions for the access site, such as keeping it clean and dry, as advised by your healthcare provider.

Take prescribed medications: Your healthcare provider may prescribe medications to help prevent complications or manage underlying conditions. It is essential to take these medications as prescribed and follow any specific instructions regarding their administration, dosage, and duration.

Gradually resume physical activity: Your healthcare provider will provide guidance on when and how to resume physical activities after the procedure. Initially, you may need to limit strenuous activities and gradually increase your activity level based on your provider's recommendations. Regular physical activity can help improve circulation and overall cardiovascular health.

Maintain a healthy lifestyle: Adopting a healthy lifestyle can contribute to the success of the PTA procedure and overall well-being. This includes maintaining a balanced diet, managing weight, avoiding tobacco use, and controlling underlying medical conditions such as diabetes and hypertension. Your healthcare provider can provide specific guidance on lifestyle modifications tailored to your needs.

Attend follow-up appointments: It is important to attend all scheduled follow-up appointments with your healthcare provider. These appointments allow your provider to monitor your progress, assess the effectiveness of the PTA procedure, and make any necessary adjustments to your treatment plan. They can also address any concerns or questions you may have.

Be vigilant for recurrent symptoms: While PTA can effectively improve blood flow and relieve symptoms associated with PAD, there is a possibility of restenosis (re-narrowing) over time. Be aware of any recurrent symptoms, such as leg pain or discomfort, that may indicate a need for further evaluation or treatment. Report any changes or concerns to your healthcare provider promptly.

Remember to communicate openly with your healthcare provider and seek their guidance throughout your recovery period. They can provide personalized advice and address any specific concerns or questions you may have, ensuring a smooth recovery process.

2. Will I experience pain after the surgery?

After percutaneous transluminal angioplasty (PTA) for peripheral arterial disease (PAD), it is common to experience some degree of discomfort or pain. However, the severity and duration of pain can vary among individuals. Here are some points to consider regarding post-surgery pain:

Access site discomfort: You may experience some pain or tenderness at the access site where the catheter was inserted. This is typically located in the groin or arm, depending on the approach used. The level of discomfort can vary, ranging from mild tenderness to more noticeable pain. This discomfort usually improves over time and can be managed with over-the-counter pain relievers as recommended by your healthcare provider.

Leg pain relief: One of the primary goals of PTA is to improve blood flow and alleviate leg pain associated with PAD. In many cases, patients experience significant relief from leg pain shortly after the procedure. However, it's important to note that some individuals may continue to experience residual or intermittent discomfort, particularly if there are other contributing factors to their leg pain, such as nerve compression or underlying musculoskeletal issues.

Restenosis-related symptoms: While PTA can provide initial relief, there is a risk of restenosis (re-narrowing) over time. If restenosis occurs, it may lead to recurrent leg pain or other symptoms associated with PAD. If you experience a return of symptoms or new discomfort in your leg, it's important to inform your healthcare provider promptly for further evaluation.

Pain management: Your healthcare provider will provide specific instructions on pain management after the procedure. This may include recommendations for over-the-counter pain relievers, such as acetaminophen or nonsteroidal anti-inflammatory drugs (NSAIDs), to help manage any discomfort or pain. It's important to follow the recommended dosage and duration of medication use as directed by your healthcare provider.

Communicate with your healthcare provider: If you experience severe or persistent pain that is not alleviated by over-the-counter pain relievers, or if you have any concerns about your post-surgery pain, it is crucial to communicate with your healthcare provider. They can assess your individual situation, provide appropriate guidance, and consider alternative pain management strategies if necessary.

Every individual's pain experience can differ, and the extent of pain after PTA can be influenced by various factors, including the complexity of the procedure, the extent of arterial blockages, and individual pain tolerance. Your healthcare provider will provide you with personalized instructions and support to manage post-surgery pain effectively.

3. Can I take pain medication after the surgery?

Yes, you can take pain medication after undergoing percutaneous transluminal angioplasty (PTA) for peripheral arterial disease (PAD). Pain medication is often prescribed or recommended to help manage post-surgery discomfort or pain. Here are some important considerations regarding pain medication after the surgery:

Follow healthcare provider's instructions: It is essential to follow your healthcare provider's instructions regarding pain medication after PTA. They will provide specific recommendations based on your individual needs, the severity of the pain, and any other factors that may influence your medication use.

Over-the-counter pain relievers: For mild to moderate pain, your healthcare provider may suggest over-the-counter pain relievers, such as acetaminophen (e.g., Tylenol) or nonsteroidal anti-inflammatory drugs (NSAIDs) like ibuprofen (e.g., Advil, Motrin) or naproxen (e.g., Aleve). These medications can help alleviate post-surgery discomfort and reduce inflammation. However, it's important to check with your healthcare provider before taking any medications to ensure they are appropriate for you and do not interfere with any other medications you may be taking or medical conditions you may have.

Prescription pain medication: In some cases, your healthcare provider may prescribe stronger pain medication to manage post-surgery pain if over-the-counter options are not sufficient. Prescription

pain medication may include opioids or other analgesics. If prescribed, it's crucial to follow the prescribed dosage and duration, as well as any specific instructions or precautions provided by your healthcare provider. Opioids, in particular, should be used cautiously and only as directed due to their potential for dependence and side effects.

Pain management plan: Your healthcare provider will work with you to develop an individualized pain management plan based on your needs and preferences. They will consider factors such as the severity of your pain, your medical history, and any potential interactions with other medications you may be taking. It's important to communicate openly with your healthcare provider about your pain levels and any concerns or questions you may have about pain medication.

Other pain management techniques: Alongside pain medication, your healthcare provider may recommend other pain management techniques to help alleviate discomfort after PTA. These techniques may include applying ice or heat to the affected area, practicing relaxation techniques, engaging in gentle exercise, or using non-medication pain management strategies such as physical therapy or acupuncture.

Remember, it is important to consult your healthcare provider regarding pain medication options after PTA. They will provide personalized recommendations based on your specific circumstances and help you manage post-surgery pain effectively while considering your overall well-being.

4. What kind of care should I provide for the puncture site or incision?

Providing proper care for the puncture site or incision after percutaneous transluminal angioplasty (PTA) is important for promoting healing, preventing infection, and minimizing complications. Here are some general guidelines for caring for the puncture site or incision:

Follow healthcare provider's instructions: Your healthcare provider will provide specific instructions on how to care for the puncture site or incision. It is important to follow their guidance closely and ask any questions you may have. Instructions may vary depending on the specific circumstances of your procedure and the preferences of your healthcare provider.

Keep the area clean and dry: In the initial stages of recovery, it is important to keep the puncture site or incision clean and dry to reduce the risk of infection. Avoid submerging the area in water, such as taking baths or swimming, until your healthcare provider gives you the green light. Showering is generally allowed, but be cautious to avoid direct water pressure on the site. Pat the area dry gently after washing.

Monitor for signs of infection: Watch for signs of infection at the puncture site or incision, including increased redness, swelling, warmth, discharge, or worsening pain. If you notice any of these signs, or if you develop a fever, notify your healthcare provider immediately, as it may indicate an infection that requires medical attention.

Dressing changes, if applicable: Depending on the specific instructions from your healthcare provider, you may need to change the dressing over the puncture site or incision. Follow the recommended schedule and technique for dressing changes, if applicable. Use sterile supplies and clean hands when performing dressing changes, or seek professional assistance if needed.

Avoid scratching or picking: It is important to refrain from scratching or picking at the puncture site or incision, as it can disrupt the healing process and increase the risk of infection. If you experience itching, inform your healthcare provider, as they may recommend appropriate remedies or medications to alleviate the discomfort.

Watch for bleeding: While minimal bleeding from the puncture site or incision is expected initially, it should gradually decrease over time. However, if you notice persistent or excessive bleeding, apply firm pressure to the area and contact your healthcare provider for further guidance.

Follow-up appointments: Attend all scheduled follow-up appointments with your healthcare provider. They will assess the healing progress of the puncture site or incision, remove any sutures or staples if necessary, and address any concerns or questions you may have.

Remember to consult your healthcare provider for specific care instructions tailored to your situation. They will provide you with personalized advice based on the type of procedure performed, the location of the puncture site or incision, and your individual needs.

5. Are there any specific restrictions or limitations on activities following PTA?

Following percutaneous transluminal angioplasty (PTA) for peripheral arterial disease (PAD), there may be some restrictions or limitations on activities during the recovery period. These restrictions are typically temporary and aimed at promoting proper healing and reducing the risk of complications. Here are some general guidelines regarding activity restrictions after PTA:

Rest and recovery: It is important to allow your body time to rest and recover after the procedure. During the immediate post-surgery period, you may be advised to limit physical activity and avoid strenuous movements. This helps to minimize stress on the treated blood vessel and allows the puncture site or incision to heal.

Avoid heavy lifting or strenuous activities: In the initial days or weeks following PTA, your healthcare provider may recommend avoiding heavy lifting or engaging in strenuous activities. These activities can increase blood pressure and strain the treated blood vessel, potentially compromising the healing process. Your healthcare provider will provide specific instructions on the weight limits and duration of activity restrictions based on your individual case.

Gradual resumption of physical activity: Your healthcare provider will guide you on gradually resuming physical activities. Initially, you may be encouraged to engage in light activities such as walking, as tolerated. Over time, you will be able to gradually increase your activity level based on your healthcare provider's recommendations. Regular physical activity is beneficial for cardiovascular health and can help improve blood flow and overall well-being.

Avoid prolonged sitting or standing: It is generally advisable to avoid prolonged periods of sitting or standing in the immediate post-surgery period. This is to minimize stress on the treated blood vessel and reduce the risk of swelling or discomfort. If you have a sedentary job, your healthcare provider may recommend taking breaks and periodically moving or stretching to promote circulation.

Follow specific instructions: Your healthcare provider will provide personalized instructions based on the specifics of your procedure and your individual condition. It is important to follow these instructions carefully. They may include guidelines on when you can resume driving, return to work, participate in sports or recreational activities, and engage in sexual activity. Your healthcare provider's guidance will take into account factors such as the location and complexity of the treated blood vessel and your overall health.

Consult your healthcare provider: If you have any questions or concerns about specific activities or restrictions after PTA, it is important to consult your healthcare provider. They can provide personalized advice based on your situation and help you navigate the recovery period safely and effectively.

Remember, the specific activity restrictions and limitations may vary depending on the details of your procedure and your individual circumstances. It is crucial to follow your healthcare provider's guidance to ensure proper healing and optimal outcomes.

6. How long do I need to remain lying down after PTA?

The duration of lying down or bed rest following percutaneous transluminal angioplasty (PTA) for peripheral arterial disease (PAD) can vary based on individual factors and the specific details of the procedure. Here are some general considerations regarding the duration of lying down after surgery:

Immediate post-surgery period: In the immediate post-surgery period, you will typically be required to remain lying down for a few hours or as advised by your healthcare provider. This allows time for the effects of sedation or anesthesia to wear off, and for initial monitoring and recovery. During this time, you may be in a recovery area or a hospital room where healthcare professionals can closely observe your vital signs and monitor for any immediate complications.

Puncture site or incision management: The duration of lying down may be influenced by the type of access site used for the procedure. If the PTA was performed through a groin artery (femoral artery), your healthcare provider may recommend lying down for a longer period to minimize the risk of

bleeding or hematoma formation at the puncture site. On the other hand, if a different access site (such as the radial artery in the wrist) was used, the lying down requirement may be shorter.

Individual factors: The specific duration of lying down after PTA can vary based on individual factors such as your overall health, the complexity of the procedure, the presence of other medical conditions, and the extent of arterial blockages. Your healthcare provider will consider these factors when determining the appropriate duration of bed rest for you.

Mobility and gradual activity: After the initial period of lying down, your healthcare provider will guide you on gradually increasing your mobility and activity level. This may involve sitting up, standing, and eventually walking with assistance, as tolerated. The timeline for transitioning from lying down to sitting and standing will depend on your specific condition and the guidance of your healthcare provider.

It is important to note that the duration of lying down is typically relatively short, and you will be encouraged to resume mobility and gentle activity as soon as it is deemed safe by your healthcare provider. Early mobilization helps prevent complications such as blood clots and promotes overall recovery.

Your healthcare provider will provide specific instructions regarding the duration of bed rest and any subsequent activity limitations based on your individual situation. It is crucial to follow their guidance closely to ensure a smooth recovery and optimal outcomes.

7. What signs or symptoms should I watch for that may indicate a problem or complication?

After undergoing percutaneous transluminal angioplasty (PTA) for peripheral arterial disease (PAD), it is important to be aware of potential signs or symptoms that may indicate a problem or complication. While complications are relatively rare, recognizing early warning signs allows for prompt medical attention. Here are some signs and symptoms to watch for:

Excessive bleeding or hematoma: Monitor the puncture site or incision for excessive bleeding that doesn't subside with gentle pressure or the development of a large bruise (hematoma). If you notice persistent bleeding or an expanding hematoma, contact your healthcare provider.

Swelling or signs of infection: Pay attention to any significant swelling, redness, warmth, or increasing pain around the puncture site or incision. These could indicate an infection or inflammation. If you observe these signs, inform your healthcare provider.

Changes in skin color or temperature: Observe the color and temperature of your affected limb. If you notice a pale or bluish discoloration, or if the limb feels unusually cold to the touch, it could be a sign of reduced blood flow. Notify your healthcare provider immediately.

Numbness, tingling, or weakness: Any new or persistent sensations of numbness, tingling, or weakness in the affected limb should be reported to your healthcare provider. These symptoms may indicate nerve involvement or compromised blood flow.

Severe pain: While some discomfort or mild pain is expected after the procedure, severe or worsening pain that is not relieved with rest, elevation, or pain medication may require medical attention. Contact your healthcare provider to discuss your symptoms.

Changes in pulse or blood pressure: If you notice a significant change in your pulse (absence or weakening) or experience sudden high or low blood pressure, seek immediate medical attention.

Fever or chills: If you develop a fever above 100.4°F (38°C) or experience chills, it could be a sign of infection. Notify your healthcare provider promptly.

Shortness of breath or chest pain: If you experience sudden or severe shortness of breath, chest pain, or any other symptoms suggestive of a heart attack or pulmonary embolism, call emergency services (such as 911 in the United States) immediately.

It is important to remember that the above symptoms are general indications and may not necessarily be related to complications from PTA. However, it is essential to promptly communicate any concerns or new symptoms to your healthcare provider. They can evaluate your specific situation, provide appropriate advice, and take the necessary steps to address any potential complications.

In case of any doubt or uncertainty, do not hesitate to seek medical attention or contact your healthcare provider for guidance.

8. Will I have a scar?

The presence of a scar after percutaneous transluminal angioplasty (PTA) for peripheral arterial disease (PAD) depends on several factors, including the access site used for the procedure and individual healing characteristics. Here are some considerations regarding scarring after PTA:

Access site and incision type: The choice of access site for PTA can vary. Common access sites include the groin (femoral artery), wrist (radial artery), or other peripheral arteries. The size and location of the incision or puncture site will influence the extent of scarring. In some cases, a small puncture or incision may be made, resulting in a minimal scar or even no visible scar over time.

Healing process: The healing process varies from person to person. Factors such as individual skin characteristics, wound care, and overall health can affect the appearance of scars. It is normal for scars to be red, raised, or noticeable in the initial stages of healing. However, over time, scars typically mature and fade, becoming flatter, lighter in color, and less noticeable.

Scar care: Following PTA, your healthcare provider may provide specific instructions on scar care to promote optimal healing. This may include keeping the incision or puncture site clean and dry, avoiding direct sunlight or using sunscreen to protect the area from UV exposure, and gently massaging the scar as it heals. Proper scar care can help minimize the appearance of scars.

Individual variations: Scarring can vary greatly among individuals. Some people may have a predisposition to develop more prominent scars, while others may have a tendency for less visible scarring. The final appearance of the scar will depend on multiple factors, including genetics, skin type, and the individual's healing response.

It is important to note that while scarring is a possibility after PTA, the goal of the procedure is to improve blood flow and alleviate symptoms associated with PAD. The cosmetic aspect of scarring is often secondary to the overall success of the treatment.

If you have concerns about scarring or would like specific information regarding the expected appearance of scars after PTA, it is best to discuss these questions with your healthcare provider. They can assess your specific situation and provide guidance based on your individual circumstances.

9. How long does it take to feel an improvement of my pAD after PTA?

The timeframe for feeling an improvement in peripheral arterial disease (PAD) symptoms following percutaneous transluminal angioplasty (PTA) can vary from person to person. While some individuals may experience immediate relief or noticeable improvement shortly after the procedure, others may require more time for the benefits of PTA to become apparent. Here are some factors to consider regarding the timeline for improvement:

Immediate relief: In certain cases, individuals may experience immediate relief of symptoms during or shortly after the PTA procedure. This can occur when a severely narrowed or blocked artery is successfully treated, leading to improved blood flow and alleviation of symptoms such as pain, cramping, or discomfort.

Individual response: Each person's response to PTA can be unique. Some individuals may notice significant improvement in their PAD symptoms within a few days or weeks following the procedure, while others may require more time to experience noticeable changes. The rate of improvement can depend on factors such as the severity of PAD, the extent of arterial blockages, and the overall health and healing capacity of the individual.

Healing and adaptation: After PTA, the treated blood vessel undergoes a healing process, and the surrounding tissues adapt to the improved blood flow. This healing and adaptation process can take time. Over a period of weeks or months, as the blood vessel heals and the body adjusts to the improved circulation, individuals may gradually experience a reduction in symptoms and an improvement in their PAD-related quality of life.

Rehabilitation and lifestyle modifications: Along with PTA, lifestyle modifications and rehabilitation may be recommended to optimize the benefits of the procedure and improve overall PAD management. These may include activities such as regular exercise, smoking cessation, dietary changes, and medication management. Engaging in these supportive measures can contribute to long-term improvement and symptom control.

It is important to have realistic expectations regarding the timeline for improvement after PTA. While some individuals may experience immediate relief, others may require a longer period for noticeable changes to occur. Regular follow-up appointments with your healthcare provider are crucial for monitoring your progress and assessing the effectiveness of the procedure. If you have specific concerns or questions about the expected timeline for improvement in your individual case, it is best to discuss them with your healthcare provider, as they can provide personalized guidance based on your unique circumstances.

10. How long does the improvement last?

The duration of improvement following percutaneous transluminal angioplasty (PTA) for peripheral arterial disease (PAD) can vary from person to person. While PTA can provide significant benefits in improving blood flow and relieving PAD symptoms, the long-term outcome depends on various factors. Here are some considerations regarding the duration of improvement:

Individual factors: The duration of improvement can be influenced by individual factors such as the extent and severity of PAD, overall health, lifestyle choices, and adherence to recommended medical therapies. Individuals who actively manage their risk factors, adopt a healthy lifestyle, and follow medical advice may experience longer-lasting benefits from PTA.

Arterial restenosis: One factor that can affect the duration of improvement is the possibility of arterial restenosis. Restenosis refers to the re-narrowing or re-blocking of the treated artery following PTA. While PTA can initially open up the artery and improve blood flow, there is a risk of restenosis over time. The rate of restenosis can vary, but it is estimated to occur in a significant proportion of patients within one to two years after the procedure.

Lifestyle modifications and medical management: To maintain the benefits of PTA and prolong the duration of improvement, it is essential to adopt a healthy lifestyle and adhere to recommended medical therapies. This may include regular exercise, smoking cessation, managing blood pressure and cholesterol levels, and taking prescribed medications as directed. These measures help reduce the progression of PAD and minimize the risk of restenosis.

Regular follow-up care: Regular follow-up appointments with your healthcare provider are crucial to monitor the status of your PAD and assess the effectiveness of the PTA procedure. Through periodic check-ups and imaging studies, your healthcare provider can identify any signs of restenosis or other issues and recommend appropriate interventions to maintain or enhance the benefits of PTA.

It is important to note that while PTA can provide significant improvement in PAD symptoms and quality of life, the disease itself is chronic and progressive. Ongoing management and regular follow-up care are essential to optimize long-term outcomes and minimize the risk of complications or disease progression.

Ultimately, the duration of improvement following PTA will depend on a combination of individual factors, adherence to recommended lifestyle changes and medical therapies, and ongoing monitoring and management of PAD. Your healthcare provider can provide personalized guidance and discuss your specific situation, as they are best equipped to evaluate the duration of improvement based on your unique circumstances.

11. Do I need to take any new medications after PTA?

Whether you need to take any new medications after percutaneous transluminal angioplasty (PTA) for peripheral arterial disease (PAD) depends on your individual case and the recommendations of your healthcare provider. Here are some common medications that may be prescribed after PTA:

Antiplatelet medications: Antiplatelet drugs, such as aspirin or clopidogrel, are often prescribed after PTA to reduce the risk of blood clots forming in the treated blood vessels. These medications help prevent reblockage of the artery and maintain blood flow. The specific type and duration of antiplatelet therapy will be determined by your healthcare provider based on your medical history and individual risk factors.

Statins: Statin medications are commonly prescribed to manage cholesterol levels and reduce the risk of atherosclerosis progression. They can help lower LDL (bad) cholesterol and stabilize plaques in the arteries. If you are not already taking a statin, your healthcare provider may consider starting or adjusting your statin therapy after PTA.

Blood pressure medications: If you have high blood pressure (hypertension), your healthcare provider may prescribe or adjust medications to help control your blood pressure. Maintaining optimal blood pressure levels is important for reducing the risk of further arterial damage and promoting overall cardiovascular health.

Medications for symptom management: Depending on your specific symptoms and medical history, your healthcare provider may prescribe medications to manage pain, improve circulation, or alleviate other symptoms associated with PAD. These may include medications such as vasodilators, analgesics, or medications that improve walking distance or quality of life.

It's important to have a discussion with your healthcare provider about your current medications and any new medications that may be prescribed after PTA. They will consider your individual needs, medical history, and specific PAD condition to determine the most appropriate medication regimen for you. They can explain the benefits, potential side effects, and proper usage of any prescribed medications, as well as address any concerns or questions you may have.

Remember to follow your healthcare provider's instructions regarding medication usage, dosage, and frequency. It is also important to inform them about any other medications, supplements, or over-the-counter drugs you are taking to avoid any potential interactions or complications.

12. Can I undergo an MRI with a stent?

In general, most stents used in peripheral arterial disease (PAD) are considered safe for magnetic resonance imaging (MRI) procedures. However, there are some important considerations and precautions to keep in mind:

Stent type: Different types of stents may have different levels of compatibility with MRI. The majority of modern stents, including those used in PAD, are typically made of materials that are MRI-compatible. These include self-expanding nitinol stents, which are commonly used in PAD treatment.

Stent size and location: The size and location of the stent may influence the decision to undergo an MRI. Large stents or stents located in sensitive areas may have higher risks associated with MRI due to potential heating or movement of the stent. The specific details of your stent, including its size, location, and type, should be discussed with your healthcare provider and the MRI facility to assess the safety and feasibility of undergoing an MRI.

MRI machine and settings: The MRI facility must have appropriate equipment and protocols in place to safely perform an MRI in patients with stents. The specific MRI machine, its field strength, and imaging parameters need to be considered to ensure compatibility with the stent and minimize any potential risks.

Consultation with specialists: It is essential to consult with both your vascular specialist who placed the stent and the radiologist overseeing the MRI procedure. They can review your medical history, assess the type and location of the stent, and make an informed decision regarding the safety and feasibility of undergoing an MRI.

Alternative imaging techniques: If MRI is contraindicated or poses risks due to the presence of a stent, alternative imaging techniques such as computed tomography (CT) angiography or ultrasound may be considered to gather the necessary diagnostic information.

It is important to communicate your medical history, including the presence of a stent, to the MRI facility and the healthcare professionals involved in your care. They will evaluate your individual circumstances and determine the most appropriate imaging modality and safety measures.

Remember, the decision to undergo an MRI with a stent should be made collectively by your healthcare team, considering the benefits of the imaging procedure and the potential risks associated with the stent.

13. Will I need any follow-up appointments?

Yes, after undergoing percutaneous transluminal angioplasty (PTA) for peripheral arterial disease (PAD), you will typically need follow-up appointments with your healthcare provider. These appointments are important for monitoring your progress, assessing the effectiveness of the procedure, and managing your ongoing care. Here are some key points regarding follow-up appointments after PTA:

Timing and frequency: The timing and frequency of follow-up appointments can vary depending on several factors, including the specific details of your procedure, the severity of your PAD, and your overall health. In general, you can expect to have a follow-up appointment within a few weeks after the PTA procedure. Further follow-up appointments may be scheduled at regular intervals over the subsequent months and years, as determined by your healthcare provider.

Evaluation of symptoms and progress: During follow-up appointments, your healthcare provider will assess your symptoms and monitor your progress. They will ask about any changes in your symptoms, such as pain, discomfort, or difficulty walking. They may also perform physical examinations and imaging tests, such as ultrasound or angiography, to evaluate the status of your treated arteries and assess the effectiveness of the PTA procedure.

Medication management: Follow-up appointments provide an opportunity for your healthcare provider to review and adjust your medications, if needed. They may evaluate the effectiveness of any prescribed medications, check your blood pressure and cholesterol levels, and make any necessary modifications to your medication regimen.

Lifestyle modifications and rehabilitation: Your healthcare provider may provide guidance on lifestyle modifications and rehabilitation exercises to optimize the benefits of PTA and improve your overall PAD management. They may discuss topics such as exercise routines, dietary changes, smoking cessation, and other lifestyle factors that can contribute to long-term improvement.

Prevention and risk factor management: Follow-up appointments are an opportunity for your healthcare provider to address any underlying risk factors for PAD and provide education on risk factor management. This may include discussions on managing blood pressure, cholesterol levels, diabetes, and other conditions that can contribute to PAD progression.

Addressing concerns and questions: Follow-up appointments also give you the opportunity to discuss any concerns, questions, or symptoms you may have related to your PAD or the PTA procedure. Open communication with your healthcare provider is important for ensuring that you receive appropriate care and support.

Remember, regular follow-up appointments are crucial for monitoring your progress, managing any potential complications, and optimizing your long-term outcomes. Be sure to attend these appointments as scheduled and communicate any changes or concerns to your healthcare provider.

14. Are there any lifestyle changes or ongoing treatments I should consider to maintain the benefits of PTA?

Yes, there are several lifestyle changes and ongoing treatments that can help maintain the benefits of percutaneous transluminal angioplasty (PTA) for peripheral arterial disease (PAD) and improve your overall vascular health. Here are some important considerations:

Adopt a healthy lifestyle: Making healthy lifestyle choices is crucial for managing PAD and maintaining the benefits of PTA. This includes:

Smoking cessation: If you smoke, quitting is essential for improving blood flow and reducing the risk of further arterial damage.

Regular exercise: Engaging in regular physical activity, as recommended by your healthcare provider, can help improve circulation, reduce symptoms, and enhance overall cardiovascular health. Walking, cycling, swimming, and other low-impact exercises are often recommended for individuals with PAD.

Healthy diet: Follow a balanced and nutritious diet that is low in saturated fats, cholesterol, and sodium. Include plenty of fruits, vegetables, whole grains, and lean proteins in your meals.

Weight management: Maintaining a healthy weight is important for reducing the strain on your cardiovascular system and improving PAD outcomes. If necessary, work with your healthcare provider or a registered dietitian to develop a personalized weight management plan.

Medication management: It is important to continue taking any prescribed medications as directed by your healthcare provider. This may include antiplatelet medications, statins, blood pressure medications, and other medications aimed at managing underlying conditions or symptoms associated with PAD. Adhering to your medication regimen can help reduce the risk of complications and maintain the benefits of PTA.

Regular follow-up care: Attend regular follow-up appointments with your healthcare provider to monitor your progress, assess the status of your PAD, and make any necessary adjustments to your treatment plan. These appointments are important for identifying any potential issues or changes in your condition and ensuring that you receive appropriate care and support.

Manage underlying risk factors: Take steps to manage underlying risk factors that contribute to PAD progression, such as high blood pressure, high cholesterol, diabetes, and obesity. Follow your healthcare provider's recommendations for managing these conditions, which may include lifestyle modifications, medication management, and regular monitoring.

Foot care: Individuals with PAD are at an increased risk of foot complications, including ulcers and infections. Pay close attention to foot care by keeping your feet clean, moisturized, and protected.

Regularly inspect your feet for any signs of injury or infection and seek prompt medical attention for any concerns.

Education and support: Stay informed about PAD and engage in patient education programs or support groups that can provide you with additional knowledge, guidance, and support. Learning about your condition and connecting with others who have experienced similar challenges can be beneficial for managing your health and well-being.

Remember, PAD is a chronic condition, and ongoing management is crucial for maintaining the benefits of PTA and minimizing the risk of complications or disease progression. Work closely with your healthcare provider to develop a comprehensive treatment plan that addresses your specific needs and follow their recommendations for lifestyle modifications, ongoing treatments, and self-care practices.
