# Supplementary material for: Feasibility of GPT-3 and GPT-4 for in-Depth Patient Education Prior to Interventional Radiological Procedures: A Comparative Analysis
Source: Cardiovasc Intervent Radiol. 2023 Oct 23;47(2):245–50. doi: 10.1007/s00270-023-03563-2 (PMC10844465; doi:10.1007/s00270-023-03563-2)
Supplement: Supplementary file 3 — Supplementary file3 (PDF 310 kb) [file 270_2023_3563_MOESM3_ESM.pdf]

GPT-3 TACE

General questions:

1. What is TACE?

TACE stands for Transarterial Chemoembolization. It is a minimally invasive procedure used in the treatment of certain types of liver cancer, specifically hepatocellular carcinoma (HCC). TACE combines two approaches: chemotherapy and embolization.

During a TACE procedure, a catheter is inserted through a small incision in the groin and threaded up to the blood vessels supplying the liver. Contrast dye is injected to visualize the blood vessels on X-ray imaging. Once the blood vessels feeding the tumor are identified, a mixture of chemotherapy drugs and embolic agents (particles) is injected directly into the blood vessels. The chemotherapy drugs target the tumor cells, while the embolic agents block the blood supply to the tumor, causing localized cell death.

TACE helps in shrinking the tumor, preventing its growth, and potentially extending the patient's survival. It is typically performed by an interventional radiologist in a specialized radiology suite.

2. What disease can be treated with TACE?

TACE (Transarterial Chemoembolization) is primarily used for the treatment of hepatocellular carcinoma (HCC), which is the most common type of primary liver cancer. HCC often occurs in individuals with underlying liver diseases such as cirrhosis, chronic hepatitis B or C infections, or non-alcoholic fatty liver disease.

TACE is generally recommended for patients with HCC who are not eligible for surgical resection (removal of the tumor) due to factors such as tumor size, location, or the presence of underlying liver disease that makes surgery risky. It can be used as a primary treatment option or in combination with other therapies such as ablation, targeted therapy, or as a bridge to liver transplantation.

In some cases, TACE may also be used for the treatment of liver metastases (cancer that has spread to the liver from another primary site), although its effectiveness may vary depending on the specific characteristics of the metastases and the primary cancer.

It's important to note that the decision to use TACE as a treatment option is made on a case-by-case basis, and the appropriateness of the procedure depends on factors such as the size, number, and location of tumors, as well as the overall health of the patient. The final decision should be made by a multidisciplinary team of healthcare professionals specialized in liver cancer treatment.

3. Which chemotherapeutic agents are used?

Several chemotherapeutic agents can be used in TACE (Transarterial Chemoembolization) for the treatment of liver cancer. The specific drugs utilized can vary depending on the preferences of the treating physician, institutional protocols, and the patient's individual characteristics. Here are some commonly used chemotherapy agents in TACE:

Doxorubicin: Doxorubicin is a potent chemotherapy drug that is frequently employed in TACE. It works by interfering with the replication of cancer cells and inhibiting their growth. Doxorubicin may be used alone or in combination with other agents.

Mitomycin C: Mitomycin C is another chemotherapy drug used in TACE. It functions by damaging the DNA in cancer cells, impairing their ability to divide and grow. Like doxorubicin, it can be used as a single agent or in combination with other drugs.

Cisplatin: Cisplatin is a platinum-based chemotherapy drug that can be utilized in TACE. It works by disrupting the DNA structure within cancer cells, leading to their destruction. Cisplatin may be employed alone or in combination with other agents.

Irinotecan: Irinotecan is a topoisomerase inhibitor that may be included in the chemotherapy regimen for TACE. It prevents cancer cells from properly replicating their DNA, ultimately causing their death.

It's important to note that the specific combination and dosage of chemotherapy agents used in TACE can vary depending on individual patient factors and the preferences of the treating medical team. The selection of chemotherapy drugs is based on their efficacy against liver cancer and their tolerability by the patient.

4. What is used for embolization?

During TACE (Transarterial Chemoembolization), embolization agents are used to block the blood vessels that supply the tumor in the liver. The embolization agents are introduced into the blood vessels along with the chemotherapy drugs to enhance the treatment's effectiveness. These agents can vary depending on the specific technique and the preference of the interventional radiologist performing the procedure. Here are some commonly used embolization agents in TACE:

Microspheres: Tiny particles called microspheres, often made of biocompatible materials such as polyvinyl alcohol (PVA), are commonly used for embolization in TACE. These microspheres block the blood flow to the tumor, depriving it of nutrients and oxygen, while also trapping the chemotherapy drugs within the tumor.

Embospheres: Embospheres are another type of microsphere commonly used for embolization. Similar to microspheres, they are composed of biocompatible materials and are designed to occlude the blood vessels feeding the tumor.

Gelfoam: Gelfoam is a sterile, absorbable gelatin sponge that can be cut into small pieces and used as an embolization agent. It is soaked in contrast dye and then injected into the blood vessels to create a blockage.

Polyvinyl alcohol (PVA) particles: PVA particles are small spherical embolic agents that are commonly used in TACE. They can be loaded with chemotherapy drugs before injection into the blood vessels supplying the tumor.

The choice of embolization agent depends on factors such as the size and location of the blood vessels, the desired level of embolization, and the experience and preference of the interventional radiologist. The goal of using embolization agents is to selectively block the blood supply to the tumor while minimizing damage to healthy liver tissue.

5. What are the success rates of TACE?

The success rates of TACE (Transarterial Chemoembolization) can vary depending on several factors, including the stage and characteristics of the liver cancer, the overall health of the patient, and the expertise of the medical team performing the procedure. Success rates are typically measured in terms of tumor response, overall survival, and progression-free survival. Here are some general considerations:

Tumor response: TACE has shown promising results in achieving tumor response or tumor shrinkage in patients with hepatocellular carcinoma (HCC). The success rate in terms of tumor response can vary but is reported to be around 30% to 60% of patients experiencing partial or complete tumor regression.

Overall survival: TACE has been demonstrated to improve overall survival in selected patients with HCC. The survival rates can vary widely depending on factors such as tumor stage, liver function, and patient comorbidities. Studies have reported median survival rates ranging from several months to over two years, depending on patient characteristics and tumor burden.

Progression-free survival: TACE can help delay the progression of liver cancer, providing a period of disease control. Progression-free survival rates vary, but studies have shown median progression-free survival ranging from several months to over a year.

It's important to note that success rates can vary among individuals, and not all patients will respond equally to TACE. Some patients may experience a significant reduction in tumor size and prolonged survival, while others may have a limited response or experience disease progression.

Furthermore, TACE is often used as part of a multimodal treatment approach for liver cancer, which may include other therapies such as surgery, ablation, targeted therapy, or liver transplantation. The success and effectiveness of TACE may be influenced by how it is integrated into the overall treatment strategy.

It's recommended to consult with a healthcare professional who can provide personalized information based on individual circumstances and available treatment options.

6. What are the contraindications for TACE?

While TACE (Transarterial Chemoembolization) is a generally safe and effective treatment option for liver cancer, there are certain contraindications or situations in which the procedure may not be suitable. Contraindications can vary depending on the specific patient and clinical factors, but here are some common contraindications for TACE:

Severe liver dysfunction: Patients with significantly impaired liver function, such as advanced cirrhosis or severe liver failure, may not be suitable candidates for TACE. The procedure requires adequate liver function to tolerate the effects of chemotherapy and embolization.

Inadequate liver reserve: TACE may not be recommended if the patient has limited liver reserve, indicated by poor synthetic function, severe portal hypertension, or significant ascites. Insufficient liver function can increase the risk of complications and limit the potential benefits of the procedure.

Vascular anatomy unsuitable for embolization: The presence of abnormal blood vessel anatomy or extensive collateral circulation may make it difficult to perform effective embolization. In such cases, TACE may not be feasible or may have limited efficacy.

Extrahepatic disease: TACE is primarily intended for treating liver tumors. If the cancer has spread extensively outside the liver (such as to distant organs), TACE may not provide substantial benefit and alternative treatment options may be considered.

Poor performance status: Patients with a significantly compromised overall health status, such as those with a very poor performance status or multiple comorbidities, may not be able to tolerate the procedure or may have limited life expectancy.

Allergy or intolerance to the chemotherapy agents or contrast media: If a patient has a known allergy or severe intolerance to the chemotherapy drugs used in TACE or the contrast media required for imaging, alternative treatment options should be explored.

It's important to note that the contraindications for TACE can vary based on individual patient factors, tumor characteristics, and the judgment of the healthcare team. The decision regarding TACE candidacy should be made by a multidisciplinary team of specialists, including hepatologists, interventional radiologists, and oncologists, who can assess the patient's overall condition and determine the most appropriate treatment approach.

7. What are alternatives to TACE?

There are several alternative treatment options available for liver cancer, particularly for patients who may not be suitable candidates for TACE (Transarterial Chemoembolization) or in cases where TACE has been ineffective. The choice of alternative treatment depends on various factors, including the stage of the disease, the extent of liver involvement, the overall health of the patient, and individualized considerations. Here are some common alternatives to TACE:

Surgical resection: Surgical resection involves the surgical removal of the tumor and surrounding healthy liver tissue. It is considered the primary treatment for early-stage liver cancer when the tumor is small and localized, and the liver function is adequate. Surgical resection aims to completely remove the tumor and potentially offers the best chance of cure.

Liver transplantation: Liver transplantation may be an option for select patients with liver cancer. It involves the surgical replacement of the diseased liver with a healthy liver from a deceased or living donor. Liver transplantation is suitable for patients with early-stage liver cancer who meet specific criteria, such as tumor size and number, absence of extrahepatic spread, and adequate liver function.

Ablation therapies: Ablation therapies use heat or cold to destroy the cancer cells. Techniques such as radiofrequency ablation (RFA), microwave ablation (MWA), and cryoablation can be utilized. These treatments are often recommended for small tumors and can be performed percutaneously (through the skin) or during laparoscopic surgery.

Targeted therapy: Targeted therapies are drugs that specifically target certain molecular pathways involved in cancer growth. They work by blocking the signals that promote tumor growth and survival. Drugs like sorafenib, lenvatinib, and regorafenib are examples of targeted therapies used for advanced liver cancer.

Systemic chemotherapy: Systemic chemotherapy involves the administration of anticancer drugs via the bloodstream to treat liver cancer that has spread beyond the liver. It may be used as a palliative treatment to slow down tumor growth, relieve symptoms, and improve overall quality of life.

Radiation therapy: External beam radiation therapy (EBRT) or internal radiation therapy (brachytherapy) may be used to deliver high-energy radiation to destroy cancer cells. Radiation therapy is commonly used for palliative purposes to alleviate symptoms and target specific areas of tumor growth.

Clinical trials: Participation in clinical trials investigating novel therapies or combination approaches is an option for some patients. Clinical trials can provide access to innovative treatments that are not yet widely available and contribute to advancing the field of liver cancer treatment.

The choice of alternative treatment depends on the individual patient's condition and should be made in consultation with a multidisciplinary team of healthcare professionals specialized in liver cancer management. They can assess the specific circumstances and recommend the most appropriate treatment approach.

8. What are treatment goals of TACE?

The treatment goals of TACE (Transarterial Chemoembolization) in the context of liver cancer management are multifaceted. The procedure is aimed at achieving several objectives, which can vary depending on the individual patient's condition and tumor characteristics. The primary treatment goals of TACE include:

Local tumor control: TACE aims to deliver chemotherapy drugs directly to the tumor site, selectively targeting cancer cells while minimizing systemic exposure. The chemotherapy drugs work to kill or inhibit the growth of tumor cells. The embolization component of TACE blocks the blood supply to the tumor, depriving it of nutrients and oxygen. Together, these actions help achieve local tumor control, shrink the tumor size, and prevent its further growth.

Prolongation of survival: TACE has been shown to improve overall survival in certain patients with hepatocellular carcinoma (HCC). By reducing tumor burden and preventing tumor progression, TACE can extend the patient's lifespan. It is often used as a palliative treatment to increase survival rates and improve quality of life.

Bridge to liver transplantation: In patients who are eligible for liver transplantation but have tumors that exceed the size criteria, TACE can be used as a bridge therapy. TACE helps control tumor growth and prevents the tumor from becoming unresectable or spreading further, allowing patients to remain eligible for transplantation.

Symptom relief: TACE can help alleviate symptoms associated with liver cancer, such as pain, discomfort, and digestive issues. By reducing tumor size and decreasing tumor-related pressure on surrounding tissues and organs, TACE can improve symptoms and enhance the patient's quality of life.

Downstaging for curative therapies: TACE can be employed as a neoadjuvant therapy to downstage large tumors, making them amenable to potentially curative treatments like surgical resection or liver transplantation. By reducing tumor size and controlling tumor growth, TACE improves the likelihood of successful curative interventions.

It's important to note that the treatment goals of TACE can vary depending on the patient's specific situation, tumor characteristics, and overall health status. The goals are individualized based on a comprehensive assessment by the medical team, taking into account the patient's unique circumstances and treatment objectives.

9. Can TACE cure cancer?

TACE (Transarterial Chemoembolization) is generally not considered a curative treatment for liver cancer. While TACE can be effective in achieving local tumor control, shrinking tumor size, and prolonging survival in certain cases, it is typically used as a palliative or disease-controlling therapy rather than a curative approach.

Curative treatment aims to completely eliminate cancer from the body, whereas TACE is primarily focused on managing the disease, slowing its progression, and improving quality of life.

However, in some cases, TACE can serve as a bridge therapy to potentially curative treatments such as liver transplantation or surgical resection. By controlling tumor growth and preventing tumor progression, TACE may help downsize tumors and make them amenable to these curative interventions. In such instances, TACE plays a supportive role in the overall treatment strategy.

It's worth noting that the long-term prognosis for liver cancer depends on various factors, including the stage of the disease, underlying liver health, tumor characteristics, and individual patient factors. In certain cases, a multimodal approach combining TACE with other treatments like surgery, ablation, targeted therapy, or systemic chemotherapy may offer the best chance of disease control and improved outcomes.

It's recommended to consult with a healthcare professional who can evaluate the specific circumstances and provide personalized information regarding the treatment options, potential benefits, and expected outcomes for an individual patient.

10. Can TACE be performed in addition to regular chemotherapy?

Yes, TACE (Transarterial Chemoembolization) can be performed in addition to regular chemotherapy in the management of liver cancer. In fact, TACE is a form of locoregional chemotherapy, where chemotherapy drugs are delivered directly to the tumor site.

Combining TACE with systemic chemotherapy can have synergistic effects and potentially enhance the overall treatment response. This approach is often referred to as combination therapy or multimodal treatment. The rationale behind combining TACE with systemic chemotherapy is to target the tumor through different mechanisms and increase the chances of tumor control.

Here are a few scenarios where TACE and systemic chemotherapy may be used together:

Neoadjuvant therapy: TACE may be used as a neoadjuvant therapy to downsize a large tumor before surgical resection or liver transplantation. Systemic chemotherapy can be administered prior to or following TACE to further shrink the tumor and reduce the risk of tumor recurrence.

Adjuvant therapy: After surgical resection or ablation of liver tumors, TACE can be utilized as an adjuvant therapy to target any remaining microscopic tumor cells that may not be detectable by imaging. Concurrent or subsequent systemic chemotherapy can be administered to target potential metastases or microsatellite lesions outside the liver.

Advanced or unresectable cases: In cases where liver cancer is advanced or deemed unresectable, a combination of TACE and systemic chemotherapy may be employed to control tumor growth, alleviate symptoms, and extend survival. TACE provides local tumor control, while systemic chemotherapy targets potential metastatic lesions outside the liver.

The specific treatment plan, including the combination of TACE and systemic chemotherapy, will depend on various factors, including the stage of the disease, liver function, patient's overall health, tumor characteristics, and the expertise of the medical team. A multidisciplinary approach involving oncologists, hepatologists, and interventional radiologists is often employed to design an optimal treatment strategy for each patient.

It's important to note that the choice and sequencing of treatments may vary for each individual, and it should be determined through discussions between the patient and the healthcare team based on the patient's specific condition and treatment goals.

Preparation and procedure:

1. How should I prepare for TACE?

Preparing for TACE (Transarterial Chemoembolization) involves several steps to ensure the procedure goes smoothly and to minimize any potential risks. Here are some general guidelines for preparing for TACE:

Medical evaluation: You will undergo a comprehensive medical evaluation to assess your overall health and suitability for the procedure. This may involve blood tests, imaging studies (such as CT scan or MRI), and liver function tests.

Fasting: Your healthcare team will provide specific instructions regarding fasting before the procedure. Typically, you will be asked to avoid eating or drinking anything for a certain period of time prior to TACE. This is done to reduce the risk of complications related to anesthesia or sedation.

Medication review: Inform your healthcare team about all the medications you are currently taking, including prescription drugs, over-the-counter medications, and any herbal supplements. They will provide instructions on which medications to continue or discontinue before the procedure.

Allergies and contrast media: Inform your healthcare team if you have any known allergies or sensitivities, especially to contrast media or any chemotherapy drugs that may be used during TACE. This information is crucial for your safety during the procedure.

Pre-procedure instructions: Your healthcare team will provide specific pre-procedure instructions, which may include guidelines on showering, dressing, and any necessary bowel preparation. Follow these instructions carefully to ensure a successful procedure.

Transportation arrangements: TACE is typically performed on an outpatient basis, but it's important to arrange for someone to accompany you and drive you home after the procedure. You may experience sedation or discomfort, making it unsafe for you to drive yourself.

Discussion with your healthcare team: Before TACE, have a detailed discussion with your healthcare team. They will explain the procedure, its potential risks and benefits, and address any concerns or questions you may have. Make sure you understand the procedure, its goals, and the expected recovery process.

It's essential to follow all the instructions provided by your healthcare team to ensure a successful TACE procedure. They will provide personalized instructions based on your specific medical condition and individual needs. If you have any concerns or questions during the preparation phase, don't hesitate to reach out to your healthcare team for clarification or further guidance.

2. Will I need to fast before the procedure?

Yes, fasting is typically required before undergoing TACE (Transarterial Chemoembolization). Fasting is necessary to reduce the risk of complications during the procedure, particularly those related to anesthesia or sedation. The specific fasting instructions may vary depending on the medical facility and the time of your scheduled procedure, so it's important to follow the instructions provided by your healthcare team. Here are some general guidelines regarding fasting before TACE:

NPO (Nil per os) status: You will be instructed to have nothing to eat or drink for a certain period of time before the procedure. This includes both solid foods and liquids, including water, coffee, tea, and juices.

Timeframe: Typically, you will be asked to fast for at least 6 hours before the procedure. However, the exact fasting duration may vary depending on your specific case and the policies of the medical facility. Your healthcare team will provide you with specific instructions regarding the timing of fasting.

Medication guidelines: Your healthcare team will provide instructions on whether to continue or temporarily discontinue any medications you are taking. Some medications may need to be taken with a small sip of water even during the fasting period, while others may need to be temporarily stopped. It's important to clarify these instructions with your healthcare team.

Clear liquids: In some cases, you may be allowed to have clear liquids up to a certain time before the procedure. Clear liquids typically refer to water, clear juices, and plain black coffee or tea without milk or cream. However, it's essential to follow the specific fasting guidelines provided by your healthcare team.

Anesthesia considerations: Fasting before TACE is necessary to minimize the risk of aspiration during the procedure. Aspiration occurs when stomach contents enter the lungs, which can be dangerous. Fasting helps ensure that the stomach is empty, reducing the likelihood of aspiration.

It's crucial to adhere to the fasting instructions provided by your healthcare team. Failing to fast as instructed may result in the postponement or cancellation of the procedure for your safety. If you have any questions or concerns about fasting or your specific fasting requirements, it's best to consult with your healthcare team for clarification and guidance.

3. Should I continue taking my medications before TACE?

Whether or not you should continue taking your medications before TACE (Transarterial Chemoembolization) depends on the specific medications you are taking and the instructions provided by your healthcare team. It's important to discuss this with your healthcare provider, who can provide personalized guidance based on your individual medical condition and the medications you are currently prescribed. Here are some general considerations:

Medication review: Make sure to inform your healthcare team about all the medications you are currently taking, including prescription drugs, over-the-counter medications, and any herbal supplements. This includes medications for any unrelated conditions or chronic illnesses you may have.

Instructions from your healthcare team: Your healthcare team will provide specific instructions regarding which medications to continue or discontinue before TACE. Some medications may need to be stopped temporarily, while others may need to be continued as usual. It's important to follow their guidance to ensure the procedure's success and minimize any potential risks.

Blood thinners: If you are taking blood-thinning medications (anticoagulants or antiplatelet drugs) such as aspirin, warfarin, clopidogrel, or others, your healthcare team will provide specific instructions on how to manage these medications before TACE. In some cases, blood thinners may need to be temporarily stopped to reduce the risk of bleeding during the procedure. However, it's

essential to follow the instructions provided by your healthcare team and not make any changes to your medication regimen without their guidance.

Communication with your healthcare team: If you have any concerns or questions about your medications before TACE, it's important to communicate with your healthcare team. They can address your specific situation, provide guidance on medication management, and ensure that your medications are appropriately adjusted or continued based on your individual needs.

Remember, every patient's situation is unique, and medication instructions can vary. It's crucial to consult with your healthcare team for personalized advice regarding your medications before TACE. They have a comprehensive understanding of your medical history and can provide you with specific instructions to follow to ensure your safety and optimize the effectiveness of the procedure.

4. Will I be awake during the procedure?

The level of consciousness during TACE (Transarterial Chemoembolization) can vary depending on the specific circumstances and the preferences of the patient and medical team. Typically, TACE is performed with the patient under conscious sedation or general anesthesia.

Conscious sedation: Conscious sedation is commonly used during TACE. It involves the administration of medications to induce a state of relaxation and drowsiness while keeping the patient conscious and able to respond to verbal cues. The sedative medications can help alleviate any discomfort or anxiety during the procedure. Local anesthesia may also be applied to numb the skin and tissues at the insertion site.

General anesthesia: In some cases, especially if the procedure is complex or lengthy, general anesthesia may be preferred. With general anesthesia, you will be completely unconscious and unaware during the entire procedure. The anesthesia team will administer medications to induce deep sleep, and a breathing tube may be inserted to assist with breathing.

The choice between conscious sedation and general anesthesia depends on various factors, including the patient's overall health, the complexity of the procedure, and the preferences of the patient and medical team. Your healthcare team will discuss the options with you and determine the most appropriate approach for your specific case.

During the procedure, regardless of the level of consciousness, your vital signs will be closely monitored, including blood pressure, heart rate, and oxygen levels. The medical team will also ensure your comfort and safety throughout the procedure.

It's important to have a discussion with your healthcare team prior to the procedure to understand the anesthesia options, their potential risks and benefits, and to address any concerns or questions you may have. They will provide you with specific information and instructions based on your individual needs and the planned approach for your TACE procedure.

5. How is the procedure performed?

TACE (Transarterial Chemoembolization) is typically performed by an interventional radiologist or an interventional oncologist. The procedure involves several steps, which may vary slightly depending on the specific case and the medical facility. Here is a general overview of how TACE is performed:

Pre-procedure preparation: You will be positioned on an examination table, typically lying on your back. Monitoring devices will be attached to track your vital signs, such as blood pressure, heart rate, and oxygen levels. An intravenous (IV) line may be inserted to administer fluids and medications during the procedure.

Anesthesia and sedation: Depending on the planned approach, conscious sedation or general anesthesia may be administered to ensure your comfort and minimize any discomfort or anxiety during the procedure. This will be discussed and determined prior to the procedure in consultation with your healthcare team.

Access and catheter placement: The interventional radiologist will access the blood vessels in your groin area (or, less commonly, in your wrist) using a small incision. A catheter (a thin, flexible tube) will be inserted through the incision and guided through the blood vessels using fluoroscopy (real-time X-ray imaging). The catheter is advanced to reach the arteries supplying the liver.

Angiography and tumor visualization: Once the catheter is in position, contrast dye will be injected through the catheter, and X-ray imaging (angiography) will be performed. This allows the interventional radiologist to visualize the blood vessels feeding the tumor and assess its size, location, and blood supply.

Chemotherapy drug administration: After angiography, a mixture of chemotherapy drugs, usually mixed with a contrast agent, will be slowly injected through the catheter directly into the blood vessels supplying the tumor. The chemotherapy drugs are intended to target and destroy the cancer cells in the liver.

Embolization: Following the chemotherapy drug infusion, embolization materials such as tiny particles, beads, or coils may be injected through the catheter to block the blood flow to the tumor and enhance the treatment's effectiveness. The embolization process helps cut off the tumor's blood supply, depriving it of oxygen and nutrients.

Completion and recovery: Once the TACE procedure is completed, the catheter will be removed, and the incision site will be closed with stitches or adhesive strips. You will be taken to a recovery area where you will be monitored for a period of time. Your vital signs and any potential post-procedure discomfort or complications will be closely observed.

The duration of the TACE procedure can vary depending on various factors, including the complexity of the case and the number of tumors being treated. It may take a few hours from the start of the procedure until you are transferred to the recovery area.

Following the procedure, your healthcare team will provide post-procedure instructions and discuss any potential side effects, risks, and expected recovery timeline. It's important to follow these instructions and attend any scheduled follow-up appointments to monitor your response to the treatment.

Please note that this is a general overview of the TACE procedure, and the specifics may vary based on your individual case. It's important to have a detailed discussion with your healthcare team to understand the procedure, its goals, and what to expect in your specific situation.

6. How long will the procedure take?

The duration of a TACE (Transarterial Chemoembolization) procedure can vary depending on various factors, including the complexity of the case, the number of tumors being treated, and individual patient factors. While it is difficult to provide an exact timeframe as it can vary from case to case, TACE procedures typically last between 1 to 3 hours.

The time required for a TACE procedure includes several steps, such as pre-procedure preparation, anesthesia or conscious sedation administration, access and catheter placement, angiography (X-ray imaging of blood vessels), chemotherapy drug infusion, embolization, and post-procedure recovery.

The complexity of the procedure can influence the duration. For example, if multiple tumors or large tumors are being treated, it may require more time for angiography and the administration of chemotherapy drugs and embolization materials.

It's important to note that the duration mentioned above primarily refers to the procedural time and does not include additional time for pre-procedure preparation, post-procedure recovery, or any necessary observation time before being discharged from the medical facility.

Your healthcare team will be able to provide you with a more accurate estimate of the expected duration for your specific TACE procedure based on your individual case and the planned approach. They will guide you through the process, provide updates during the procedure, and ensure that you are informed about the estimated duration and any potential variations.

It's essential to keep in mind that the duration of the procedure should not be a major concern. What matters most is that the medical team takes the necessary time to perform the procedure safely and effectively, addressing your specific needs and providing optimal care throughout the process.

7. Will I feel any pain during TACE?

During TACE (Transarterial Chemoembolization), measures are taken to minimize discomfort and pain. The specific level of discomfort or pain experienced during the procedure can vary from person to person, and it depends on factors such as the individual's pain threshold, the use of anesthesia or conscious sedation, and the complexity of the case. Here are some important points to consider:

Anesthesia or conscious sedation: Prior to the procedure, you will receive anesthesia or conscious sedation to ensure your comfort during TACE. Conscious sedation involves the administration of medications that induce a state of relaxation and drowsiness while keeping you conscious and able to respond to verbal cues. This helps minimize any discomfort or anxiety during the procedure. In some cases, general anesthesia may be used, in which you will be completely unconscious and unaware during the procedure.

Local anesthesia: Local anesthesia is often applied to numb the skin and tissues at the insertion site where the catheter is inserted. This helps minimize any pain or discomfort associated with the initial incision and catheter placement.

Communication with the medical team: During the procedure, it is important to communicate with the medical team if you experience any pain or discomfort. They can adjust the sedation or provide additional measures to alleviate any discomfort.

Pain management: If you experience any pain or discomfort following the TACE procedure, your healthcare team will provide appropriate pain management strategies. This may include prescribing pain medications or recommending over-the-counter pain relievers to help manage any post-procedure discomfort.

It's important to remember that TACE is a minimally invasive procedure, and steps are taken to ensure your comfort throughout the process. While some individuals may experience mild discomfort or pressure sensations during the procedure, severe pain is not typically expected. The use of anesthesia or conscious sedation aims to keep you comfortable and minimize any potential pain or anxiety.

Before the procedure, it is crucial to have a discussion with your healthcare team to understand their pain management approach and to express any concerns or questions you may have about pain control during and after the procedure. They will be able to provide personalized guidance based on your specific situation and help ensure that you are as comfortable as possible during TACE.

8. Will I be able to feel or sense anything during the TACE?

During TACE (Transarterial Chemoembolization), the level of awareness or sensory perception can vary depending on the type of anesthesia or conscious sedation used. Here are some points to consider regarding your sensory experience during TACE:

Conscious sedation: Conscious sedation is commonly used during TACE. It involves the administration of medications that induce a state of relaxation and drowsiness while keeping you conscious and able to respond to verbal cues. With conscious sedation, you may feel relaxed and sleepy during the procedure, but you should not experience any significant pain or discomfort. While you may be aware of some sensations, you are unlikely to have a detailed memory of the procedure afterward.

General anesthesia: In some cases, general anesthesia may be used for TACE. With general anesthesia, you will be completely unconscious and unaware during the procedure. This means you will not feel or sense anything during the TACE procedure. General anesthesia is typically used for more complex cases or if conscious sedation is not suitable for the patient.

Local anesthesia: Local anesthesia is often applied to numb the skin and tissues at the insertion site where the catheter is inserted. This helps ensure that you do not feel pain or discomfort associated with the initial incision and catheter placement.

It's important to have a discussion with your healthcare team before the procedure to understand the type of anesthesia or conscious sedation that will be used and its expected effects. They will provide information specific to your case and address any concerns or questions you may have.

During the procedure, your vital signs will be continuously monitored, and the medical team will be attentive to your comfort and well-being. If you have any sensations or discomfort during the procedure, it's important to communicate with the medical team. They can adjust the sedation or provide additional measures to ensure your comfort.

Remember, the goal of anesthesia and conscious sedation during TACE is to keep you comfortable and pain-free while the procedure is performed. Your healthcare team will strive to provide the appropriate level of sedation to minimize any sensations or discomfort you may experience.

9. What type of anesthesia or sedation can be used during TACE?

During TACE (Transarterial Chemoembolization), different types of anesthesia or sedation can be used to ensure your comfort and minimize any pain or discomfort during the procedure. The specific type of anesthesia or sedation employed depends on various factors, including your medical condition, the complexity of the case, and your preferences. Here are the common options:

Conscious sedation: Conscious sedation is a commonly used approach for TACE. It involves the administration of medications to induce a state of relaxation and drowsiness while keeping you conscious and able to respond to verbal cues. Typically, a combination of medications, such as sedatives and pain relievers, is administered through an intravenous (IV) line. Conscious sedation helps alleviate any anxiety or discomfort during the procedure while allowing you to remain responsive to instructions from the medical team.

General anesthesia: In some cases, general anesthesia may be preferred or necessary for TACE. With general anesthesia, you will be completely unconscious and unaware during the procedure. Anesthesia medications are administered to induce a state of deep sleep, and a breathing tube may be inserted to assist with breathing. General anesthesia is often used for more complex or lengthy procedures, or if conscious sedation is not suitable due to specific patient factors.

The choice between conscious sedation and general anesthesia is typically made based on your medical condition, the complexity of the procedure, your preferences, and the expertise and recommendation of your healthcare team. They will evaluate your case and determine the most appropriate approach to ensure your safety, comfort, and optimal outcome.

It's important to have a thorough discussion with your healthcare team prior to the procedure to understand the anesthesia or sedation options available, their potential risks and benefits, and to address any concerns or questions you may have. They will provide you with personalized information and recommendations based on your individual needs and the planned approach for your TACE procedure.

During the procedure, regardless of the type of anesthesia or sedation used, your vital signs will be continuously monitored, and the medical team will ensure your comfort and safety throughout the process.

10. How will I be monitored during the procedure?

During a TACE (Transarterial Chemoembolization) procedure, you will be closely monitored to ensure your safety and well-being. Monitoring is an essential part of the procedure and involves assessing vital signs and other parameters throughout the process. Here are the typical monitoring methods used during TACE:

Blood pressure monitoring: Your blood pressure will be measured regularly throughout the procedure. This is usually done using an automatic blood pressure cuff placed on your arm. Blood pressure readings help evaluate your cardiovascular health and ensure that your blood pressure remains within a safe range.

Heart rate monitoring: Your heart rate will be continuously monitored using electrocardiography (ECG). Electrodes will be placed on your chest to record the electrical activity of your heart. Monitoring heart rate helps assess your heart's function and detect any irregularities or changes that may require attention.

Oxygen saturation monitoring: Oxygen saturation levels in your blood will be monitored using a pulse oximeter. A small device will be attached to your finger or earlobe, which measures the amount of oxygen carried by your red blood cells. This helps ensure that you are receiving adequate oxygen supply during the procedure.

Respiratory monitoring: Your respiratory rate will be monitored to assess your breathing pattern and ensure it remains stable. This can be done visually or with the help of a respiratory monitoring device.

Consciousness and response monitoring: If conscious sedation is used, the medical team will closely monitor your level of consciousness and responsiveness throughout the procedure. They will interact with you and ask questions to ensure you are appropriately sedated and comfortable.

Additionally, the medical team will be present throughout the procedure, closely observing you and attending to any changes or concerns. They will be available to address any discomfort or pain you may experience and adjust the level of sedation or provide additional support as needed.

Monitoring during TACE is crucial to detect any potential complications or adverse reactions promptly. It ensures that your vital signs and overall condition remain stable throughout the procedure. The medical team is trained to interpret these monitoring parameters and respond promptly to any issues that may arise.

It's important to communicate with your healthcare team throughout the procedure and inform them of any discomfort or concerns you may have. They are there to support you and ensure your well-being during the TACE procedure.

Risks and Complications:

1. What are the possible risks and complications of TACE?

While TACE (Transarterial Chemoembolization) is generally considered a safe procedure, there are potential risks and complications associated with it. These can vary depending on individual factors, the specific case, and the overall health of the patient. It's important to have a thorough discussion with your healthcare team to understand the potential risks and benefits in your specific situation. Here are some possible risks and complications of TACE:

Post-embolization syndrome: After the TACE procedure, it is common to experience a temporary condition called post-embolization syndrome. This can include symptoms such as fever, pain, nausea, vomiting, loss of appetite, fatigue, and general malaise. These symptoms are usually self-limiting and resolve within a few days to weeks with appropriate supportive care.

Pain or discomfort: Some individuals may experience pain or discomfort at the site of the catheter insertion or in the liver region after TACE. This can typically be managed with pain medications and usually subsides over time.

Infection: There is a risk of infection associated with any invasive procedure. Steps are taken to minimize this risk, such as using sterile techniques during the procedure. However, infection can still occur, requiring medical intervention and possibly antibiotic treatment.

Bleeding: There is a small risk of bleeding at the catheter insertion site or within the liver during the procedure. In rare cases, significant bleeding may require additional medical interventions, including blood transfusion or surgical management.

Allergic reactions: Some individuals may experience allergic reactions to the contrast dye used during angiography. These reactions can range from mild to severe and may require medical treatment. Inform your healthcare team about any known allergies or previous reactions to contrast agents.

Damage to healthy liver tissue: While TACE is targeted to treat liver tumors, there is a possibility of damage to surrounding healthy liver tissue. The goal is to minimize this by precisely directing the chemotherapy and embolization agents to the tumor-feeding blood vessels. However, some healthy liver tissue may still be affected, and this can result in transient or, rarely, long-term liver function abnormalities.

Blood vessel injury: There is a small risk of injury to the blood vessels during catheter placement or angiography. In rare cases, this may require further intervention, such as vascular repair or embolization.

Non-target embolization: Occasionally, embolization materials may inadvertently flow to non-target areas, leading to unintended embolization and potential complications. Care is taken to minimize this risk, but it can still occur.

Rare complications: Rare but serious complications can include liver abscess, bile duct injury, gastrointestinal ulceration or perforation, kidney injury, and pulmonary embolism. These complications are uncommon but can require further medical interventions.

It's important to note that the risks and complications mentioned above are not exhaustive, and there may be other specific risks depending on individual cases. Your healthcare team will evaluate your specific situation, provide information on the potential risks and benefits, and take measures to minimize the risks associated with TACE.

Before the procedure, make sure to discuss any concerns or questions you may have with your healthcare team. They will provide you with detailed information about the potential risks and complications specific to your case and help you make an informed decision about the procedure.

2. How common are these risks and complications?

The incidence of risks and complications associated with TACE (Transarterial Chemoembolization) can vary depending on several factors, including the patient population, underlying medical conditions, tumor characteristics, and the expertise of the medical team performing the procedure. It's

important to note that while risks and complications can occur, they are generally considered infrequent or rare. Here is a general overview of the commonality of these risks and complications:

Post-embolization syndrome: Post-embolization syndrome, which includes symptoms such as fever, pain, nausea, and fatigue, is a common occurrence after TACE. It is estimated to occur in a significant proportion of patients, typically resolving within a few days to weeks with supportive care.

Pain or discomfort: Pain or discomfort at the catheter insertion site or in the liver region after TACE is common but generally manageable with pain medications. The severity and duration of pain can vary among individuals.

Infection: While infection is a potential risk, the incidence of infection following TACE is relatively low. Proper sterile techniques and precautions are taken to minimize this risk during the procedure.

Bleeding: Bleeding at the catheter insertion site or within the liver during TACE is uncommon but can occur. Significant bleeding requiring intervention is rare.

Allergic reactions: Allergic reactions to contrast dye used during angiography are relatively rare but can occur. The incidence of severe allergic reactions is low.

Damage to healthy liver tissue: TACE is designed to target liver tumors while minimizing damage to healthy liver tissue. However, some degree of injury to surrounding liver tissue is possible. Severe or long-term liver function abnormalities are rare.

Blood vessel injury: While rare, blood vessel injury can occur during catheter placement or angiography. Proper techniques and expertise minimize this risk.

Non-target embolization: Non-target embolization is an infrequent occurrence but can happen. The medical team takes precautions to minimize this risk through careful catheter navigation and embolization agent delivery.

Rare complications: Rare complications such as liver abscess, bile duct injury, gastrointestinal ulceration or perforation, kidney injury, and pulmonary embolism are uncommon but can occur.

It's important to emphasize that the incidence of these risks and complications may vary, and the information provided here serves as a general guideline. The specific occurrence rates can differ depending on multiple factors and should be discussed with your healthcare team. They will provide you with more precise information based on their experience and your individual circumstances.

Your healthcare team will take precautions, closely monitor your condition during and after the procedure, and provide appropriate interventions or treatments if any complications arise. The goal is to maximize the benefits of TACE while minimizing the potential risks and complications.

3. Are there any specific factors that increase my risk of complications?

Several factors can increase the risk of complications associated with TACE (Transarterial Chemoembolization). It's important to discuss your individual risk factors with your healthcare team, as they can provide more personalized information based on your specific situation. Here are some general factors that may increase the risk of complications:

Liver function: If you have significant underlying liver dysfunction or impaired liver function, such as advanced cirrhosis or severe hepatocellular insufficiency, you may be at higher risk for complications after TACE. This is because the liver plays a crucial role in metabolizing drugs and managing the body's response to embolization. Your healthcare team will assess your liver function before the procedure to determine the appropriateness and potential risks associated with TACE.

General health status: Your overall health and medical history can impact the risk of complications. Patients with pre-existing medical conditions, such as heart disease, lung disease, kidney disease, or diabetes, may have a higher risk of experiencing complications during or after TACE. It's important to provide your healthcare team with a complete and accurate medical history to help them assess your individual risk factors.

Tumor characteristics: The size, location, and vascularity of the liver tumor being targeted can influence the risk of complications during TACE. Large or complex tumors, tumors close to critical structures, or tumors with an extensive blood supply may pose a higher risk. Your healthcare team will consider these factors when evaluating the feasibility and potential risks of TACE in your case.

Previous treatments: If you have undergone prior liver interventions or treatments, such as surgery, radiofrequency ablation, or previous TACE procedures, it may affect the risk profile for subsequent TACE. Scar tissue or altered blood supply in the liver can complicate the procedure and increase the risk of complications.

Coagulation disorders: Patients with underlying coagulation disorders or those taking anticoagulant medications may have an increased risk of bleeding during or after TACE. Your healthcare team will assess your coagulation profile and may adjust your medications or take additional precautions to minimize bleeding risks.

Age and general fitness: Advanced age and poor general physical fitness can potentially increase the risk of complications associated with any medical procedure, including TACE. Older adults or individuals with limited functional capacity may have a higher risk of adverse events or slower recovery.

It's crucial to have a detailed discussion with your healthcare team before the procedure to assess your individual risk factors and determine the appropriateness of TACE in your case. They will evaluate your specific situation, consider any potential risks, and take necessary precautions to maximize the benefits and minimize complications.

4. What steps are taken to minimize the risks during the procedure?

During a TACE (Transarterial Chemoembolization) procedure, several steps are taken to minimize the risks and ensure patient safety. These steps involve careful planning, meticulous technique, monitoring, and prompt intervention if any complications arise. Here are some measures commonly employed to minimize risks during TACE:

Comprehensive pre-procedural evaluation: Before the procedure, a thorough evaluation of your medical history, physical examination, and imaging studies (such as CT scans or MRI) is conducted. This helps identify any factors that may increase the risk of complications, allowing the medical team to tailor the procedure to your specific needs.

Patient selection and case assessment: The medical team carefully selects appropriate candidates for TACE based on factors such as tumor characteristics, liver function, and overall health status. This ensures that the benefits of the procedure outweigh the potential risks.

Expertise and experience: TACE procedures are typically performed by interventional radiologists or interventional oncologists who have specialized training and experience in these techniques. Their expertise helps minimize complications and optimize treatment outcomes.

Sterile techniques: Strict sterile techniques are employed during the procedure to minimize the risk of infection. This includes thorough handwashing, the use of sterile drapes, gloves, masks, and sterile instruments.

Imaging guidance: TACE is performed under imaging guidance, such as fluoroscopy, ultrasound, or CT scan, to ensure accurate catheter placement and precise targeting of the tumor-feeding blood vessels. Real-time imaging helps minimize the risk of inadvertent embolization or damage to surrounding structures.

Careful catheter navigation: The catheter is navigated through the blood vessels under fluoroscopic guidance to reach the tumor-feeding vessels while avoiding non-target areas. The use of selective catheters and microcatheters allows for more precise targeting and reduces the risk of non-target embolization.

Monitoring during the procedure: Vital signs, such as blood pressure, heart rate, oxygen saturation, and respiratory rate, are continuously monitored during the procedure. This helps detect any changes or complications promptly, allowing for immediate intervention if needed.

Medication administration: Chemotherapeutic agents and embolization materials are carefully selected and administered based on the individual case. Dosing and drug selection are tailored to the patient's specific needs, aiming to maximize therapeutic efficacy while minimizing adverse effects.

Prompt management of complications: In the event of any complications during or after the procedure, the medical team is prepared to provide timely intervention. This may include addressing pain or discomfort, managing bleeding or other adverse events, and ensuring appropriate post-procedural care.

Post-procedural monitoring and follow-up: After TACE, close monitoring and follow-up are conducted to assess the response to treatment, manage any post-procedural symptoms, and evaluate for potential complications. This allows for timely intervention and adjustment of the treatment plan as necessary.

These steps, along with adherence to established guidelines and protocols, help minimize the risks associated with TACE and ensure the safety and well-being of the patient throughout the procedure. Your healthcare team will discuss these measures with you and address any specific concerns you may have.

5. Can my liver be damaged?

During a TACE (Transarterial Chemoembolization) procedure, there is a potential risk of liver damage, although steps are taken to minimize this risk. TACE is designed to target liver tumors while sparing healthy liver tissue as much as possible. However, some degree of injury to surrounding liver tissue can occur. Here are some factors to consider regarding potential liver damage:

Selective targeting: TACE aims to deliver the chemotherapeutic agents directly to the tumor-feeding blood vessels, thereby concentrating the treatment in the tumor and minimizing exposure to healthy liver tissue. This selective targeting helps reduce the risk of damage to the healthy liver.

Blood supply variations: The blood supply to liver tumors can vary among individuals. In some cases, the blood vessels supplying the tumor may be intertwined with those supplying healthy liver tissue. While efforts are made to selectively embolize tumor vessels, there is a possibility of affecting nearby healthy liver tissue. However, the liver has a remarkable capacity for regeneration, and any potential damage to healthy tissue is usually transient and manageable.

Pre-procedural evaluation: Before the TACE procedure, a comprehensive evaluation is conducted to assess liver function and identify any pre-existing liver diseases or conditions that may impact the

risks and potential for liver damage. This helps the healthcare team customize the treatment plan and minimize the risk of complications.

Expertise and precision: TACE procedures are typically performed by experienced interventional radiologists or interventional oncologists who have specialized training in these techniques. Their expertise in catheter navigation, embolization, and drug delivery helps ensure accurate placement and minimizes the risk of unintended damage to the liver.

Monitoring and follow-up: Close monitoring is conducted during and after the TACE procedure to assess liver function and detect any potential complications. This allows for timely intervention if needed. Additionally, regular follow-up visits and imaging studies are conducted to evaluate treatment response and monitor the long-term effects on liver health.

It's important to discuss the potential risks and benefits of TACE with your healthcare team. They will consider your individual circumstances, including your liver function and tumor characteristics, to determine the appropriateness of the procedure and help you make an informed decision. While there is a potential for liver damage, the overall goal of TACE is to achieve tumor control while preserving liver function and quality of life. Your healthcare team will work to minimize risks and provide optimal care throughout the treatment process.

6. Will my liver function be impaired after the procedure?

After a TACE (Transarterial Chemoembolization) procedure, there is a possibility of transient impairment of liver function. However, the extent and duration of any potential impairment can vary among individuals and depend on several factors, including the underlying liver health, the extent of the procedure, and the individual response to treatment. Here are some important considerations:

Selective targeting: TACE aims to selectively deliver chemotherapeutic agents and embolic materials to the tumor-feeding blood vessels while minimizing exposure to healthy liver tissue. This selective targeting helps reduce the risk of widespread liver damage and preserves overall liver function.

Temporary liver function changes: The embolization and chemotherapeutic agents used in TACE may cause a temporary alteration in liver function. This can manifest as transient elevations in liver enzyme levels, such as alanine aminotransferase (ALT) and aspartate aminotransferase (AST), which are commonly monitored after the procedure. These changes are typically self-limiting and resolve over time.

Pre-existing liver disease: Patients with pre-existing liver diseases, such as cirrhosis or impaired liver function, may be more susceptible to temporary impairment of liver function after TACE. The extent of liver function changes can depend on the severity of the underlying liver disease.

Individual response: The response to TACE can vary among individuals. Some patients may experience minimal impact on liver function, while others may have a more pronounced transient impairment.

Regular monitoring and follow-up visits will help assess liver function and detect any significant changes.

Liver regeneration: The liver has a remarkable capacity for regeneration. In most cases, any temporary liver function impairment after TACE is reversible as the liver regenerates and recovers. The healthy liver tissue typically regains its function over time.

Long-term liver function: In the majority of cases, TACE is performed with the intention of preserving liver function while targeting the tumor. The overall goal is to achieve tumor control while maintaining liver health. However, it's important to monitor liver function in the long term, especially in patients with pre-existing liver disease or a history of multiple TACE procedures.

It's crucial to have open and regular communication with your healthcare team regarding any concerns about liver function. They will assess your specific situation, provide appropriate pre- and post-procedural care, monitor liver function, and guide you through the recovery process. If you have underlying liver disease, they may work in collaboration with a hepatologist or liver specialist to optimize your care. Your healthcare team will take measures to minimize liver-related complications and ensure the best possible outcomes.

7. Can I die during the procedure?

While any medical procedure carries some level of risk, including the possibility of serious complications, the risk of death during a TACE (Transarterial Chemoembolization) procedure is generally considered low. TACE is a minimally invasive procedure performed under careful medical supervision, and steps are taken to minimize risks and ensure patient safety. Here are some important points to consider:

Pre-procedural evaluation: Before undergoing TACE, a thorough evaluation is conducted to assess your overall health, including cardiac function, respiratory function, liver function, and any other relevant factors. This evaluation helps identify any potential risks or contraindications that may increase the likelihood of serious complications.

Selection of appropriate candidates: TACE is typically recommended for patients who are considered suitable candidates based on their underlying health condition, liver function, tumor characteristics, and overall treatment goals. Patients who are at higher risk for severe complications or have contraindications may be advised against TACE.

Experienced medical team: TACE procedures are performed by interventional radiologists or interventional oncologists who have specialized training and experience in these techniques. Their expertise helps minimize the risk of complications and allows for prompt intervention if any unexpected events occur.

Monitoring during the procedure: You will be continuously monitored throughout the TACE procedure, including vital signs such as blood pressure, heart rate, oxygen saturation, and respiratory rate. This monitoring allows the medical team to promptly detect and address any changes in your condition.

Adherence to safety protocols: Strict adherence to established safety protocols, including aseptic techniques, proper medication administration, and careful catheter navigation, helps minimize the risk of complications during the procedure.

Emergency preparedness: The medical team is equipped and trained to manage any potential complications that may arise during or immediately after the procedure. Emergency equipment, resuscitation measures, and medications are readily available to address any unforeseen events.

It's important to understand that every medical procedure carries some degree of risk, and the specific risks can vary depending on individual factors. While the risk of death during a TACE procedure is generally low, it's crucial to have open and honest discussions with your healthcare team about the potential risks, benefits, and alternatives of the procedure. They will assess your specific situation, address your concerns, and provide you with the necessary information to make an informed decision about your treatment.

8. Are there any complications that can occur after the procedure?

Yes, there are potential complications that can occur after a TACE (Transarterial Chemoembolization) procedure. While these complications are generally infrequent, it's important to be aware of them. The specific complications can vary depending on individual factors, the extent of the procedure, and the response to treatment. Here are some possible complications that can occur after TACE:

Post-embolization syndrome: It is common for patients to experience a temporary syndrome after TACE, known as post-embolization syndrome. This can include symptoms such as pain or discomfort in the abdomen, fever, nausea, vomiting, fatigue, and loss of appetite. These symptoms usually resolve within a few days to a week with supportive care and medication.

Liver function changes: As the liver processes the chemotherapeutic agents and responds to the embolization, there may be transient changes in liver function. This can manifest as temporary elevation of liver enzymes, such as alanine aminotransferase (ALT) and aspartate aminotransferase (AST). These changes typically resolve over time, but close monitoring of liver function is important.

Infection: There is a risk of infection at the site of catheter insertion or in the liver following the TACE procedure. Antibiotics may be given before or after the procedure to reduce this risk. It is essential to monitor for signs of infection, such as fever, increasing pain, or drainage from the insertion site.

Bleeding or hematoma: There is a small risk of bleeding or hematoma (collection of blood) at the catheter insertion site or within the liver. Close monitoring and appropriate management can help mitigate this risk.

Non-target embolization: In some cases, the embolic materials used during TACE can inadvertently migrate to non-target areas, leading to unintended blockage of blood vessels and potential damage to healthy tissues. This risk is minimized through meticulous technique and careful catheter navigation.

Liver abscess: In rare cases, a liver abscess can develop as a result of infection in the liver following TACE. Symptoms may include fever, abdominal pain, and jaundice. Prompt medical attention and appropriate treatment are necessary if a liver abscess is suspected.

Hepatic decompensation: In patients with underlying liver disease, TACE can potentially lead to hepatic decompensation, which is a significant deterioration of liver function. Close monitoring of liver function, as well as collaboration with a hepatologist or liver specialist, is important for those with pre-existing liver disease.

Allergic reactions: Although uncommon, allergic reactions to the contrast dye or medications used during the procedure can occur. These reactions can range from mild symptoms, such as rash or itching, to severe reactions requiring immediate medical intervention.

It's important to discuss these potential complications with your healthcare team before undergoing TACE. They will provide you with detailed information about the possible risks, preventive measures, and post-procedural care. Regular follow-up visits and close monitoring after the procedure will help detect and manage any complications that may arise.

9. Are there any long-term risks or complications associated with TACE?

Long-term risks and complications associated with Transarterial Chemoembolization (TACE) are generally infrequent, but it's important to be aware of potential considerations. The specific risks can vary depending on individual factors, the underlying condition being treated, and the response to treatment. Here are some possible long-term risks and complications associated with TACE:

Liver damage: While efforts are made to selectively target the tumor and minimize damage to healthy liver tissue, there is a potential risk of liver damage. In some cases, the treatment may affect a small portion of healthy liver tissue surrounding the tumor. However, the liver has a remarkable capacity for regeneration, and any potential damage to healthy tissue is usually transient and manageable.

Recurrence or progression of tumors: TACE is aimed at controlling or reducing the size of liver tumors. However, there is a possibility of tumor recurrence or progression over time, particularly in cases where the underlying liver disease is not curable. Regular monitoring and follow-up imaging studies are important to detect any signs of tumor recurrence or progression.

Impaired liver function: In patients with underlying liver disease, TACE can potentially contribute to further impairment of liver function. The impact on liver function depends on the extent of liver disease and the response to treatment. Regular monitoring of liver function, collaboration with a hepatologist or liver specialist, and appropriate management are important in these cases.

Non-target embolization: Although rare, there is a risk of non-target embolization, where the embolic materials used during TACE inadvertently migrate to non-target areas of the liver or other organs. This can potentially cause damage to healthy tissues or compromise the blood supply to these areas.

Biliary complications: TACE may cause inflammation or injury to the bile ducts, which are responsible for transporting bile from the liver. This can lead to biliary complications such as bile duct strictures, bile leaks, or jaundice. These complications are relatively rare but may require further intervention or management.

Radiation-related complications: In some cases, TACE may involve the use of radiation-emitting agents, such as Yttrium-90 microspheres. While this can be an effective treatment approach for certain liver tumors, there is a small risk of radiation-related complications, such as radiation-induced liver disease (RILD) or radiation pneumonitis (if the radiation reaches the lungs). Pre-treatment evaluation and careful patient selection are important to minimize these risks.

It's important to have open and regular communication with your healthcare team about the potential long-term risks and complications associated with TACE. They will provide you with detailed information based on your specific situation, monitor your progress, and help manage any concerns that may arise. Regular follow-up visits and imaging studies are crucial for monitoring treatment response, detecting any potential complications, and ensuring optimal long-term outcomes.

10. Does the cytostatic medication have a systemic effect?

Yes, the cytostatic medication used during Transarterial Chemoembolization (TACE) can have a systemic effect to some extent. Although TACE is a targeted treatment that delivers chemotherapy directly to the tumor through the tumor-feeding blood vessels, a portion of the medication can enter the systemic circulation. Here are some key points to understand about the systemic effects of cytostatic medication in TACE:

Localized delivery: The primary goal of TACE is to deliver the chemotherapeutic agents directly to the tumor site, where they can exert their anti-cancer effects. By selectively injecting the medication into the tumor-feeding blood vessels, TACE aims to concentrate the chemotherapy within the tumor, leading to local tumor control.

Systemic circulation: During the TACE procedure, a small portion of the chemotherapeutic agents can enter the systemic circulation and be distributed throughout the body. This occurs because the tumor-feeding blood vessels may have connections with other blood vessels supplying normal tissues.

Lower systemic exposure: Compared to traditional systemic chemotherapy, the systemic exposure to cytostatic medication in TACE is generally lower. This is due to the preferential targeting of the tumor vasculature and the use of embolic materials to restrict blood flow to the tumor.

Potential systemic effects: The systemic exposure to cytostatic medication in TACE can result in some systemic effects. However, these effects are usually less pronounced compared to systemic chemotherapy. Systemic side effects such as bone marrow suppression, hair loss, and gastrointestinal disturbances are typically milder with TACE, as the concentration of medication reaching normal tissues is lower.

Individual response: The systemic effects can vary among individuals, depending on factors such as the specific chemotherapy drugs used, the dosage, the extent of embolization, and the individual's overall health. Some patients may experience mild systemic side effects, while others may have minimal or no noticeable effects.

It's important to note that the primary focus of TACE is the localized treatment of liver tumors, and systemic effects are typically considered secondary. The goal is to maximize tumor response while minimizing systemic toxicity. Close monitoring and regular follow-up visits with your healthcare team will help assess your individual response, manage any potential side effects, and optimize your treatment outcomes.

It's recommended to discuss the specific systemic effects and potential side effects of the chemotherapy agents used in TACE with your healthcare team. They will provide you with detailed information based on your specific treatment plan and address any concerns you may have.

11. Will I lose my hair?

Hair loss is generally not a common side effect of Transarterial Chemoembolization (TACE) because the chemotherapy drugs used in TACE are primarily targeted to the tumor site and have a lower systemic exposure compared to traditional systemic chemotherapy. However, it's important to note that hair loss can occur as a side effect of certain chemotherapy agents, and in some cases, these agents may be used in combination with TACE.

The likelihood of experiencing hair loss during TACE depends on various factors, including the specific chemotherapy drugs used, the dosage, and your individual response. If the chemotherapy drugs used in your TACE treatment have the potential to cause hair loss, your healthcare team will inform you about this possibility during the treatment planning process.

It's important to have open and honest discussions with your healthcare team regarding the potential side effects of the specific chemotherapy agents used in your TACE procedure. They can provide you with more accurate information based on your individual treatment plan and help you understand the likelihood of hair loss or other side effects associated with the medications used.

If hair loss does occur, it is typically temporary, and the hair usually starts to regrow once the chemotherapy treatment is completed. However, the regrowth process may take some time, and the texture or color of the regrown hair may differ temporarily.

If the possibility of hair loss is a concern for you, consider discussing it with your healthcare team. They may be able to provide further guidance, suggest supportive measures to minimize hair loss, or recommend resources for coping with hair loss during your treatment journey.

Postinterventional aftercare:

1. What should I consider after TACE?

After undergoing Transarterial Chemoembolization (TACE), there are several important considerations to keep in mind for a smooth recovery and optimal post-operative care. Here are some key points to consider:

Post-operative instructions: Follow the specific post-operative instructions provided by your healthcare team. These instructions may include information on wound care, medication management, diet restrictions, activity limitations, and when to schedule follow-up appointments.

Recovery period: Give yourself adequate time to rest and recover after the TACE surgery. The duration of the recovery period can vary depending on individual factors, the extent of the procedure, and your overall health. Listen to your body and gradually increase your activity level as recommended by your healthcare team.

Pain management: You may experience some pain or discomfort after the TACE surgery. Your healthcare team will provide instructions on pain management, which may include the use of over-the-counter pain relievers or prescription medications. Follow the prescribed dosage and schedule, and notify your healthcare team if the pain is not adequately controlled or if you experience any concerning symptoms.

Wound care: If an incision was made during the procedure, it is important to keep the wound clean and dry to prevent infection. Follow the specific wound care instructions provided by your healthcare team, such as keeping the incision site covered and avoiding activities that may put strain on the incision area.

Medication management: Take any prescribed medications as directed by your healthcare team. This may include medications to manage pain, prevent infection, or support your liver function. Inform

your healthcare team about any existing medications or supplements you are taking to ensure there are no potential interactions.

Follow-up appointments: Schedule and attend all follow-up appointments as recommended by your healthcare team. These appointments are essential for monitoring your progress, assessing treatment response, and addressing any concerns or questions you may have.

Physical activity and lifestyle modifications: Your healthcare team will provide guidance on when you can resume regular activities and exercise. It is important to gradually increase your activity level and follow any specific recommendations regarding physical activity. Depending on your condition and treatment plan, there may be certain lifestyle modifications to consider, such as dietary changes or limitations on alcohol consumption.

Emotional support: Going through any medical procedure can be emotionally challenging. Seek emotional support from your loved ones, join support groups, or consider speaking with a counselor or therapist to help manage any emotional stress or concerns that may arise during your recovery.

It is crucial to maintain open and regular communication with your healthcare team throughout your recovery process. They will provide you with personalized guidance, address any questions or concerns you may have, and monitor your progress to ensure a successful recovery.

2. Will I experience pain after TACE?

It is possible to experience pain or discomfort after undergoing Transarterial Chemoembolization (TACE), although the level of pain can vary from person to person. Here are some key points to consider regarding post-surgical pain:

Pain management: Your healthcare team will provide you with guidance on managing post-operative pain. This may involve the use of over-the-counter pain medications, prescription pain relievers, or other forms of pain management techniques. It is important to follow the prescribed dosage and schedule provided by your healthcare team.

Expected discomfort: It is normal to experience some degree of discomfort, soreness, or tenderness in the area where the procedure was performed. The level of discomfort can vary depending on individual factors, the extent of the procedure, and your overall health. The discomfort is typically temporary and should gradually improve as you recover.

Communication with your healthcare team: It is important to communicate any pain or discomfort you experience with your healthcare team. They can provide further guidance, adjust your pain management regimen if necessary, or investigate any potential complications that may be causing the pain.

Duration of pain: The duration of post-surgical pain can vary. In most cases, the pain and discomfort should start to subside within a few days to a couple of weeks after the procedure. However, it is important to note that individual recovery times may vary, and it is not uncommon to experience intermittent discomfort or mild pain during the healing process.

Signs of complications: While some level of pain is expected after the procedure, it is important to be aware of any signs of complications that may require immediate medical attention. Contact your healthcare team if you experience severe or worsening pain, excessive swelling, fever, bleeding, or any other concerning symptoms.

Your healthcare team will provide you with specific instructions and recommendations for managing post-operative pain. It is important to follow these instructions, take any prescribed medications as directed, and reach out to your healthcare team if you have any concerns or questions about your pain management or recovery process.

3. What pain medication can I take after TACE?

The specific pain medication recommended after Transarterial Chemoembolization (TACE) can vary depending on individual factors, the extent of the procedure, and your medical history. Your healthcare team will provide you with personalized instructions on pain management, including the specific pain medication to take. Here are some commonly used pain medications after TACE:

Nonsteroidal anti-inflammatory drugs (NSAIDs): Over-the-counter NSAIDs such as ibuprofen (Advil, Motrin) or naproxen (Aleve) are commonly used for mild to moderate pain relief. They help reduce pain and inflammation. However, it's important to consult with your healthcare team before taking NSAIDs, as they may have certain contraindications or interactions with other medications you are taking.

Acetaminophen: Acetaminophen (Tylenol) is another over-the-counter pain reliever that can be used for mild to moderate pain. It is generally well-tolerated but should be taken according to the recommended dosage.

Prescription opioids: In some cases, if the pain is more severe, your healthcare team may prescribe stronger pain medications, such as opioids (e.g., codeine, oxycodone, hydrocodone). These medications should be taken as directed by your healthcare provider, and it's important to be aware of the potential side effects and risks associated with opioids, including the risk of dependence and respiratory depression.

Local anesthetics: In certain situations, your healthcare team may use local anesthetics during the procedure, which can provide immediate pain relief in the treated area. The effect of local anesthetics may last for several hours after the procedure.

It is crucial to follow the instructions provided by your healthcare team regarding pain medication usage. They will consider your specific medical history, any allergies or contraindications, and tailor the pain management plan to your individual needs. Make sure to inform your healthcare team about any medications you are currently taking to avoid potential drug interactions.

If you have any questions or concerns about pain medication options after TACE, it is important to discuss them with your healthcare team. They can provide you with the most accurate and up-to-date information based on your specific situation and ensure you receive appropriate pain relief during your recovery.

4. What kind of care should I provide for the puncture site or incision?

Proper care of the puncture site or incision after Transarterial Chemoembolization (TACE) is crucial to prevent infection, promote healing, and minimize complications. Here are some general guidelines for caring for the puncture site or incision:

Follow instructions: Adhere to the specific post-operative instructions provided by your healthcare team regarding wound care. These instructions may vary depending on the size and location of the incision, as well as any additional procedures performed during the TACE.

Keep the area clean: Cleanliness is essential to prevent infection. Wash your hands thoroughly with soap and water before touching the puncture site or incision. Follow your healthcare team's instructions regarding cleaning the wound area, which may involve gentle cleansing with mild soap and water or a specific antiseptic solution. Avoid harsh scrubbing or using irritants on the wound.

Dressing changes: Your healthcare team will provide guidance on whether to keep the puncture site or incision covered with a sterile dressing or bandage. Follow their instructions regarding when and how to change the dressing. If the dressing becomes soiled, wet, or if there is any leakage, contact your healthcare team for advice on changing it.

Monitor for signs of infection: Keep a close eye on the puncture site or incision for any signs of infection, such as increasing redness, swelling, warmth, tenderness, pus, or worsening pain. Also, watch for any signs of systemic infection, such as fever, chills, or malaise. If you notice any concerning symptoms, promptly contact your healthcare team.

Avoid excessive movement or strain: Minimize activities that may strain or put undue pressure on the puncture site or incision. Follow your healthcare team's instructions on activity restrictions, lifting restrictions, and any specific precautions to prevent injury or disruption to the wound.

Avoid soaking or submerging the area: Until your healthcare team advises otherwise, avoid soaking the puncture site or incision in water, such as in bathtubs, pools, or hot tubs. This helps to reduce the risk of infection and promotes proper healing.

Follow a healthy lifestyle: A healthy lifestyle can support wound healing. Eat a balanced diet rich in nutrients, stay hydrated, and avoid smoking, as smoking can impair the healing process.

Attend follow-up appointments: It is important to attend all scheduled follow-up appointments with your healthcare team. They will assess the healing progress, remove any sutures or staples if necessary, and address any concerns or questions you may have.

Remember, these are general guidelines, and your specific wound care instructions may vary. Always consult your healthcare team for personalized advice and follow their recommendations closely to ensure optimal care for the puncture site or incision and promote a smooth recovery.

5. Are there any specific restrictions or limitations on activities following TACE?

Following Transarterial Chemoembolization (TACE), your healthcare team may provide specific instructions regarding restrictions or limitations on activities during your recovery period. These instructions can vary based on individual factors, the extent of the procedure, and your overall health. It is important to consult with your healthcare team for personalized guidance. However, here are some general considerations:

Rest and recovery: It is common to experience fatigue and decreased energy levels after TACE. Give yourself time to rest and allow your body to heal. Balance periods of activity with periods of rest to prevent excessive strain.

Activity restrictions: Your healthcare team may recommend avoiding strenuous activities, heavy lifting, or intense exercise for a certain period of time after the procedure. These restrictions are intended to minimize the risk of complications and promote proper healing. Follow their guidance regarding specific activities to avoid or modify.

Driving restrictions: Depending on the anesthesia or sedation used during the procedure, you may be advised to refrain from driving for a certain period of time after TACE. Anesthesia or sedation can affect coordination, judgment, and reflexes. It is important to follow your healthcare team's recommendations regarding driving restrictions.

Work considerations: The time needed to resume work activities can vary based on the nature of your job, your recovery progress, and any potential side effects experienced. Some individuals may be able to return to work relatively soon after TACE, while others may require a longer recovery period. Discuss with your healthcare team when it is safe for you to return to work and any accommodations that may be necessary.

Avoiding certain medications and substances: Your healthcare team may advise you to avoid certain medications or substances that can interfere with the healing process or interact with the

medications you are taking post-TACE. Follow their recommendations regarding the use of specific medications, alcohol consumption, and smoking.

Dietary considerations: While there may not be strict dietary restrictions following TACE, maintaining a healthy and balanced diet is generally beneficial for healing and overall well-being. Your healthcare team may provide specific dietary recommendations or guidelines based on your individual needs.

It is important to have open communication with your healthcare team regarding activity restrictions and any concerns or questions you may have. They can provide you with personalized guidance based on your specific situation and help you navigate your recovery period effectively.

6. How long do I need to stay lying down after the procedure?

The duration of lying down after Transarterial Chemoembolization (TACE) can vary depending on individual factors and the specific instructions provided by your healthcare team. In general, it is common to be advised to remain lying down for a few hours after the procedure to allow for proper recovery and minimize the risk of complications. However, the exact duration may vary based on the specific circumstances of your procedure.

Your healthcare team will provide you with clear instructions regarding the recommended duration of lying down after TACE. They may specify a specific timeframe, such as 2-4 hours, or provide more individualized guidance based on your condition. It is crucial to follow their instructions closely for optimal recovery.

During the period of lying down, it is important to keep the puncture site or incision area immobilized and avoid unnecessary movement or strain. Your healthcare team may provide a compression bandage or other forms of support to help stabilize the area and minimize the risk of bleeding or other complications.

It is worth noting that individual recovery times and post-procedure instructions can vary. After the designated period of lying down, your healthcare team may provide further guidance on when you can gradually resume normal activities. This may include instructions on gradually increasing mobility, resuming light activities, and any activity restrictions or precautions to be mindful of during the recovery period.

It is important to consult with your healthcare team for specific instructions regarding the duration of lying down after TACE. They will provide you with the most accurate and personalized guidance based on your individual procedure and condition.

7. Will I be admitted as an inpatient?

The decision to admit a patient as an inpatient after Transarterial Chemoembolization (TACE) depends on several factors, including the specific treatment protocol, the individual's overall health condition,

and the healthcare facility's policies. In some cases, TACE can be performed on an outpatient basis, meaning you may not need to stay overnight in the hospital. However, in other cases, an overnight or short-term hospital admission may be required.

The duration of hospitalization can vary. Some individuals may be discharged on the same day as the procedure, while others may require an overnight stay for observation and monitoring. The decision will be made by your healthcare team based on factors such as the complexity of the procedure, your response to the treatment, the need for close monitoring, and any potential complications that may arise.

Your healthcare team will discuss the expected length of your hospital stay and provide you with specific information regarding admission and discharge procedures. They will also provide instructions on post-procedure care, including any activity restrictions, wound care, and medication management.

It is important to communicate with your healthcare team about any concerns or questions you may have regarding your hospitalization. They will provide you with the necessary information and support to ensure a smooth transition from the procedure to post-procedure care.

8. What signs or symptoms should I watch for that may indicate a problem or complication?

After Transarterial Chemoembolization (TACE), it is important to monitor your condition closely for any signs or symptoms that may indicate a problem or complication. While complications are relatively rare, it is essential to be vigilant and seek medical attention if you experience any concerning symptoms. Here are some signs or symptoms that you should watch for:

Excessive bleeding: Monitor the puncture site or incision for any signs of excessive bleeding, such as continuous bleeding or bleeding that does not stop with gentle pressure. Notify your healthcare team immediately if you observe significant bleeding.

Infection: Look out for signs of infection, including increasing redness, swelling, warmth, tenderness, or drainage (pus) at the puncture site or incision. Also, watch for any systemic signs of infection, such as fever, chills, or malaise. If you suspect an infection, contact your healthcare team promptly.

Pain or discomfort: Some degree of pain or discomfort is expected after the procedure. However, if you experience severe or worsening pain that is not relieved by prescribed pain medication, it may indicate a problem. Contact your healthcare team for further evaluation.

Shortness of breath or chest pain: If you experience sudden or severe shortness of breath, chest pain, or difficulty breathing, it could be a sign of a serious complication. Seek immediate medical attention or call emergency services.

Nausea, vomiting, or abdominal pain: While mild gastrointestinal symptoms can occur after TACE, persistent or severe nausea, vomiting, or abdominal pain should be reported to your healthcare team.

Changes in mental status: If you notice any significant changes in mental status, such as confusion, disorientation, or difficulty speaking, it could indicate a neurological complication. Seek medical attention promptly.

Signs of liver dysfunction: TACE can affect liver function, so be vigilant for symptoms such as jaundice (yellowing of the skin or eyes), dark urine, pale stools, or unexplained fatigue. These may indicate liver problems and should be reported to your healthcare team.

Allergic reactions: Although rare, allergic reactions to medications or contrast agents used during TACE can occur. If you develop symptoms such as hives, rash, swelling, difficulty breathing, or a rapid heartbeat, seek immediate medical attention.

It is important to remember that these signs and symptoms are general guidelines, and your healthcare team will provide you with specific instructions on what to watch for and when to seek medical attention. Follow their guidance closely and do not hesitate to reach out to them if you have any concerns or questions about potential complications.

9. For how long will I typically need to stay in hospital after TACE?

The length of hospital stay following Transarterial Chemoembolization (TACE) can vary depending on several factors, including the specific treatment protocol, your individual response to the procedure, and your overall health condition. In some cases, TACE can be performed as an outpatient procedure, meaning you may be discharged on the same day. However, in other cases, a short-term hospital admission of one to several days may be necessary for monitoring and recovery.

Typically, the duration of hospital stay after TACE ranges from a few hours to one or two nights. The decision regarding the length of stay is made by your healthcare team based on factors such as the complexity of the procedure, your response to the treatment, the need for close monitoring, and any potential complications that may arise.

During your hospital stay, your healthcare team will monitor your vital signs, assess your pain level, and ensure that you are stable before considering discharge. They may also perform additional tests or imaging studies to evaluate the effectiveness of the treatment and monitor any potential complications.

It is important to note that every individual's situation is unique, and the length of hospital stay can vary. Your healthcare team will provide you with specific information regarding the expected duration of your hospitalization and communicate any changes or updates as necessary.

After discharge, you will receive instructions on post-procedure care, including wound care, medication management, activity restrictions, and follow-up appointments. It is important to follow these instructions closely to support your recovery and ensure the best possible outcome.

Keep in mind that the information provided here is a general guideline, and your actual hospital stay duration may differ. Your healthcare team will provide you with personalized guidance based on your specific case and condition.

10. Will I need any follow-up appointments?

Yes, after undergoing Transarterial Chemoembolization (TACE), you will likely need follow-up appointments with your healthcare team. These follow-up appointments are important for monitoring your progress, assessing treatment effectiveness, managing any potential side effects or complications, and making necessary adjustments to your ongoing care plan. The specific frequency and duration of follow-up appointments may vary based on individual factors and the recommendations of your healthcare team.

During these follow-up appointments, your healthcare team will typically perform various evaluations, which may include:

Imaging studies: Imaging tests such as CT scans, MRI scans, or angiograms may be conducted to assess the response of the treated area, monitor any residual tumor, and evaluate the overall condition of your liver.

Blood tests: Blood tests, including liver function tests and tumor marker tests, may be ordered to assess the functioning of your liver, monitor tumor markers, and detect any potential complications or changes in your health.

Physical examination: Your healthcare provider will conduct a physical examination to assess your general health, evaluate the healing of the puncture site or incision, and check for any signs of complications or recurrence.

Discussion and consultation: You will have an opportunity to discuss your progress, any concerns or symptoms you may be experiencing, and ask any questions you have regarding your recovery, further treatment options, and long-term management.

The exact schedule of follow-up appointments will be determined by your healthcare team based on your individual needs and treatment plan. It is essential to attend all scheduled follow-up appointments and communicate any changes or concerns with your healthcare team between appointments. This ensures that your condition is closely monitored, and any necessary interventions or adjustments can be made in a timely manner.

Remember to ask your healthcare team about the specific follow-up plan and any recommended self-care measures to optimize your recovery and long-term outcomes.

11. How often does TACE need to be repeated?

The frequency of Transarterial Chemoembolization (TACE) treatments and the need for repetition depend on several factors, including the underlying condition being treated, the response to the initial treatment, the extent and aggressiveness of the disease, and the overall treatment plan determined by your healthcare team. TACE is often performed in multiple sessions, with intervals between treatments to allow for recovery and assessment of treatment effectiveness.

In some cases, a single session of TACE may be sufficient to achieve the desired treatment goals, such as tumor shrinkage or control of symptoms. However, in many instances, TACE is performed as part of a planned treatment strategy involving multiple sessions over a period of time.

The specific interval between TACE sessions can vary, and your healthcare team will determine the optimal timing based on your individual circumstances. This may range from several weeks to a few months, allowing time for the treated area to heal, for the evaluation of treatment response, and for the planning of subsequent sessions.

The number of TACE sessions required can vary significantly. It depends on factors such as the size and number of tumors, their location within the liver, the overall response to treatment, and the individual treatment goals. It is not uncommon for patients to undergo several TACE sessions spaced apart over several months to achieve the desired treatment outcomes.

Ultimately, the decision regarding the need for repeat TACE treatments and the schedule for subsequent sessions will be determined by your healthcare team. They will evaluate your response to treatment, consider the progression of the disease, and make recommendations based on the best available evidence and guidelines for your specific condition.

It is important to have open and regular communication with your healthcare team throughout your treatment journey. They will provide you with specific guidance on the frequency and timing of TACE sessions, monitor your progress, and make adjustments to your treatment plan as necessary to optimize your outcomes.
