# Supplementary material for: Feasibility of GPT-3 and GPT-4 for in-Depth Patient Education Prior to Interventional Radiological Procedures: A Comparative Analysis
Source: Cardiovasc Intervent Radiol. 2023 Oct 23;47(2):245–50. doi: 10.1007/s00270-023-03563-2 (PMC10844465; doi:10.1007/s00270-023-03563-2)
Supplement: Supplementary file 4 — Supplementary file4 (PDF 280 kb) [file 270_2023_3563_MOESM4_ESM.pdf]

GPT-4 Port

General Questions:

1. What is a port?

A port, or a "port-a-cath" or "implanted port", is a small medical appliance that is installed beneath the skin. It's a type of central venous catheter (CVC).

A port has two primary components: a round metallic or plastic "portal" (also sometimes called a reservoir), and a rubber-like top known as the septum. The portal is implanted under the skin in the upper chest or in the arm, and the catheter connected to the portal is threaded into a large vein.

The main function of a port is to ease the process of repeated injections or infusions (like chemotherapy drugs, blood transfusions, antibiotics), and for the withdrawal of blood for testing. To access the port, medical personnel insert a special needle (often called a Huber needle) through the skin and the septum to reach the reservoir.

Because it is entirely beneath the skin, a port carries a lower risk of infection compared to other types of central lines and can be left in place for long periods of time, making it a good option for patients who require regular medication infusions or blood draws.

2. Why do I need a port?

A port is typically recommended for patients who require long-term intravenous (IV) treatments or frequent blood draws. Here are a few reasons why you might need a port:

Chemotherapy: If you have cancer and need chemotherapy, the drugs are often delivered through a port. Chemotherapy drugs can be harsh on the smaller veins in your arms, so delivering them through a port into a larger vein is often a better choice.

Long-term antibiotic therapy: If you have a severe infection and require antibiotics intravenously for an extended period, a port can be a convenient way to deliver the medication.

Nutritional therapy: Some people who cannot eat food normally might require total parenteral nutrition (TPN), which is delivered through a port.

Frequent blood draws: If you have a condition that requires regular blood testing, a port might be a more comfortable solution than frequent needle sticks in your peripheral veins.

Blood transfusions: People who require regular blood transfusions, such as those with certain types of anemia, might also benefit from a port.

However, the use of a port does come with some risks, including infection, blood clots, and potential issues with the port's placement. Therefore, the decision to use a port is made after a thorough discussion with your healthcare provider, considering your personal medical situation, the potential benefits, and the risks involved.

3. What alternatives do I have to a port?

There are several alternatives to an implanted port for providing intravenous (IV) access, depending on the specifics of your medical situation, how long you'll need treatment, and your personal preferences. Here are a few common alternatives:

Peripheral IV Line: This is the most common type of IV access. A catheter is inserted into a small vein in your arm or hand. However, these are usually only suitable for short-term use (hours to days) because they can easily become blocked or infected.

Peripherally Inserted Central Catheter (PICC): A PICC line is a long, thin tube that's inserted through a vein in your arm and guided to a larger vein near your heart. A PICC line can be left in place for weeks to months and is a good choice for long-term treatments.

Central Venous Catheter (CVC): A CVC is similar to a PICC line, but it's typically inserted into a large vein in your neck, chest, or groin. Like a PICC line, a CVC can be left in place for a long time.

Tunneled Catheter: This type of catheter is placed under your skin and then tunneled to a large vein. One end stays outside your body. This type of catheter is useful for long-term treatments and has less risk of infection compared to PICC lines and CVCs because the tunnel under the skin provides an extra barrier against bacteria.

Each of these options has pros and cons, and the best choice depends on factors like the type and duration of treatment, the condition of your veins, your overall health status, and your lifestyle. Your healthcare provider can help you understand which option is best for your specific situation.

4. What are the advantages of having a port?

Having a port has several advantages, particularly for people who require long-term or frequent intravenous treatments. Here are a few key advantages:

Comfort: Once a port is implanted and healed, it can be more comfortable than other forms of IV access because it eliminates the need for frequent needle sticks in the peripheral veins.

Convenience: A port allows for easy access to your bloodstream, which can simplify the process of receiving medications, especially for treatments that are administered frequently or over a long period of time.

Reduced Infection Risk: Because the port is entirely under the skin when not in use, it has a lower risk of infection compared to catheters that exit the skin, such as PICC lines or external central venous catheters.

Long-term Use: A port can remain in place for months or even years if necessary, making it a good choice for people who require long-term therapy.

Lifestyle: Once a port is implanted, you can bathe and swim without worrying about the entry site. It's also less noticeable than external devices, which may be a significant advantage for some people in terms of body image or self-confidence.

Preservation of Peripheral Veins: Chemotherapy and some other medications can cause irritation or damage to smaller peripheral veins. Administering these medications through a port can help to preserve these veins.

Remember, while there are many advantages to having a port, it's important to discuss your personal situation and needs with your healthcare provider to make the best decision for your care.

5. What medication can be administered through a port?

A wide variety of medications can be administered through a port. These include:

Chemotherapy: Chemotherapy drugs are often administered through a port, especially if the treatment regimen requires multiple cycles over weeks or months. This is because these drugs can be harsh on smaller veins.

Antibiotics: Long-term intravenous antibiotic therapy can also be administered through a port, particularly for severe infections that are not responding to oral antibiotics.

Pain Medications: Some patients with chronic pain conditions may receive pain medications through a port.

Biological Therapies: This includes a wide range of treatments for conditions like rheumatoid arthritis or Crohn's disease that involve the administration of proteins or other biological agents.

Total Parenteral Nutrition (TPN): TPN is a method of feeding that bypasses the gastrointestinal tract for those who are unable to or should not receive their nutrition orally or enterally (via feeding tubes). It is administered directly into the bloodstream through a port.

Blood Products: This includes blood transfusions, platelet infusions, and plasma infusions.

Fluids and Electrolytes: In certain situations, you may require intravenous hydration or electrolyte repletion, which can be delivered through a port.

Certain Medications for Heart Conditions: Medications such as inotropic drugs for heart failure can also be given via a port.

Your healthcare provider can provide information specific to your treatment regimen and whether your medications can be safely and effectively delivered through a port. The port provides direct access to the central circulation, so most drugs that can be given intravenously can be administered through a port.

6. How is the medication administered?

Administering medications through a port involves several steps, often performed by a healthcare professional:

Accessing the Port: First, a special type of needle (commonly called a Huber needle) is inserted through the skin and into the septum (the rubber-like top) of the port. This is usually done using a sterile technique to minimize the risk of infection. An anesthetic cream can be applied to the skin over the port before the needle insertion to numb the area if needed.

Checking the Port: Once the needle is in place, the healthcare provider will draw back on the syringe attached to the needle. If blood returns easily, it confirms that the port and catheter are functioning correctly and are ready for use.

Flushing the Port: Before and after administering medication, the port is typically flushed with a heparin or saline solution. This helps ensure that the catheter remains unblocked and that all the medication has been delivered.

Administering Medication: The medication can be given in a few different ways, depending on the type and duration of the medication. It might be injected directly into the port, infused over a set period of time, or connected to an infusion pump for continuous administration.

Removing the Needle: After the medication has been delivered and the port flushed, the needle is removed and a bandage is applied to the site. If the port will be accessed regularly over a period of days (for example, during a hospital stay), the needle might be left in place and securely covered with a dressing.

Patients and their caregivers can be trained to care for the port and administer medications at home, but this depends on the type of treatment, the patient's general health, and their comfort level with the process. Always follow the specific instructions provided by your healthcare team.

7. What does a port look like?

A port, or port-a-cath, is a small medical device that consists of two main parts: the portal (or reservoir) and the catheter.

The Portal/Reservoir: The portal is a small, round or oval device made of a durable material like plastic or metal. It's usually about the size of a quarter or slightly larger. The top of the portal is covered with a self-sealing silicone septum, which is what allows needles to be inserted and removed without damaging the device.

The Catheter: Attached to the portal is a thin, flexible tube called a catheter. The length of the catheter can vary depending on where the portal is placed and where the catheter needs to end in the vein.

The entire port device is fully implanted beneath the skin, usually in the chest area or the upper arm. Once implanted, the only visible sign of the port might be a small bump under the skin where the portal is located, and possibly a faint line under the skin where the catheter runs. However, for many people, the port is not noticeably visible at all.

The specific appearance of a port can vary somewhat depending on the manufacturer and the exact model of the device. If you're considering having a port implanted, your healthcare provider can show you what the device will look like and explain more about how it will be placed and used.

8. Can blood be drawn through the port as well?

Yes, blood can be drawn through a port. This is one of the reasons a port can be very useful for patients who need frequent blood tests.

The process is somewhat similar to administering medication. A healthcare provider will insert a special needle (often a Huber needle) through the skin and into the port. They can then attach a syringe or vacutainer to draw blood. After the blood draw, the port is usually flushed with a heparin or saline solution to keep it open and functioning correctly.

Just like with medication administration, the needle insertion and blood draw should be done using sterile technique to minimize the risk of infection. Patients can be trained to care for their port at home, but drawing blood usually requires a healthcare professional

9. Where will a port be implanted?

A port is usually implanted in the upper chest, just below the collarbone, with the catheter running into the jugular vein and then down to the heart. This location allows for easy access, and the port can be comfortably and discreetly worn under clothing.

Alternatively, a port can be implanted in the upper arm, with the catheter running into one of the large veins of the arm towards the heart. This might be chosen for cosmetic reasons, or if there's a medical reason that prevents implantation in the chest.

The placement is usually on the side opposite your dominant hand, but it can depend on your personal circumstances and your doctor's recommendations.

The exact placement can vary depending on factors such as the patient's body habitus, the condition of their veins, and the specific type of port being used. The procedure to implant a port is generally done under local anesthesia, often with sedation, and may involve imaging technology like ultrasound or fluoroscopy to guide the placement of the catheter in the vein.

10. How long does a port remain in place?

A port is designed to be a long-term solution for accessing your bloodstream. It can stay in place for several months to several years, depending on your medical needs.

The duration for which a port remains in place depends on a few factors:

Your Treatment Plan: If you are receiving a treatment like chemotherapy, the port will likely stay in place for the duration of your treatment. Some treatment regimens last several months, while others may last a few years.

Your Medical Condition: Some chronic conditions might require long-term or even lifelong access to the bloodstream. In these cases, a port can remain in place indefinitely, as long as it's functioning well and not causing any complications.

The Condition of the Port: Over time, a port can sometimes develop complications such as infections, blockages, or mechanical issues. If this occurs, it may need to be removed or replaced.

Maintenance and Care: Proper care of the port, including regular flushing and cleanliness when accessing, can help prolong its usability and prevent complications.

If you no longer need it for treatment, and it's functioning well without complications, your healthcare provider may leave the port in place in case it's needed in the future, particularly if there's a likelihood of recurrence of your condition. However, in some cases, they may choose to remove it.

As always, the specifics of how long a port will stay in place will be determined by your individual circumstances and your healthcare provider's recommendations.

Preparation and procedure:

1. How should I prepare for the port implantation?

Preparing for port implantation involves several steps, and your healthcare team will give you specific instructions based on your personal health condition and the exact procedure you will be undergoing. Here are some general guidelines:

Medical Evaluation: Before the procedure, your doctor will conduct a thorough medical evaluation. This might include blood tests, imaging studies, or other tests to ensure that you're healthy enough for the procedure and to plan the best approach for the port implantation.

Medications: Your doctor will review your current medications and may instruct you to stop taking certain drugs before the procedure. For instance, you might need to stop taking blood thinners temporarily to reduce the risk of bleeding. However, do not stop taking any medication without your doctor's approval.

Fasting: Typically, you'll be asked to fast (not eat or drink anything) for a certain period before the procedure. The exact duration can vary, but it's often at least 6-8 hours.

Clothing: Wear comfortable, loose-fitting clothing to the hospital. You'll likely need to change into a hospital gown for the procedure.

Personal Items: You might want to bring personal items like a book or music to help relax before the procedure. However, keep in mind that valuables should generally be left at home.

Transportation: Because you'll likely receive sedation for the procedure, you'll need to arrange for someone to drive you home afterward.

Questions and Concerns: Don't hesitate to ask your healthcare provider about any questions or concerns you have about the procedure. Knowing what to expect can help reduce anxiety and ensure that you're prepared for the implantation.

Always follow your healthcare provider's specific instructions for preparation. Your preparation might be slightly different based on your personal medical history and the specific procedure being done.

2. Do I need someone to accompany me on the day of the surgery?

Yes, you will likely need to have someone accompany you on the day of the surgery. This is because you will be given sedation or anesthesia during the procedure and will not be able to drive yourself home afterward due to the residual effects of these medications.

Even if you're not feeling overly groggy, reaction times and cognitive function can be impacted for several hours after sedation or anesthesia. Therefore, it's important to have someone else available to drive you home to ensure your safety.

Moreover, having a friend or family member accompany you can be comforting and helpful. They can assist you in remembering post-procedure instructions given by your healthcare team and provide support during your recovery. Always check with your healthcare provider for specific instructions and recommendations related to your procedure.

3. Am I allowed to eat and drink before the surgery?

Typically, you're asked to fast before surgery, which means not eating or drinking anything, usually after midnight the night before the procedure. This includes avoiding water, chewing gum, and even hard candies. The main reason for this is to prevent the risk of aspiration (inhaling food or liquid into your lungs) while under anesthesia.

However, the specific instructions can vary depending on factors like the time of your procedure, your overall health condition, and the type of anesthesia being used. Certain medications may be allowed with a small sip of water, as instructed by your healthcare provider.

These are general guidelines, and your doctor or anesthesiologist will provide you with specific instructions based on your personal medical situation. Always follow these instructions closely. If you accidentally eat or drink too close to the time of your procedure, be sure to inform your healthcare provider as it could potentially impact your surgery.

4. Can I take my medications before the surgery?

Whether or not you can take your medications before the surgery depends on what medications you're taking and the specific instructions from your healthcare team.

Some medications, especially those related to heart conditions, blood pressure, or asthma, are often continued even on the day of surgery with a small sip of water. Other medications, such as blood thinners, certain diabetic medications, or non-steroidal anti-inflammatory drugs (NSAIDs), may need to be adjusted or temporarily stopped before the procedure to reduce the risk of complications.

In the days leading up to your surgery, your doctor or a member of your healthcare team will review your current medications with you, including prescription drugs, over-the-counter medications, and any dietary supplements you're taking. They will provide specific instructions about which medications you should take, which you should skip, and the appropriate timing.

Remember, it's important to follow these instructions carefully and to communicate openly with your healthcare team about all the medications and supplements you're taking. If you have any doubts or questions, don't hesitate to ask them for clarification.

5. Which medications am I not allowed to take before the surgery?

Certain types of medications may need to be stopped or adjusted before surgery because they can increase the risk of complications. However, the specific medications you'll need to avoid will depend on your personal health history and the medications you're currently taking. Always discuss this with your healthcare provider to receive personalized instructions.

Here are some types of medications that often need to be managed prior to surgery:

Blood Thinners: Medications that thin the blood, such as warfarin (Coumadin), clopidogrel (Plavix), and certain over-the-counter medications like aspirin or ibuprofen, can increase the risk of bleeding during and after surgery. Your doctor may instruct you to stop these medications several days before the procedure.

Diabetes Medications: Insulin and other diabetes medications may need to be adjusted to prevent low blood sugar (hypoglycemia) during the surgery, especially since you'll be fasting.

Immunosuppressants: If you're taking drugs that suppress your immune system, such as for autoimmune diseases or after organ transplantation, you may need to adjust these medications. These drugs can affect your body's ability to heal and fight off infection after surgery.

Herbal Supplements: Certain dietary and herbal supplements can affect surgery outcomes. For example, supplements like ginkgo biloba, ginseng, and garlic can increase bleeding risk, while others like St. John's Wort can interfere with anesthesia.

Remember, never stop or adjust your medications without first discussing it with your healthcare provider. They'll provide detailed instructions on how to manage your medications leading up to the procedure. If you're unsure about any medication, it's always better to ask.

6. How does the surgery proceed?

The process for implanting a port can vary based on several factors, including the specific type of port being used and the patient's individual health status. However, here is a general outline of what you might expect:

Preparation: Before the surgery begins, you will be given a hospital gown to change into. An IV line will be started in your hand or arm so that medications can be given. Monitors will be attached to your body to track your vital signs during the procedure.

Anesthesia/Sedation: You will likely receive sedation to help you relax, and local anesthesia will be used to numb the area where the port will be implanted. In some cases, general anesthesia may be used instead, so you are asleep during the procedure.

Incision and Pocket Creation: The surgeon will make a small incision (usually about 1-2 inches long) in your skin. Just beneath this incision, the surgeon will create a "pocket" in the fatty tissue under your skin. This is where the port will be placed.

Catheter Placement: A second small incision is made near a large vein (typically in your neck or chest), and the catheter (the long, thin tube connected to the port) is inserted into this vein. The other end of the catheter is threaded through a tunnel under your skin and connected to the port in the pocket. The placement of the catheter may be guided by ultrasound or fluoroscopy (a type of real-time X-ray) to ensure it's in the correct position in the vein.

Securing the Port: The port is then secured in place and the incisions are closed, typically using stitches, surgical staples, or adhesive strips. A dressing is applied to the area.

Post-Procedure Imaging: After the surgery, an X-ray or other imaging test may be done to confirm the catheter is in the right position.

Recovery: You will be monitored in the recovery area until the effects of the anesthesia or sedation have worn off. Instructions will be given for how to care for your incisions, and you will likely be prescribed or recommended pain medication to manage any post-procedure discomfort.

The entire procedure usually takes between 1 to 2 hours. Remember, this is a general guide and the exact process can vary. Always discuss the specifics of the procedure with your healthcare team.

7. Will I be awake during the surgery?

Whether you are awake or asleep during the surgery can depend on the specific type of anesthesia that is used.

In many cases, port implantation procedures are performed under local anesthesia and conscious sedation. Local anesthesia involves injecting medication to numb the area where the surgery will be performed so you don't feel pain. Conscious sedation, also known as "twilight" anesthesia, involves giving medication that helps you relax or lightly sleep, but doesn't fully put you under.

While under conscious sedation, you may have limited awareness of your surroundings, but generally, you will be relaxed and comfortable, and might not remember much about the procedure afterwards.

In some situations, general anesthesia, which puts you into a deep sleep, might be used instead. This is less common for port placements but might be used if you are having other procedures at the same time or if it's medically necessary.

The choice of anesthesia will be determined by your healthcare provider, taking into consideration your overall health, the specifics of your case, and your preferences. Your healthcare team will discuss this with you in detail before your procedure, and you should feel free to ask them any questions or express any concerns you may have.

8. Where will the port be implanted?

The specific location of the port implantation can vary based on individual factors such as body habitus, intended use, and the preference of the healthcare provider. However, the most common sites for port implantation are in the chest or upper arm.

In the chest, the port is typically placed just below the collarbone, often on the right side. This location provides easy access to the large veins that lead directly into the heart, allowing for effective delivery of medication.

If the port is placed in the upper arm, it's typically on the inner side, just above the elbow. This is less common than chest placement, but may be preferred in some situations.

In both cases, the port is placed under the skin in a 'pocket' created by the surgeon. A catheter is attached to the port and threaded into a nearby vein. The placement of the catheter is often guided by ultrasound or fluoroscopy to ensure accurate positioning.

Remember, the exact location can depend on various factors, and your healthcare provider will discuss this with you before the procedure.

9. Will the port be implanted on the left or right side?

The choice of whether to implant the port on the left or right side of the body depends on several factors, including:

The Patient's Condition: If there is a medical condition or surgical history that makes access to the veins on one side of the body difficult or impossible, the other side will be chosen.

The Vein's Condition: The healthcare provider will consider the condition and size of the patient's veins. If the veins on one side are not suitable for port placement, the port may be placed on the other side.

The Patient's Dominant Hand: Sometimes, the non-dominant side is preferred to minimize the risk of injury to the port.

Concurrent Medical Devices: If the patient already has a device implanted on one side (such as a pacemaker), the port may be placed on the other side.

In many cases, the right side is often chosen for port placement because the route to the central veins is more direct on that side. However, it is ultimately a decision made by the healthcare provider based on a careful evaluation of the patient's medical history, physical condition, and treatment needs.

10. How long does the surgery take?

The length of the surgery for port implantation can vary depending on several factors, including the specific technique used by the surgeon and the patient's individual circumstances. However, the procedure generally takes between 1 to 2 hours.

This includes time for the preparation (such as administering anesthesia and positioning the patient), the actual surgery to implant the port and catheter, and immediate post-procedure care like applying dressings and monitoring the patient's initial recovery.

Remember, this is a general estimate. Your healthcare provider will be able to give you a more accurate estimate based on your specific situation. It's also important to remember that preparation before the surgery and recovery time after the surgery will add to the overall time you spend in the hospital or surgery center.

11. Is the surgery painful?

The surgery to implant a port is typically not painful because anesthesia is used to prevent discomfort during the procedure.

Before the surgery starts, you'll likely receive a combination of local anesthesia and sedation. The local anesthesia numbs the area where the surgeon will be working, so you won't feel any pain during the procedure. The sedation can help you relax or even sleep lightly throughout the procedure, but you typically won't be fully unconscious as with general anesthesia.

You may feel some pressure or tugging sensations during the procedure, but you should not feel any sharp pain. If you do, it's important to let your healthcare team know so they can adjust your medications as necessary.

After the procedure, you may have some discomfort or tenderness at the site of the port and the catheter. This is typically managed with over-the-counter pain medications or a prescription from your doctor, if necessary. The discomfort usually improves over the first few days after the procedure.

Remember, everyone's experience with pain and discomfort is different. It's important to communicate openly with your healthcare team about what you're feeling both during and after the procedure, so they can help manage any discomfort effectively.

12. Will I be admitted as an inpatient?

Port implantation procedures are often performed on an outpatient basis, which means that you typically don't need to be admitted to the hospital and can go home on the same day as the surgery. You will need to stay for a while after the procedure for observation to ensure there are no immediate complications and that you have recovered from the anesthesia.

However, there are some situations where an overnight stay or inpatient admission might be necessary, such as if there are complications during the procedure, if you have other serious health conditions that need to be managed, or if you're already an inpatient for other reasons.

Keep in mind that these are general guidelines and the exact approach can vary based on factors like your specific health situation, the hospital's policies, and your doctor's recommendations. Be sure to discuss this with your healthcare provider to understand what to expect in your case.

Risks and Complications:

1. How safe is the procedure?

The procedure to implant a port is generally considered safe and is commonly performed. However, like any medical procedure, it carries some degree of risk. Complications are relatively rare, but can include:

Infection: This is a risk with any surgical procedure. The surgical site or the port itself can become infected. This is usually preventable with proper surgical technique and aftercare.

Bleeding or Hematoma: As with any invasive procedure, there is a risk of bleeding during or after the surgery. Hematomas (collections of blood) can also form at the surgical site.

Thrombosis or Embolism: These are blood clots that can form in or travel through the blood vessels. If a clot forms in the catheter or the vein in which the catheter is placed, it can lead to blockages and complications.

Air Embolism: This is a very rare complication where air gets into the venous system. This is typically prevented by ensuring that the system is free of air before the catheter is connected.

Misplacement of the Catheter: There is a risk that the catheter can be placed incorrectly, potentially leading to complications. This is minimized by using imaging guidance during the placement.

Damage to Surrounding Structures: There is a small risk of damage to nearby structures during the surgery, such as the lungs (which could potentially cause a pneumothorax, or collapsed lung) or nearby nerves.

Port or Catheter Malfunction: Over time, the port or catheter may stop working correctly, potentially requiring additional procedures to fix or replace the device.

Skin Breakdown: In some cases, the skin over the port may become thin or breakdown.

The healthcare team will take steps to minimize these risks, and the benefits of having the port (such as improved medication delivery and reduced need for repeated needle sticks) often outweigh the potential risks for patients who require long-term intravenous therapies.

It's also important to discuss your personal risk factors and any concerns you may have with your healthcare provider before the procedure. They can provide you with information tailored to your specific health situation.

2. What complications can occur during the procedure?

While the procedure to implant a port is generally considered safe, like any surgical procedure, there are potential complications that can occur during the operation. These include:

Bleeding: This can occur at the surgical site, although severe bleeding is rare.

Infection: There's a risk of infection anytime the skin is broken, including during surgery.

Misplacement of the Catheter: There is a risk that the catheter can be placed incorrectly. This is typically minimized by using imaging guidance during the placement.

Pneumothorax: This is a condition where air enters the space around the lungs, causing the lung to collapse. This is a rare complication that can occur if the lung is accidentally punctured during the procedure.

Arrhythmias: Rarely, manipulation of the catheter within the large central veins can irritate the heart and cause abnormal heart rhythms.

Air Embolism: This is a rare complication where air gets into the venous system, which can be life-threatening if not promptly treated. This is typically prevented by ensuring that the system is free of air before the catheter is connected.

Vascular injury: Damage to blood vessels can occur, although this is rare.

It's important to note that these complications are relatively rare, and the healthcare team performing the procedure will take a variety of precautions to minimize these risks. It's also crucial to discuss any concerns you may have with your healthcare provider before the procedure. They will be able to give you a thorough understanding of the risks based on your personal health situation.

3. Are there any specific factors that increase my risk of complications?

Yes, there are certain factors that can increase the risk of complications during or after a port implantation procedure. These can include:

Pre-existing medical conditions: Certain conditions, such as diabetes, obesity, immune deficiencies, or conditions that affect blood clotting can increase the risk of complications like infection or bleeding.

Poor nutritional status: Malnutrition can delay wound healing and increase the risk of infection.

Current medications: Some medications, particularly blood thinners, can increase the risk of bleeding during and after the procedure. Immunosuppressive drugs can increase the risk of infection.

Smoking: Smoking can delay wound healing and increase the risk of complications.

Previous surgeries or radiation therapy in the implantation area: These can cause scarring or changes in the tissue that may make the procedure more challenging and increase the risk of complications.

Age: Older age can increase the risk of complications, likely due to the presence of other underlying health conditions.

Remember, your healthcare provider will discuss these risks with you in detail prior to the procedure, and they will take all necessary steps to minimize potential complications. This can include adjusting medications, giving advice on smoking cessation, and providing guidelines on managing existing medical conditions. It's important to discuss your health history thoroughly with your healthcare provider so they can assess your individual risk and plan your care accordingly.

4. Can I die during the operation?

While it's important to note that all surgical procedures carry some risk, death during a port implantation procedure is extremely rare. This procedure is generally considered safe and is commonly performed. The complications that can occur during the procedure, such as bleeding or infection, are typically manageable and not life-threatening when treated appropriately.

The healthcare team performing the procedure takes several precautions to minimize the risk of complications, such as using sterile techniques to prevent infection and monitoring the patient closely during the procedure to manage any issues that arise immediately.

Severe complications that could potentially be life-threatening, such as a severe allergic reaction to anesthesia or a significant pneumothorax, are exceedingly rare. Additionally, these complications would be rapidly identified and treated by the medical team.

In summary, while there is always some risk associated with any surgical procedure, the risk of death during a port implantation procedure is extremely low. It's important to discuss any concerns you may have with your healthcare provider, who can give you a more accurate assessment of risk based on your personal health situation.

5. Can any complications occur after the procedure?

Yes, like any surgical procedure, complications can arise after port implantation. These are generally rare, but can include:

Infection: This can occur at the surgical site or involve the port or catheter itself. This can happen immediately after surgery or weeks to months later.

Bleeding or Hematoma: As with any invasive procedure, there is a risk of bleeding at the surgical site. Hematomas (collections of blood) can also form at the surgical site.

Thrombosis or Embolism: These are blood clots that can form in or travel through the blood vessels. If a clot forms in the catheter or the vein in which the catheter is placed, it can lead to blockages and complications.

Catheter Displacement: Over time, the catheter can move or become dislodged, which may require additional procedures to reposition or replace it.

Port or Catheter Malfunction: Over time, the port or catheter may stop working correctly, potentially requiring additional procedures to fix or replace the device.

Skin Breakdown: In some cases, the skin over the port may become thin or breakdown.

Regular care and maintenance of the port and catheter can help prevent many of these complications. Your healthcare provider will provide specific instructions on how to care for your port and when to seek medical attention.

Remember, your healthcare provider will discuss these risks with you in detail prior to the procedure, and they will take all necessary steps to minimize potential complications. It's important to discuss any concerns you may have with your healthcare provider so they can assess your individual risk and plan your care accordingly.

6. What are typical side effects of the procedure?

After the port implantation procedure, you may experience some common side effects. These are typically not serious and should improve within a few days to a week. They include:

Pain or Discomfort: You may experience some pain, discomfort, or tenderness at the site where the port was implanted. This should improve over time and can usually be managed with over-the-counter pain medications or a prescription from your doctor if necessary.

Swelling and Bruising: Some swelling and bruising around the surgical site is common after the procedure.

Fatigue: You may feel tired or weak after the procedure, particularly if you were given sedation. This should improve over the first day or two.

Mild Bleeding: Some mild bleeding or oozing from the surgical site can occur in the first day or two after the procedure.

Remember, these side effects should improve over time. If they worsen, or if you experience severe pain, significant bleeding, fever, redness or swelling at the port site, or difficulty breathing, you should seek medical attention promptly. These could be signs of complications such as infection, significant bleeding, or a pneumothorax (collapsed lung).

It's important to discuss any concerns or questions you have with your healthcare provider to ensure that you understand what to expect after the procedure. Your healthcare provider can provide you with detailed instructions on how to care for your port and the surgical site after the procedure, as well as what side effects to expect and when to seek medical attention.

7. Will I lose blood during the procedure?

Port implantation, like any surgical procedure, involves some level of blood loss, but it is typically minimal and does not have significant health impacts for most patients.

The procedure is performed using careful surgical techniques and tools designed to minimize blood loss. For example, the surgeon makes a small incision in the skin to insert the port and uses cauterization or other techniques to control any bleeding.

If you have a condition that affects your blood's ability to clot, or if you are taking medications that thin your blood, you may be at a higher risk for bleeding during the procedure. Your healthcare provider will review your medical history and current medications before the procedure to manage these risks.

In rare cases, more significant bleeding may occur, which could require additional interventions to control. However, the medical team performing the procedure is equipped to manage such complications if they arise.

Remember, it's important to discuss any concerns you have with your healthcare provider before the procedure. They can provide you with more specific information based on your individual health situation and the specifics of the planned procedure.

8. What structures can be injured during the procedure?

While port implantation is generally a safe procedure, as with any surgical procedure, there's a risk of injury to the surrounding structures. These risks are generally low, but could include:

Blood Vessels: The procedure involves inserting a catheter into a vein, usually the subclavian vein or jugular vein. There's a small risk of injury to these vessels during the procedure.

Lungs: The port is typically implanted in the chest, near the lungs. There's a small risk of a pneumothorax, or collapsed lung, if a lung is accidentally punctured during the procedure.

Nerves: There are various nerves in the chest area that could potentially be injured during the procedure, although this is rare.

Heart: Rarely, if the catheter is inserted too far, it can irritate the heart and cause irregular heart rhythms.

Skin and Subcutaneous Tissue: Infection, hematoma (a collection of blood outside of a blood vessel), or seroma (a pocket of clear serous fluid) can occur in the skin and tissues where the port is implanted.

Your healthcare provider will use imaging guidance during the procedure to minimize the risk of injury to these structures. In addition, your medical team will closely monitor you during and after the procedure to quickly identify and address any complications that may arise.

Please note that these are potential risks and are relatively rare. It's important to discuss any concerns you may have with your healthcare provider, who can provide you with more detailed information based on your individual health circumstances.

9. What happens in a pneumothorax?

A pneumothorax, also known as a collapsed lung, is a condition that occurs when air leaks into the space between your lung and chest wall. This air pushes on the outside of your lung and causes it to collapse.

Pneumothorax can occur as a result of a chest injury, lung disease, or certain medical procedures, including the insertion of a central venous catheter like a port. However, it's important to note that this is a rare complication of port placement.

The symptoms of a pneumothorax can vary depending on the size and cause of the air leak. They can include sudden chest pain on the side of the collapsed lung and shortness of breath. In severe cases, it can cause a rapid heart rate, rapid breathing, chest tightness, fatigue, and low blood pressure.

If a pneumothorax occurs during a procedure, it is typically identified quickly. The management of a pneumothorax depends on its size and severity. A small pneumothorax may resolve on its own over time, but larger or more severe pneumothoraces often require treatment to remove the air from the chest cavity. This is typically done by inserting a needle or a chest tube into the space between the lung and the chest wall, allowing the air to escape and the lung to re-expand.

Even though a pneumothorax is a serious condition, when it's identified and treated promptly, most people recover fully. Your healthcare provider will closely monitor you during and after the procedure to quickly identify and manage any complications that may arise.

10. How is a pneumothorax treated?

The treatment for a pneumothorax, or collapsed lung, largely depends on the severity of the condition.

Observation: If the pneumothorax is small and you're not experiencing severe symptoms, doctors may choose to simply monitor your condition. A small pneumothorax may heal on its own over time as the air is naturally reabsorbed from the pleural space. During this time, you might be asked to avoid flying, high altitudes, and scuba diving.

Needle Aspiration or Chest Tube Placement: For a larger pneumothorax or in cases where symptoms are more severe, a healthcare provider may use a needle or a small tube (chest tube) to remove the excess air from the space between the lungs and chest wall (pleural space), allowing the lung to re-expand. The needle or chest tube is usually inserted between the ribs into the pleural space, and the air is suctioned out.

Surgery: If the pneumothorax recurs (happens again) or if the air leak continues, surgery might be required to close the source of the air leak. In some cases, a procedure known as pleurodesis is performed, in which a substance is inserted into the pleural space causing inflammation and eventually causing the lung to stick to the chest wall, reducing the risk of future pneumothoraces. This is usually done via a minimally invasive procedure known as video-assisted thoracoscopic surgery (VATS).

Supplemental Oxygen: In some cases, extra oxygen may be given. This can help the body absorb the air in the pleural space faster.

Patients are usually monitored closely until the pneumothorax is fully resolved. Treatment can often prevent complications and future occurrences. If you experience symptoms such as sudden chest pain or shortness of breath, it's important to seek medical attention immediately.

11. How is a Port-Infection treated?

Treatment for a port infection generally involves the following steps:

Antibiotics: The first line of treatment for any infection is usually antibiotics. The specific antibiotic used will depend on the type of bacteria causing the infection. Sometimes, the bacteria may be resistant to certain antibiotics, so a sample (culture) may be taken to identify the exact bacteria and determine the best antibiotic to treat it.

Port Removal: If the infection is severe or doesn't respond to antibiotics, it may be necessary to remove the port. This is usually done in a hospital setting under local or general anesthesia. After the port is removed, you will typically continue to receive antibiotics to ensure the infection is completely cleared.

Drainage of Abscess: If an abscess (a pocket of pus) has formed around the port, it may need to be drained. This is usually done using a needle and syringe under sterile conditions.

In all cases, it's important to get medical attention if you notice signs of an infection, which can include redness, swelling, warmth, or drainage at the port site, or symptoms like fever or chills. Infections can be serious if not treated promptly, so don't hesitate to contact your healthcare provider if you have any concerns.

After a port infection, it's important to follow all of your healthcare provider's instructions for care and follow-up. You'll need to ensure the infection has completely cleared before a new port is implanted, if necessary. Your healthcare provider will guide you through this process and help you decide the best course of action based on your individual health needs and circumstances.

Postinterventional aftercare:

1. Will I have pain after the surgery?

After port implantation surgery, it's common to experience some discomfort or pain at the site of the procedure. This can vary from person to person, but typically the pain is mild to moderate and improves over a few days.

Your healthcare team will provide you with instructions and options for managing any pain. This can include:

Prescription pain medications: Your doctor might prescribe a short course of pain medication for you to take after the procedure. It's important to take these only as directed by your healthcare provider.

Over-the-counter (OTC) pain relievers: Non-prescription drugs such as acetaminophen (Tylenol) or non-steroidal anti-inflammatory drugs (NSAIDs) like ibuprofen (Advil, Motrin) can also be used to help manage discomfort. Again, these should be used as directed by your healthcare provider.

Ice packs: Applying an ice pack to the area can help reduce swelling and relieve pain. Be sure to wrap the ice pack in a thin cloth to avoid freezing the skin, and only apply it for short periods of time (around 15-20 minutes) with breaks in between.

While it's normal to have some pain after surgery, if the pain becomes severe, doesn't improve with medication, or is accompanied by other symptoms such as fever, redness, or swelling, you should contact your healthcare provider as these could be signs of complications.

Always follow the advice of your healthcare provider for managing postoperative pain, and never hesitate to contact them if you have any concerns or questions.

2. How long does the local anaesthesia last?

Local anesthesia, the type often used for procedures like port placement, typically lasts for a few hours. The exact duration can depend on several factors, including the type of local anesthetic used, the dose given, and individual patient factors.

Once the local anesthesia wears off, you may start to feel some discomfort or pain at the site of the procedure. This is normal and can usually be managed with over-the-counter or prescribed pain medications as advised by your healthcare provider.

If you experience severe pain, or if the pain continues for a prolonged period, you should contact your healthcare provider as this could be a sign of complications. They can evaluate your symptoms and recommend the appropriate treatment.

3. Can I drive a car after the surgery?

After port implantation surgery, you should not drive home yourself. This is because the procedure often involves the use of medications for sedation or anesthesia that can impair your reflexes and judgement for several hours after the procedure. Even if local anesthesia was used and you feel alert, it's still best to arrange for someone else to drive you home.

As for when you can resume driving after the procedure, this can depend on a few factors, including how you're recovering, whether you're experiencing any side effects from the procedure or medications, and the advice of your healthcare provider.

As a general guideline, many people are able to resume driving within a few days after the procedure, once they're no longer taking prescription pain medications and feel comfortable performing the necessary actions to drive safely, like turning the steering wheel and looking over their shoulder.

But it's very important to follow your healthcare provider's specific advice about when it's safe for you to drive after your procedure. They know your individual circumstances and are the best resource for guidance on this matter.

4. When can I start eating again?

After port implantation surgery, you can typically start eating and drinking again once you're fully awake and the healthcare team has given you the go-ahead. Depending on the specifics of your surgery and your personal health situation, this might be a few hours after the procedure.

In many cases, it's recommended to start with clear liquids or light foods and gradually progress to your normal diet if you're not experiencing nausea or vomiting. It's also important to stay hydrated by drinking plenty of fluids.

However, the exact timing and diet can vary depending on individual circumstances, including the type of anesthesia used during the procedure, your overall health, and the specifics of your recovery. Your healthcare provider will give you specific instructions about when and what you can eat after the procedure. Always follow their instructions and ask them any questions you have about your post-procedure diet.

5. When can the port be used?

In many cases, the port can be used almost immediately after it has been implanted. This is one of the advantages of port catheters, particularly in situations where medication needs to be administered promptly, such as in chemotherapy.

However, the exact timing can depend on a few factors, including the specifics of your surgery, your personal health situation, and your healthcare provider's discretion. They will guide you on when the port is ready to be used.

Remember that even though the port can be used soon after implantation, the surgical site still needs time to heal. It's important to take care of the site and watch for any signs of complications, like

redness, swelling, or unusual discomfort. Always follow your healthcare provider's instructions regarding port care and use.

6. Will the port be visible after the surgery?

After the surgery, the port itself is generally not visible, as it's placed under your skin. However, you might be able to see or feel a small bump under your skin where the port is located, particularly if you are slim or if the port is placed in an area with little fatty tissue.

While the port is typically designed to be discreet and not interfere with daily activities, its presence can sometimes be noticeable depending on the clothing you wear. You might also be able to feel the port when you touch the area.

In terms of the surgical site, there will likely be a small scar once the incision heals. This scar tends to fade over time, but its visibility can depend on individual factors like your skin type and how your body heals.

If you have any concerns about the visibility or feel of the port, be sure to discuss them with your healthcare provider. They can provide you with more information based on your specific circumstances and the specific type of port being used.

7. Can I engage in sports with the port?

Generally, you can participate in many types of physical activities and sports after a port has been implanted and the incision has fully healed. However, there are some important considerations and precautions to take:

Contact Sports: You should avoid sports or activities that involve significant physical contact or risk of injury to the chest area. This includes sports like football, rugby, and martial arts. These types of activities can potentially damage the port or dislodge it from its position.

Swimming and Water Activities: After the port site has healed, swimming is generally allowed. However, it's important to avoid swimming in bodies of water that might be contaminated, such as lakes or rivers, as these can increase the risk of infection.

High-Impact Sports: High-impact sports, like running or jumping, can potentially cause discomfort if the movement causes the port to bounce. Wearing supportive clothing might help in such cases.

Heavy Lifting: Lifting heavy objects can put strain on the muscles and tissues around the port and should be avoided.

It's always important to discuss your specific situation with your healthcare provider before returning to or starting a new sport or physical activity after port placement. They can provide guidance based on your individual health needs, the type of port you have, and the sport you want to participate in.

Remember, taking care of the port and avoiding potential complications is crucial. Always listen to your body and seek medical attention if you experience pain or discomfort during physical activity, or if you notice any changes in the port site.

8. Will I have a scar?

After port implantation surgery, there will be a scar where the incision was made to insert the port. The size and appearance of the scar can vary depending on several factors:

Surgical Technique: The surgical technique used to implant the port can impact the size and location of the incision, and therefore the scar.

Healing Process: Individual healing processes can significantly affect the appearance of the scar. Some people naturally form thicker or more visible scars than others.

Scar Care: How the scar is cared for after the surgery can also affect its final appearance. Keeping the wound clean, protecting it from sun exposure, and possibly using scar care creams or silicone sheets can all help to improve the appearance of the scar over time.

Time: Scars typically fade and become less noticeable over time. However, the rate and extent of this process can vary widely from person to person.

If you're concerned about scarring, you can discuss this with your healthcare provider before the procedure. They can provide information on the surgical technique, expected scar size, and any potential strategies for minimizing scar appearance. In some cases, a plastic surgeon may be consulted to optimize the cosmetic outcome.

9. How should the port be cared for?

Caring for your port is important to help prevent infections and ensure that it functions properly. Here are some general guidelines:

1. **Cleanliness:** Keeping the port and the skin around it clean is key. Usually, your healthcare provider will give you specific instructions on how to clean the area. This might involve cleaning it with a specific type of antiseptic before the port is used, and perhaps at regular intervals even when it's not being used.

2. **Regular Flushing:** Ports need to be regularly flushed to prevent blockages. This usually involves injecting a sterile saline or heparin solution into the port. Your healthcare team will likely do this for you, but they might also teach you how to do it at home.
3. **Protecting the Site:** Avoid pulling or putting strain on the area of the port to prevent dislodging it. Also, avoid swimming in non-chlorinated water or taking baths until the site has fully healed to reduce the risk of infection. Showers are typically okay, but it's important to dry the area thoroughly afterward.
4. **Regular Check-ups:** Regular follow-up appointments with your healthcare provider are important to check the condition and function of the port and to address any problems or complications promptly.
5. **Monitoring for Complications:** It's important to regularly check the port site for signs of infection, such as redness, swelling, increased warmth, or pus. Also, pay attention to symptoms like fever, chills, or unexplained body aches, as these might indicate a systemic infection. If the port appears to be blocked or isn't working properly, contact your healthcare provider.

Remember, the exact instructions can vary depending on the specific type of port, your overall health condition, and your healthcare provider's protocols. Always follow the instructions given by your healthcare provider, and don't hesitate to contact them if you have any concerns or questions.

10. Will I feel the port under the skin?

Yes, in most cases, you will be able to feel the port under your skin. While it is implanted beneath the skin, the port itself is a small device made from plastic or metal, so it will create a small raised area or bump where it's located.

The size and shape of the bump will depend on the specific type of port used. Some are quite flat and may not be very noticeable, especially in individuals with more fatty tissue in the chest area, while others may create a more noticeable bump.

It's important to note that while you will likely be able to feel the port under your skin, it should not cause significant discomfort. If you do experience discomfort or pain related to the port, be sure to let your healthcare provider know as it may be a sign of a problem such as infection or a dislodged port.

11. Does it hurt to access the port?

Accessing a port involves inserting a special needle through the skin and into the port's septum. While this might cause some discomfort, it generally hurts less than a typical needle stick because the needle used to access the port is very thin. Also, because the port is located under the skin, the skin and tissue around the port usually become less sensitive over time.

If you find the process painful, a topical anesthetic cream or gel can be applied to the skin over the port prior to accessing it. This numbs the skin and helps reduce any discomfort from the needle stick.

Once the needle is in place and the port is being used for infusion or to draw blood, you shouldn't feel any pain. If you do feel discomfort or pain during or after the port access, let your healthcare provider know right away, as this might indicate a problem that needs to be addressed.

In general, ports are designed to make repeated access to a vein more comfortable and convenient, and most patients tolerate port access well. However, individual experiences can vary, and your healthcare provider can work with you to make the process as comfortable as possible.

12. How long can I keep the port?

Port catheters are designed for long-term use and can remain in place for months to even years. The exact duration depends on your individual medical needs and treatment plan. They can be extremely beneficial for patients who require long-term intravenous therapy, such as chemotherapy for cancer.

Factors that influence how long you keep the port include:

Type and duration of treatment: If you're undergoing a treatment that requires frequent or long-term access to your bloodstream, such as chemotherapy or long-term antibiotic treatment, you may need to keep the port for the duration of your treatment.

Individual health condition: The status of your health condition may affect how long the port stays in. If your condition improves and you no longer need the port, it can be removed.

Condition of the port: If there are any complications, such as infection, clot formation, or mechanical issues with the port, it may need to be removed or replaced.

Remember, your healthcare provider will be the one to determine the necessity and duration of the port based on your individual health status and treatment needs. Regular check-ups will be scheduled to monitor the condition of the port and ensure it's functioning properly.

Once the port is no longer needed, a minor surgical procedure is performed to remove it.

13. Can I undergo CT or MRI with the port?

    Yes, in most cases, you can undergo computed tomography (CT) and magnetic resonance imaging (MRI) scans with a port in place. Most port catheters are made from materials that are safe for these imaging procedures.

For MRI specifically, most ports are constructed from non-magnetic or weakly magnetic materials that will not be affected by the MRI's magnetic field. However, not all ports are MRI-compatible. Therefore, it's essential to let your healthcare provider and the radiology department know that you have a port catheter before undergoing an MRI scan. They will be able to confirm whether your specific port is safe for MRI.

For CT scans, the port can actually aid in the procedure if intravenous contrast material is needed. The contrast can be injected through the port instead of requiring a separate intravenous line.

In all cases, you should always inform any healthcare provider about your port before undergoing any type of medical test or procedure, not just imaging scans. They will be able to give the most accurate information based on the specifics of your port and the procedure being performed.
