# Supplementary material for: Feasibility of GPT-3 and GPT-4 for in-Depth Patient Education Prior to Interventional Radiological Procedures: A Comparative Analysis
Source: Cardiovasc Intervent Radiol. 2023 Oct 23;47(2):245–50. doi: 10.1007/s00270-023-03563-2 (PMC10844465; doi:10.1007/s00270-023-03563-2)
Supplement: Supplementary file 5 — Supplementary file5 (PDF 274 kb) [file 270_2023_3563_MOESM5_ESM.pdf]

GPT-4 PTA

General Questions:

1. What is a PTA?

Percutaneous transluminal angioplasty (PTA) is a minimally invasive procedure performed to widen narrowed or obstructed arteries or veins, typically to treat arterial atherosclerosis. This procedure was first performed in the peripheral arteries and is still often used in this context.

During the procedure, an empty and collapsed balloon on a guide wire, known as a balloon catheter, is passed into the narrowed locations and then inflated to a fixed size. The balloon forces the expansion of the blood vessel and the surrounding muscular wall, allowing an improved blood flow. A stent may or may not be inserted at the time of ballooning to ensure the vessel remains open. The balloon is then deflated and withdrawn.

PTA is particularly useful in treating peripheral arterial disease (PAD), where blood circulation is restricted due to the buildup of plaque in the arteries, often in the legs.

Please note that while the procedure can greatly aid in the reduction of symptoms (such as pain while walking in PAD), it is often part of a larger treatment plan that involves lifestyle changes to halt or reverse the progression of disease, including dietary modifications, exercise, and medication.

2. How is a stenosis treated in PTA?

A stenosis is a narrowing of a blood vessel, often due to the buildup of plaque (a condition called atherosclerosis). In percutaneous transluminal angioplasty (PTA), a stenosis is treated using the following general steps:

Access: A small incision is made, often in the groin area, to access the femoral artery. A local anesthetic is typically used for this.

Guidewire and Catheter Insertion: A long, thin, flexible tube called a catheter, which has a small balloon attached to it, is threaded through the blood vessels to the site of the stenosis under the guidance of a special type of X-ray called fluoroscopy. A thin wire, known as a guidewire, is typically passed through the vessels first to guide the balloon catheter to the right location.

Balloon Inflation: Once the balloon is positioned within the stenosis, it is inflated. This compresses the plaque against the arterial wall, effectively widening the artery and improving blood flow.

Stent Placement (If Necessary): Depending on the size, location, and composition of the stenosis, as well as the patient's overall health and specific medical condition, a stent (a small wire mesh tube) may be placed at the site of the stenosis during the procedure. The stent is delivered to the site on a balloon catheter. When the balloon inflates, the stent expands and locks into place within the artery. After the balloon is deflated and removed, the stent stays in place permanently to help keep the artery open.

Catheter Removal: After the procedure, the catheter is removed, and the incision site is closed and bandaged.

Remember, all medical procedures, including PTA, carry some risks and potential complications, and they may not be appropriate for all patients. The decision to perform PTA and potentially place a stent is made based on a thorough evaluation of the patient by a healthcare provider.

3. What is a stent?

A stent is a small, flexible tube made of metal or plastic that's used to support the internal structure of a blood vessel, duct, or other anatomical structure. In the context of percutaneous transluminal angioplasty (PTA), a stent is often used to help keep an artery open after it's been widened.

There are two primary types of stents used in PTA:

Bare-Metal Stents: These are stents without any additional coating or medication. They are simply a mesh tube made from a metal alloy.

Drug-Eluting Stents: These stents are coated with medication that is slowly released into the artery following placement. The medication helps to prevent the artery from becoming narrow again (restenosis) by reducing the growth of scar tissue in the artery.

In a PTA procedure, the stent is placed over a balloon catheter in its collapsed state. The balloon catheter is guided to the site of the blockage or narrowing in the artery, and then the balloon is inflated. As the balloon inflates, it expands the stent against the walls of the artery. Once the stent is in place, the balloon is deflated and removed, leaving the stent in place to act as a scaffold that helps keep the artery open. The stent remains in the artery permanently.

As with any medical procedure, the use of stents comes with risks, including potential allergic reaction to the stent material, clot formation within the stent (stent thrombosis), and the potential for restenosis. The use of stents, their type, and the need for subsequent medication (like antiplatelet therapy) are determined by the healthcare provider based on the individual patient's needs and situation.

4. What can be treated with a PTA?

Percutaneous transluminal angioplasty (PTA) is primarily used to treat vascular diseases, where the arteries or veins become narrowed or blocked. The procedure can treat several conditions, including but not limited to:

Peripheral Arterial Disease (PAD): PAD occurs when there's a buildup of fatty deposits or plaque in the peripheral arteries, particularly those supplying the legs and feet. This can cause symptoms such as leg pain when walking.

Coronary Artery Disease (CAD): This is when the coronary arteries, which supply the heart muscle with blood, become narrowed or blocked. The procedure in the context of the coronary arteries is often called percutaneous coronary intervention (PCI) or coronary angioplasty.

Renal Artery Stenosis (RAS): This condition is characterized by narrowing of the arteries that carry blood to the kidneys. If left untreated, RAS can lead to hypertension and kidney failure.

Carotid Artery Stenosis: This condition occurs when the carotid arteries, which are the main blood vessels leading to the brain, become narrowed, often due to atherosclerosis. This can increase the risk of stroke.

Venous Stenosis: PTA can also be used to treat narrowings in the veins, such as in patients with dialysis fistulas or grafts that have become narrow and affect the efficiency of dialysis.

Aneurysm Coiling: In some cases, PTA is used in conjunction with the placement of coils to treat aneurysms, especially those found in the brain.

Remember, the decision to perform PTA and the specific techniques used are based on the individual patient's needs and medical condition. It's important to have a thorough discussion with a healthcare provider to understand the potential benefits and risks of the procedure.

5. Can my peripheral arterial disease (pAD) be improved through a PTA?

Yes, percutaneous transluminal angioplasty (PTA) can significantly improve symptoms associated with peripheral arterial disease (PAD).

PAD is caused by atherosclerosis, a buildup of fatty deposits or plaque in the arterial walls, which can restrict or block blood flow. This is often seen in the arteries of the legs. Symptoms of PAD can include leg pain when walking (claudication), non-healing sores on the feet or legs, and, in severe cases, tissue death (gangrene) which may necessitate amputation.

PTA can help alleviate these symptoms by widening the narrowed or blocked arteries and restoring blood flow. This can lead to improved walking distance, healing of ulcers, and in some cases, preventing limb loss.

Additionally, a stent may be placed during PTA to keep the artery open. The use of drug-eluting stents, which release medication to prevent the re-narrowing of the artery, has shown promise in improving the durability of the procedure.

However, while PTA can treat the symptoms of PAD, it does not cure the underlying disease of atherosclerosis. Therefore, a comprehensive treatment plan often includes lifestyle changes (such as smoking cessation, diet modification, and exercise), medication to control contributing factors (like diabetes, high blood pressure, and high cholesterol), and sometimes medications to prevent blood clotting (antiplatelets or anticoagulants).

Remember that each patient is unique, and the decision to perform PTA depends on many factors, including the severity and location of the blockages, the patient's overall health, and the patient's symptoms and goals of care. You should discuss your options thoroughly with your healthcare provider to understand the potential benefits, risks, and alternatives.

6. Can my peripheral arterial disease (pAD) be cured through a PTA?

Percutaneous transluminal angioplasty (PTA) can significantly improve symptoms and quality of life in patients with peripheral arterial disease (PAD), but it does not cure the underlying disease process.

PAD is caused by atherosclerosis, which is a systemic disease characterized by the buildup of plaque within the arteries. This process often affects multiple blood vessels throughout the body, not just those where symptoms are occurring. Atherosclerosis is often related to modifiable risk factors such as smoking, high cholesterol, high blood pressure, obesity, physical inactivity, and diabetes.

PTA can effectively widen narrowed or blocked arteries and restore blood flow to the affected limbs, often leading to symptom relief, improved mobility, and better quality of life. However, the procedure does not stop the progression of atherosclerosis in other areas of the body.

To manage PAD and atherosclerosis in the long term, it's critical to also address the underlying risk factors. This typically involves lifestyle changes such as:

Regular physical exercise.

A heart-healthy diet.

Smoking cessation.

Weight loss, if overweight or obese.

Good control of blood pressure, blood sugar (if diabetic), and cholesterol levels.

Medications may also be prescribed to help manage these risk factors, as well as to prevent blood clots, which are a potential complication of atherosclerosis.

So while PTA can be a crucial part of managing PAD, it's most effective when used as part of a comprehensive treatment plan that also addresses the root causes of the disease. Always consult with your healthcare provider to discuss the best treatment strategy for your specific situation.

7. When is a PTA performed?

Percutaneous transluminal angioplasty (PTA) is typically performed when patients have symptoms or complications from a narrowed or blocked artery (or vein in some cases), which is not adequately managed by conservative treatments or medications.

The decision to perform a PTA is based on several factors, including the patient's overall health, the severity and location of the blockage, and the impact of the disease on the patient's quality of life. Here are some situations when PTA might be considered:

Peripheral Arterial Disease (PAD): PTA may be considered in patients with PAD who have significant leg pain when walking (claudication) that limits their daily activities and doesn't improve with exercise therapy or medication. It might also be considered for critical limb ischemia, a severe form of PAD characterized by non-healing sores or gangrene due to severely reduced blood flow.

Coronary Artery Disease (CAD): In the setting of CAD, PTA (more commonly called percutaneous coronary intervention or PCI in this context) is often performed in patients with significant blockages that cause symptoms such as chest pain (angina) or shortness of breath, or in those with acute coronary syndrome, a medical emergency that includes conditions such as a heart attack.

Renal Artery Stenosis (RAS): PTA may be done in patients with RAS, particularly those with high blood pressure that's difficult to control with medication, or in those who have declining kidney function due to reduced blood flow to the kidneys.

Carotid Artery Stenosis: PTA of the carotid artery, often with stent placement (carotid stenting), might be considered in certain patients with significant narrowing of the carotid arteries, which can increase the risk of stroke.

Venous Conditions: PTA can also be used to treat certain conditions that cause narrowing or blockages in the veins, such as deep vein thrombosis (DVT), or in patients with dialysis access grafts or fistulas that have become narrowed.

As with any procedure, PTA comes with risks, including complications from the procedure itself and the potential for re-narrowing of the artery (restenosis) over time. Therefore, the decision to perform PTA should involve a careful discussion between the patient and the healthcare provider about the potential benefits, risks, and alternatives.

8. What are the alternatives to a PTA in pAD?

In managing peripheral arterial disease (PAD), various treatment options can be considered, and the choice of treatment often depends on the severity of the disease and the patient's overall health. The goal is to control symptoms, improve quality of life, and halt the progression of the disease. Here are some of the alternatives to percutaneous transluminal angioplasty (PTA):

Lifestyle Changes and Medication: The first line of treatment usually involves lifestyle modifications, such as quitting smoking, increasing physical activity, eating a healthy diet, and managing other risk factors like high blood pressure, high cholesterol, and diabetes. Medications can be used to control these risk factors and to prevent blood clots. For instance, statins are often used to control cholesterol levels, and antiplatelet drugs (like aspirin) may be recommended to reduce the risk of blood clots.

Supervised Exercise Therapy (SET): Exercise, especially a structured program under professional supervision, can significantly improve symptoms of claudication (leg pain with exertion) in PAD. The goal is to increase the distance that the person can walk without pain.

Medications for Claudication: Certain medications, such as cilostazol and pentoxifylline, can help improve walking distance in people with claudication. However, these medications are not appropriate for everyone and can have side effects, so they need to be prescribed by a healthcare provider.

Bypass Surgery: In more severe cases of PAD, particularly when large portions of the artery are blocked, bypass surgery may be considered. This involves using a graft (which may be a blood vessel from another part of the patient's body or a synthetic tube) to reroute blood flow around the blocked area.

Endarterectomy: This is a surgical procedure in which plaque is physically removed from the artery. This procedure is sometimes used for blockages in the femoral artery in the groin, which is a common site of significant blockage in PAD.

Atherectomy: This is a minimally invasive procedure in which a catheter with a sharp blade on the end is used to remove plaque from a blood vessel. The catheter is guided to the location of the blockage, and the blade is used to shave or cut away the plaque.

Each of these treatment options has benefits and risks, and not all options are suitable for every patient. The choice of treatment should be a shared decision-making process between the patient and the healthcare provider, taking into account the patient's symptoms, the severity and location of the disease, the patient's overall health, and their preferences and goals of care.

9. What are the success rates of a PTA?

Percutaneous transluminal angioplasty (PTA) is generally considered a safe and effective procedure for improving blood flow in narrowed or blocked arteries, with immediate success rates often reported over 90% in terms of technical ability to dilate the artery and improve blood flow.

However, one challenge with PTA is the risk of restenosis, or re-narrowing of the artery after the procedure. The rate of restenosis can vary depending on several factors, including the size, location, and severity of the arterial blockage, whether a stent was used, the type of stent used (drug-eluting stents tend to have lower restenosis rates than bare-metal stents), and individual patient factors such as whether they have diabetes or continue to smoke.

In the context of peripheral arterial disease (PAD), studies have reported a wide range of restenosis rates after PTA, with some studies reporting rates as low as 20% and others as high as 60% or more at 1 year after the procedure. The introduction of drug-eluting stents and drug-coated balloons has led to lower restenosis rates in more recent years, although these technologies may not be suitable or available for all patients.

It's also important to remember that while PTA can improve symptoms and quality of life, it does not cure the underlying disease process of atherosclerosis. Therefore, long-term management of PAD also involves lifestyle changes and medications to control risk factors like high blood pressure, high cholesterol, diabetes, and smoking.

Every patient is unique, and outcomes can vary widely based on individual circumstances. Therefore, it's important to have a thorough discussion with the healthcare provider to understand the potential benefits, risks, and alternatives of PTA in the context of the individual patient's situation.

10. What are contraindications for a PTA?

While percutaneous transluminal angioplasty (PTA) is generally considered safe and effective for many patients, there are certain situations or conditions where it may not be the best choice. Some contraindications for PTA include:

Allergy or sensitivity: Patients with a known allergy or sensitivity to the contrast dye used during angioplasty, or to certain materials used in stents, may not be suitable candidates for PTA. However, premedication and use of different types of contrast agents can sometimes allow these procedures to be performed safely even in patients with allergies.

Severe kidney disease: The contrast dye used during PTA can potentially harm the kidneys, especially in patients who already have impaired kidney function. However, the benefits of the procedure might still outweigh the risks in certain situations, so careful consideration and potentially pre- and post-procedural measures to protect the kidneys might be taken.

Certain types of blockages: PTA may not be effective for very long or hard calcified blockages, or for blockages at certain locations such as bifurcations (where a blood vessel splits into two vessels). In these cases, surgical procedures like bypass surgery might be more effective.

Severe comorbid conditions: Patients with severe heart, lung, or other diseases may not be able to tolerate the procedure or the necessary post-procedure medications. In these cases, conservative management strategies might be used instead.

Active infection: If the patient has an active infection, especially in the area where the catheter would be inserted, PTA may need to be postponed until the infection has been treated.

Poorly controlled blood clotting: Patients with a high risk of bleeding, or who can't stop taking anticoagulant medications for a short period of time around the procedure, may not be suitable candidates for PTA.

Remember, each patient is unique, and the decision to perform PTA should involve a careful consideration of the benefits, risks, and alternatives. This requires a thorough discussion between the patient and their healthcare provider.

Preparation and Procedure:

1. How should I prepare for percutaneous transluminal angioplasty (PTA)?

Preparing for percutaneous transluminal angioplasty (PTA) generally involves several steps, including medical evaluations, lab tests, and specific instructions about eating, drinking, and medication use. Here's a general guide, but remember that your healthcare provider will give you personalized instructions based on your health condition and the specific procedure you'll be undergoing.

Medical evaluations: Your healthcare provider will perform a thorough medical evaluation before the procedure. This may include physical examination, reviewing your medical history, and imaging studies such as angiogram to visualize the blockages in your arteries.

Lab tests: You may need to undergo lab tests to check your blood count, kidney function, coagulation profile, and other parameters that might affect the procedure's safety or success.

Medication: If you're taking any medication, especially anticoagulants (blood thinners) or antiplatelet drugs, make sure to inform your healthcare provider. You may need to stop taking these medications for a certain period before the procedure to reduce the risk of bleeding. However, do not stop taking any medication without first discussing it with your healthcare provider.

Eating and drinking: You'll likely be asked to fast (not eat or drink) for a certain period before the procedure, usually at least 6 to 8 hours. This is to reduce the risk of aspiration during sedation.

Arranging transport: You'll probably be sedated during the procedure and may feel groggy afterward, so arrange for someone to drive you home.

Consent form: You'll be asked to sign a consent form before the procedure. Make sure to read it carefully and ask any questions you might have about the procedure's benefits, risks, and alternatives.

On the day of the procedure, wear comfortable clothing, and leave valuables at home. You'll likely need to change into a hospital gown for the procedure.

Again, remember to follow your healthcare provider's specific instructions. Preparation can vary based on individual patient factors and the specifics of the procedure.

2. Will I need to fast before the procedure?

Yes, you will typically need to fast before undergoing percutaneous transluminal angioplasty (PTA). The general recommendation is usually to avoid eating or drinking anything for at least six to eight hours before the procedure. This is to reduce the risk of aspiration (inhalation of stomach contents into the lungs) as sedation or anesthesia will be used during the procedure.

However, the exact duration of fasting may depend on factors such as your overall health, the specifics of the procedure, and the type of sedation or anesthesia used. Always follow the specific instructions given to you by your healthcare provider.

Importantly, if you take medications for conditions like diabetes, heart disease, or high blood pressure, you should discuss with your healthcare provider how to manage these medications around

the time of the procedure, as you may be instructed to take them with a small sip of water even during your fasting period.

Remember not to stop taking any prescribed medications without first consulting your healthcare provider.

3. Should I continue taking my medications before PTA?

Whether or not to continue taking your medications before a percutaneous transluminal angioplasty (PTA) will depend on the specific medications and your overall health condition.

Certain medications may need to be adjusted or temporarily stopped before the procedure. This is especially true for:

Blood-thinning medications: Medications such as warfarin, clopidogrel, and other anticoagulants or antiplatelet agents may need to be adjusted or temporarily stopped before the procedure to minimize the risk of excessive bleeding. Your doctor will provide specific instructions based on the type of medication, your health status, and the specifics of your procedure.

Diabetes medications: If you are taking medication for diabetes, adjustments may be needed since you will be fasting before the procedure.

Other medications: You should discuss all the medications you're currently taking, including over-the-counter drugs, supplements, and herbal products, with your healthcare provider to determine whether any changes are necessary before the procedure.

In many cases, you may be advised to take your usual medications with a small sip of water on the morning of your procedure.

Remember, you should not stop or change the dose of any medication without discussing it with your healthcare provider. Follow the specific instructions given by your healthcare team.

4. Will I be awake during the procedure?

During a percutaneous transluminal angioplasty (PTA), patients are usually given a sedative to help them relax and may also receive a local anesthetic to numb the area where the catheter will be inserted. This means you will be awake during the procedure but should be relaxed and not feel any significant discomfort.

The level of sedation can vary depending on the specifics of the procedure and the patient's individual needs. Some people are mildly sedated and remain fairly alert during the procedure, while others are more heavily sedated and may have little or no memory of the procedure afterwards.

In some cases, especially for more complex procedures or if the patient is particularly anxious, general anesthesia may be used, in which case the patient would be fully asleep for the procedure.

It's important to discuss your concerns and preferences with your healthcare provider before the procedure. They can explain what to expect and discuss the most appropriate type of sedation or anesthesia for your situation.

5. How is the procedure performed in patients with peripheral arterial disease?

Percutaneous transluminal angioplasty (PTA) is a minimally invasive procedure used to treat peripheral arterial disease (PAD) by reopening blocked arteries and restoring blood flow. Here's a general overview of how the procedure is performed:

Preparation: The patient is prepared for the procedure, usually in a specialized catheterization lab or radiology suite. This includes providing sedation and local anesthesia, positioning the patient on the table, and preparing the site of catheter insertion, often the groin or wrist, with antiseptic to reduce the risk of infection.

Accessing the Artery: A small incision is made at the access site, and a thin, flexible tube called a catheter is inserted into the artery.

Guiding the Catheter: Using fluoroscopy (a type of X-ray imaging), the physician guides the catheter through the arteries to the site of the blockage. A contrast dye is injected through the catheter to make the arteries visible on the X-ray images.

Dilating the Artery: Once the catheter reaches the blockage, a smaller, thinner tube with a balloon at its tip is passed through the catheter to the site of the blockage. The balloon is then inflated, pushing against the walls of the artery to widen the narrowed or blocked area and improve blood flow.

Stent Placement: In many cases, a stent (a small, mesh-like tube) is placed at the site of the blockage to help keep the artery open. The stent is mounted on the balloon catheter and expands when the balloon is inflated. Once the balloon is deflated and removed, the stent stays in place, acting like a scaffold to keep the artery open.

Completing the Procedure: After the artery has been dilated and the stent placed (if used), the balloon catheter is removed. The initial incision is closed, often with manual pressure or a small device to seal the puncture site.

Recovery: The patient is monitored for a period of time after the procedure to make sure there are no immediate complications, such as bleeding from the catheter insertion site or an allergic reaction to the contrast dye. Depending on the specifics of the procedure and the patient's overall health, they may be able to go home the same day, or they may need to stay in the hospital overnight for observation.

While PTA can effectively treat PAD and relieve symptoms, it's important to remember that it doesn't cure the underlying disease process of atherosclerosis. Therefore, long-term management of PAD also includes lifestyle changes and medications to manage risk factors like high cholesterol, high blood pressure, diabetes, and smoking.

6. How long will the procedure take?

The length of a percutaneous transluminal angioplasty (PTA) procedure can vary significantly depending on a variety of factors, including the complexity of the arterial blockages, the number of blockages to be treated, the patient's overall health, and whether or not a stent is being placed.

Generally speaking, the procedure can take anywhere from 1 to 3 hours on average. However, more complex cases might take longer.

After the procedure, you will likely need to remain at the hospital for a few hours for monitoring. In some cases, an overnight stay may be required. This allows the healthcare team to monitor for any immediate complications, such as bleeding from the catheter insertion site or an allergic reaction to the contrast dye.

Your healthcare provider will give you more specific information based on your individual situation and the specifics of the planned procedure.

7. Will I feel any pain during PTA?

During a percutaneous transluminal angioplasty (PTA) procedure, you will likely be given sedatives to help you relax, and a local anesthetic will be used to numb the area where the catheter is inserted. This means you should not feel significant pain during the procedure.

You might feel some pressure or discomfort when the catheter is inserted and moved through the artery, but this is usually well-tolerated by patients. If a balloon is used to open up a blockage in an artery, you may feel some discomfort when the balloon is inflated.

After the procedure, you may feel some soreness or discomfort at the catheter insertion site. Over-the-counter pain relievers or prescription medications can be used to manage this post-procedure pain, if needed.

Your healthcare team will monitor your comfort level throughout the procedure and afterwards. If you feel any significant discomfort or pain, it's important to let them know so they can manage your symptoms appropriately.

Again, everyone's experience with a procedure like PTA can vary, and it's important to discuss any concerns or questions you may have with your healthcare provider.

8. What happens if a complication is discovered during the procedure?

While percutaneous transluminal angioplasty (PTA) is generally considered safe, like any medical procedure, it carries a risk of complications. These risks are usually low, but they can be serious. Your healthcare team is prepared to manage any complications that may arise during the procedure.

Complications can include:

Dissection or perforation of the artery: If a tear or hole occurs in the artery during the procedure, it can cause bleeding or other complications. This is usually managed by placing a stent at the site of the injury, or in more severe cases, by performing emergency surgery.

Embolism or thrombosis: Clots can form on the catheter or the stent and can block blood flow. Medications can be used to dissolve clots, or in some cases, other procedures may be used to remove the clot.

Allergic reactions: An allergic reaction can occur in response to the contrast dye used during the procedure. If an allergic reaction occurs, medications can be administered to manage the symptoms.

Kidney injury: The contrast dye can also potentially harm the kidneys, especially in patients who already have impaired kidney function. Measures are taken before, during, and after the procedure to minimize this risk.

Radiation exposure: Although the amount of radiation used in this procedure is usually low, there is always some risk associated with radiation exposure. The procedure is designed to minimize this exposure.

Access site complications: Complications at the site where the catheter was inserted, such as bleeding or infection, can occur. These are usually managed by applying pressure, using medications, or, in rare cases, with surgery.

Your healthcare team is prepared to manage any of these complications and will closely monitor your condition throughout the procedure. After the procedure, you will continue to be monitored to make sure any potential complications are identified and managed promptly.

Remember, the risk of complications must be weighed against the potential benefits of the procedure. In most cases, the benefits of improving blood flow and relieving symptoms outweigh the risks. If you have any concerns, it's important to discuss them with your healthcare provider.

9. Will I be able to feel or sense anything in my leg during the procedure?

During percutaneous transluminal angioplasty (PTA), you are usually given sedative medication to help you relax and a local anesthetic is used to numb the area where the catheter is inserted, typically in the groin or wrist. This means you should not feel pain from the catheter insertion.

As the catheter is guided through your arteries to the site of the blockage, you generally won't feel this happening. The inside of your arteries doesn't have the same sense of touch as your skin, so you won't feel the catheter moving through your body.

However, if a balloon is used to open up a blockage in your leg artery, you may feel some temporary discomfort in your leg when the balloon is inflated. This discomfort usually resolves once the balloon is deflated.

After the procedure, it's normal to have some soreness or minor pain at the catheter insertion site. If a blockage in your leg artery was treated, you might notice improved symptoms like less leg pain during walking.

Remember, everyone's experience can be a bit different, and it's important to communicate with your healthcare team about any concerns or questions you have. They can provide more detailed information based on your specific circumstances.

10. What type of anesthesia or sedation can be used during PTA?

During a percutaneous transluminal angioplasty (PTA), patients are typically given a combination of sedative and local anesthetic. The type and amount of sedation can vary depending on the specific procedure, patient's overall health, and their comfort level. Here are the most common types of sedation or anesthesia used:

Conscious Sedation: Also known as moderate sedation, conscious sedation helps you relax and may make you feel drowsy but you will remain awake and able to respond to commands. This sedation is

generally administered intravenously. Conscious sedation usually also involves the use of pain relievers.

Local Anesthesia: This is used to numb the area where the catheter will be inserted, typically the groin or wrist. This means you should not feel any pain at the insertion site.

Monitored Anesthesia Care (MAC): This involves sedation given by an anesthesiologist or nurse anesthetist, with the depth of sedation ranging from conscious sedation to general anesthesia, depending on the needs of the procedure and patient. The anesthesiology provider also monitors vital signs and manages any complications related to the anesthesia.

General Anesthesia: This is less commonly used for PTA procedures. Under general anesthesia, you are completely unconscious and unaware of the procedure. This may be used in more complex cases or if the patient is particularly anxious or unable to lie still for the procedure.

Your doctor will discuss with you the most appropriate type of sedation or anesthesia for your situation. If you have any concerns about sedation or pain control, be sure to bring them up with your healthcare provider. They can help address your concerns and make you as comfortable as possible during your procedure.

11. Will I need to stay overnight in the hospital after the procedure?

Whether or not you'll need to stay overnight in the hospital after a percutaneous transluminal angioplasty (PTA) can depend on several factors, including:

The complexity of the procedure: More complex procedures may require a longer observation period afterwards.

Your overall health: If you have other health conditions, especially those related to the heart, lungs, or kidneys, you may need to be monitored in the hospital for a longer period.

Potential complications: If there were any complications during the procedure, or if you're at high risk for post-procedure complications, an overnight stay may be necessary.

Your recovery from anesthesia or sedation: Some people may need more time to fully recover from the effects of the sedation or anesthesia used during the procedure.

Many patients undergoing PTA are able to go home the same day, once they have recovered from the sedation and the medical team has ensured there are no immediate complications. However, some patients may need to stay in the hospital overnight for observation.

Your healthcare provider will discuss with you what to expect in your specific case. After you go home, you'll be given instructions on how to care for the catheter insertion site, signs of complications to watch for, and when you can return to your usual activities. You'll also have follow-up appointments to check on your recovery and the results of the procedure.

12. How will I be monitored during the procedure?

During a percutaneous transluminal angioplasty (PTA), your healthcare team will carefully monitor your vital signs and overall wellbeing. Here are some of the common methods used for monitoring during the procedure:

Electrocardiogram (ECG or EKG): This measures the electrical activity of your heart and can help detect any changes in heart rhythm or signs of heart stress during the procedure.

Blood Pressure Monitoring: Your blood pressure will be checked regularly to ensure it remains stable.

Pulse Oximetry: This device, usually placed on your finger, measures the oxygen level in your blood.

Fluoroscopy: This real-time X-ray imaging technique is used to guide the catheter to the right place in your arteries and to monitor the progress of the procedure.

Heart Rate Monitoring: Your heart rate will be continuously monitored throughout the procedure.

Consciousness Level Monitoring: If you're sedated, your level of consciousness will be monitored to ensure the sedation is working properly and that you are comfortable but responsive.

Breathing Rate Monitoring: The rate and depth of your breathing may also be monitored, especially if sedation or anesthesia is used.

Temperature Monitoring: In some cases, your body temperature might be monitored.

The goal of all this monitoring is to ensure your safety and comfort during the procedure, as well as to promptly detect and manage any potential complications. The medical team will be able to respond quickly to any changes in your condition. After the procedure, monitoring will continue for a period of time to make sure you're recovering well.

Risks and Complications:

1. What are the possible risks and complications of percutaneous transluminal angioplasty (PTA)?

Like all medical procedures, percutaneous transluminal angioplasty (PTA) carries some risks and potential complications. While PTA is generally considered a safe procedure and complications are not common, it is important to be aware of the potential risks. These may include:

Bleeding or Hematoma: There is a risk of bleeding or a hematoma (a collection of blood) at the site where the catheter was inserted.

Infection: Any invasive procedure carries a risk of infection, although this risk is relatively low with PTA.

Artery Damage: There is a risk of damage to the artery where the catheter is inserted or the artery being treated, including dissection (a tear in the artery wall) or perforation (a hole in the artery wall).

Blood Clots: Blood clots can form on the catheter or the stent, potentially leading to a blockage in the artery.

Restenosis: The treated artery can narrow again after the procedure, a condition known as restenosis. This is more common when a stent is not used.

Kidney Damage: The contrast dye used in the procedure can, in rare cases, cause kidney damage, particularly in people who already have kidney disease.

Allergic Reactions: Some people may have an allergic reaction to the contrast dye.

Radiation Exposure: There is a risk associated with exposure to radiation from the X-rays used to guide the catheter. However, the level of exposure is generally low and considered safe for most patients.

Stroke or Heart Attack: These are very rare but serious complications. They are more likely to occur in people who have severe heart disease or other serious health conditions.

Your healthcare provider will discuss these risks with you in the context of your specific health status and the potential benefits of the procedure. It's important to ask any questions you have and to understand the potential risks and benefits before deciding to proceed with PTA.

2. How safe is PTA for pAD?

Percutaneous transluminal angioplasty (PTA) is generally considered to be a safe and effective treatment for peripheral arterial disease (PAD). As a minimally invasive procedure, it usually has fewer complications and a shorter recovery time compared to more invasive surgical procedures.

The success of PTA in treating PAD is well established, with many patients experiencing significant improvement in their symptoms and quality of life. However, like all medical procedures, PTA does carry some risk of complications. These risks are generally low, but can include bleeding or bruising at the catheter insertion site, damage to the artery, infection, and restenosis (re-narrowing of the artery).

Certain factors can increase the risk of complications, including the patient's overall health, the specific location and severity of the arterial blockage, and whether a stent is used. It's also worth noting that while PTA can effectively improve blood flow through a blocked artery, it doesn't cure PAD or stop the underlying disease process, so ongoing treatment and lifestyle changes are usually necessary.

As always, the decision to proceed with PTA should be made after a detailed discussion between the patient and their healthcare provider, taking into account the potential benefits and risks in the context of the patient's individual circumstances.

3. Are there any specific factors that increase my risk of complications?

Yes, there are certain factors that may increase the risk of complications from percutaneous transluminal angioplasty (PTA). These can include:

Age: Older patients may have an increased risk of complications, particularly if they have other health conditions.

Pre-existing Medical Conditions: Conditions such as diabetes, heart disease, kidney disease, and high blood pressure can increase the risk of complications.

Smoking: Smoking increases the risk of complications from any surgical procedure, including PTA. It can also increase the risk of restenosis (the artery narrowing again) after the procedure.

Obesity: Obesity can increase the risk of complications, including infection and problems with anesthesia.

Location and Severity of the Blockage: Certain types of blockages, such as long blockages, blockages in smaller vessels, or blockages in certain locations, may have a higher risk of complications or a lower success rate.

Allergies: If you have a known allergy to contrast dye or any of the medications used during the procedure, this can increase the risk of an allergic reaction.

Previous Reactions to Procedures: If you've had complications during previous surgical or medical procedures, you may be at higher risk.

Your healthcare provider will assess your individual risk based on these and other factors. It's important to discuss all of your medical conditions, as well as any concerns you have, with your healthcare provider before the procedure. They will work with you to minimize your risk as much as possible.

4. What steps are taken to minimize the risks during the procedure?

Various steps are taken to minimize the risks and potential complications during a percutaneous transluminal angioplasty (PTA) procedure. These can include:

Pre-procedure Assessment: Before the procedure, your healthcare provider will perform a thorough assessment of your health, which can include blood tests, imaging studies, and other diagnostic tests. This is done to identify any potential risks and ensure you are in the best possible health before the procedure.

Proper Sterilization Techniques: To minimize the risk of infection, strict sterilization techniques are used. This includes cleaning and disinfecting the skin at the catheter insertion site and using sterile equipment.

Use of Local Anesthesia or Sedation: Local anesthesia or sedation is used to help you stay comfortable and relaxed during the procedure, minimizing the stress on your body.

Monitoring During the Procedure: Throughout the procedure, your vital signs will be closely monitored. This includes your heart rate, blood pressure, oxygen levels, and, in some cases, your brain waves (if you're receiving sedation). This continuous monitoring allows the healthcare team to quickly detect and respond to any potential problems.

Use of Anticoagulant Medication: During and after the procedure, you may be given anticoagulant (blood-thinning) medication to help prevent blood clots.

Experienced Medical Team: The procedure should be performed by a skilled and experienced medical team who are trained to handle any complications that may arise.

Post-procedure Care and Monitoring: After the procedure, you'll be closely monitored to ensure you're recovering well and to promptly detect and manage any complications. You'll also be given clear instructions on what to do when you go home, including how to care for the catheter site, what symptoms to watch for, and when to seek medical attention.

Remember, while these steps can significantly reduce the risks associated with PTA, no procedure is completely risk-free. It's important to have a thorough discussion with your healthcare provider about the potential risks and benefits before making a decision about treatment.

5. Can I die during the procedure?

While the risk of death during percutaneous transluminal angioplasty (PTA) is extremely low, as with any medical procedure, there is always a very small risk. Most deaths that do occur are related to complications such as heart attack or stroke, and are more common in patients with severe heart disease or other serious health conditions.

It's important to note that PTA is considered a minimally invasive procedure, and the risks are generally much lower than those associated with more invasive surgical procedures.

Prior to the procedure, your healthcare provider will conduct a thorough assessment of your overall health and the specific characteristics of your peripheral arterial disease (PAD) to evaluate the potential benefits and risks in your individual case. This evaluation can help identify patients who may be at higher risk of complications and guide the decision about whether to proceed with PTA or consider alternative treatment options.

It's also important to remember that while the procedure itself carries some risks, PAD itself also carries risks if left untreated, including severe pain, non-healing sores, gangrene, and the potential need for amputation.

You should discuss any concerns you have about the risks of the procedure with your healthcare provider. They can provide more detailed information based on your specific health status and the specifics of the planned procedure.

6. Are there any complications that can occur after the procedure?

Yes, there are potential complications that can occur after percutaneous transluminal angioplasty (PTA), though they are relatively rare. These might include:

Bleeding or Hematoma: Minor bleeding or bruising at the catheter insertion site is relatively common, but serious bleeding that requires a blood transfusion or surgery is rare.

Infection: There's a small risk of infection at the catheter insertion site.

Restenosis: The treated artery can narrow again after the procedure, a condition known as restenosis. This is more common when a stent is not used.

Thrombosis or Embolism: Rarely, a blood clot (thrombosis) can form in the treated artery, or a piece of a clot (embolism) can break off and block another artery. These are serious complications that require immediate treatment.

Artery Damage: There's a small risk of damage to the artery where the catheter was inserted or to the artery being treated. This could include dissection (a tear in the artery wall), perforation (a hole in the artery wall), or formation of a pseudoaneurysm (a small sac of blood that forms on the side of an artery).

Kidney Damage: In rare cases, the contrast dye used during the procedure can cause kidney damage, particularly in people who already have kidney disease.

Allergic Reaction: Some people may have an allergic reaction to the contrast dye used during the procedure.

It's important to seek immediate medical attention if you experience severe pain, swelling, redness, or heat at the catheter insertion site, or symptoms such as chest pain, shortness of breath, severe leg pain, or sudden weakness or numbness after the procedure.

Remember, most people do not experience serious complications after PTA. Your doctor will explain the signs of potential complications and provide clear instructions on when to seek medical attention.

7. How are intraprocedural complications treated?

Intraprocedural complications, or those that occur during the procedure itself, are typically managed immediately by the interventional team performing the percutaneous transluminal angioplasty (PTA). The type of management depends on the nature of the complication:

Bleeding or Hematoma: Minor bleeding can often be controlled by applying pressure to the site. If a larger hematoma forms, additional interventions may be necessary, such as surgical evacuation or ultrasound-guided compression.

Artery Damage: If there's a dissection (tear) or perforation (hole) in the artery, a stent may be placed to seal it. In rare cases, an emergency surgical repair may be needed.

Thrombosis or Embolism: If a blood clot forms during the procedure, medications can be administered to dissolve the clot. In certain cases, mechanical thrombectomy (physically removing the clot) may be required.

Allergic Reaction to Contrast Dye: If an allergic reaction to the contrast dye occurs, medications such as antihistamines, steroids, or epinephrine may be administered.

Heart Rhythm Disturbances: Any heart rhythm problems that occur during the procedure can be managed with medications or with a temporary pacemaker in rare cases.

Kidney Damage: The risk of kidney damage from contrast dye can be minimized by ensuring adequate hydration before and after the procedure, and using the smallest effective amount of dye. If kidney function deteriorates, additional treatments such as dialysis may be required.

The medical team performing the PTA is trained to recognize and manage these complications promptly. This is one of the reasons why continuous monitoring of your vital signs is done throughout the procedure. Furthermore, before the procedure, you should discuss with your doctor about the risks and benefits and what to expect during and after the procedure.

8. Will there be blood loss during the procedure?

Percutaneous transluminal angioplasty (PTA) is a minimally invasive procedure, and as such, blood loss is typically minimal. However, some bleeding is to be expected, especially at the catheter insertion site.

Most often, this bleeding is minor and can be easily controlled by applying pressure to the area after the catheter is removed. However, in rare cases, there may be more significant bleeding or the

formation of a hematoma (a localized collection of blood outside the blood vessels). These complications are typically managed immediately by the interventional team.

Your healthcare provider will use techniques to minimize bleeding, such as careful placement of the catheter and use of hemostatic devices or pressure bandages after the procedure. If you are on blood-thinning medications (anticoagulants or antiplatelet agents), your healthcare provider will provide specific instructions about how to manage these medications around the time of the procedure to balance the risk of bleeding with the risk of blood clot formation.

While blood loss during PTA is typically minimal, it's important to tell your healthcare provider if you have a history of bleeding disorders or if you're taking any medications that could increase your risk of bleeding.

9. What structures can be injured during the procedure?

During percutaneous transluminal angioplasty (PTA), the aim is to avoid injury to any structures. However, as with any invasive procedure, there is always a risk of injury. Some structures that could potentially be injured during PTA include:

Blood Vessels: The procedure involves inserting a catheter into an artery, typically in the groin or arm, and then guiding it through the blood vessels to the site of the blockage. There's a risk of damaging these blood vessels, leading to complications such as dissection (tearing of the artery wall), perforation (puncturing of the artery), hematoma (blood clot), or bleeding.

Nerves: Although less common, there is a small risk of nerve damage during PTA, especially if the procedure is performed near nerve structures. This could potentially lead to numbness, weakness, or pain.

Kidneys: The contrast dye used during the procedure can, in rare cases, cause kidney damage, particularly in individuals who already have kidney disease or other risk factors.

The likelihood of these injuries is low, especially when the procedure is performed by an experienced team. The interventional team is trained to handle these complications and take necessary precautions to avoid them. For instance, real-time imaging (fluoroscopy) is used during the procedure to guide the catheter and help avoid injury to the blood vessels and surrounding structures.

Postinterventional aftercare:

1. What do I need to consider after the surgery?

After undergoing percutaneous transluminal angioplasty (PTA), there are several factors you'll need to consider as part of your recovery:

Wound Care: You will need to take care of the catheter insertion site to prevent infection and promote healing. Your healthcare provider will give you instructions on how to clean the area and when you can remove any dressings or bandages.

Activity Levels: You may need to avoid heavy lifting, strenuous activities, and driving for a period of time after the procedure. Your healthcare provider will advise you on when you can return to normal activities.

Medication Management: You will likely be prescribed medications after the procedure, such as blood thinners to prevent blood clots, or other medications to manage underlying conditions such as high blood pressure or high cholesterol. It's important to take these medications as instructed and discuss any side effects or issues with your healthcare provider.

Follow-up Appointments: It's crucial to attend all follow-up appointments so your healthcare provider can monitor your recovery and the effectiveness of the procedure. You may need additional imaging studies to evaluate the treated artery.

Lifestyle Changes: Healthy lifestyle habits can help prevent further progression of peripheral arterial disease (PAD) and improve your overall health. This may include eating a healthy diet, engaging in regular physical activity, quitting smoking if you smoke, and managing underlying health conditions such as diabetes.

Monitoring for Complications: Be aware of signs of potential complications, such as severe pain, swelling, or redness at the catheter insertion site, chest pain, severe leg pain, or sudden weakness or numbness. Seek immediate medical attention if you experience any of these symptoms.

Remember, your healthcare provider is your best source of information for your specific situation and will provide personalized advice and instructions based on your overall health, the specifics of the procedure, and your recovery progress.

2. Will I experience pain after the surgery?

After a percutaneous transluminal angioplasty (PTA), it's normal to have some discomfort or mild pain at the catheter insertion site. This is typically mild and can be managed with over-the-counter pain relievers. Your healthcare provider will guide you on what pain medications you can take, especially considering interactions with any blood thinners you may be prescribed.

Other post-procedure symptoms could include mild bruising or swelling at the catheter insertion site, which should also resolve over a few days.

If you experience severe pain, or if the mild pain does not improve over a few days, it's important to contact your healthcare provider. Similarly, severe pain in your leg or elsewhere could be a sign of a complication such as a blood clot and should be evaluated immediately.

Remember, while some discomfort is normal, severe or persistent pain is not and should be evaluated by a healthcare professional. Pain management is an important part of recovery, and effective control of pain can help speed up your recovery and return to normal activities.

3. Can I take pain medication after the surgery?

Yes, it's generally safe to take pain medication after percutaneous transluminal angioplasty (PTA) to manage any discomfort or pain at the catheter insertion site or elsewhere. However, the specific medications you can take may depend on other medications you've been prescribed, your overall health status, and any other underlying health conditions.

For mild pain, over-the-counter pain relievers such as acetaminophen (Tylenol) are often recommended. Nonsteroidal anti-inflammatory drugs (NSAIDs), such as ibuprofen (Advil, Motrin), can also be effective, but should be used cautiously if you have been prescribed blood-thinning medications, as NSAIDs can increase the risk of bleeding.

For more severe pain, your healthcare provider might prescribe stronger pain medications. Always take these exactly as instructed, and avoid taking more than the prescribed dose.

Before taking any new medication, including over-the-counter medications, it's important to discuss it with your healthcare provider to ensure it's safe and won't interact with your other medications. If the pain persists or gets worse, contact your healthcare provider promptly, as it could be a sign of a complication.

4. What kind of care should I provide for the puncture site or incision?

After percutaneous transluminal angioplasty (PTA), taking care of the puncture site or incision is important to promote healing and prevent infection. Here are some general guidelines:

Keep the Area Clean: You may be advised to keep the area clean and dry for a certain period, often 24 to 48 hours. After that, you may be allowed to shower, but avoid soaking the area in water (no baths, swimming pools, or hot tubs) until the site is fully healed.

Check for Signs of Infection: Regularly check the area for signs of infection, which can include increased redness, swelling, warmth, or drainage, as well as fever or chills. If you notice any of these signs, contact your healthcare provider.

Avoid Rubbing or Irritation: Avoid rubbing the site or wearing tight or restrictive clothing that could irritate the area.

Avoid Heavy Lifting or Straining: Following your procedure, try to avoid any activities that could put strain on the site, including heavy lifting or straining, for a period recommended by your healthcare provider.

Follow Specific Instructions: Your healthcare provider will give you specific instructions based on your individual circumstances, so be sure to follow those closely.

Remember, your healthcare provider is your best source of information. They will provide instructions tailored to your specific situation. If you're unsure about anything or have any concerns, don't hesitate to contact them.

5. Are there any specific restrictions or limitations on activities following PTA?

After percutaneous transluminal angioplasty (PTA), there are typically some temporary restrictions on physical activities to allow your body to heal. These can vary depending on your individual situation and overall health, but general guidelines include:

Rest and Gradual Return to Activity: It's normal to feel tired after the procedure, so rest and take it easy for the first few days. Gradually return to your normal activities as you feel able.

Avoid Heavy Lifting and Strenuous Exercise: Your healthcare provider will likely advise you to avoid heavy lifting, strenuous exercise, and any activities that might strain the puncture site for a certain period, often about a week.

Driving: You may be advised not to drive for 24 to 48 hours after the procedure, or longer if you're taking narcotic pain medications.

Bathing: You'll likely be advised to keep the puncture site clean and dry for 24 to 48 hours. After that, you can usually shower, but avoid soaking the area in water (no baths, swimming pools, or hot tubs) until the site is fully healed.

Remember, these are general guidelines and your healthcare provider will provide instructions tailored to your specific situation. Always follow your healthcare provider's advice, and don't hesitate to contact them if you have any questions or concerns. It's also important to attend all follow-up appointments so your recovery can be monitored.

6. How long do I need to remain lying down after PTA?

The amount of time you'll need to stay lying down after percutaneous transluminal angioplasty (PTA) can vary depending on factors like the specific location and size of the treated vessel, the type of

closure device used, and your overall health. However, it's common for patients to be asked to remain lying down for several hours immediately following the procedure.

This period of bed rest is important to ensure that the puncture site in the artery has time to seal properly and reduce the risk of bleeding. During this time, it's important to keep the leg that was treated straight.

Your healthcare team will monitor the puncture site during this period and will let you know when it is safe for you to start moving around again. If you need to cough, sneeze, or move while you are lying down, it's important to let your healthcare team know so they can provide guidance and support.

Again, the specific amount of time can vary, so it's important to follow the guidance of your healthcare team.

7. What signs or symptoms should I watch for that may indicate a problem or complication?

After percutaneous transluminal angioplasty (PTA), you should monitor for any unusual signs or symptoms that could indicate a problem or complication. Contact your healthcare provider if you experience:

Changes at the Catheter Insertion Site: Signs of infection, such as increased redness, warmth, swelling, or pus drainage. Also, look for signs of bleeding or a growing hematoma (bruise or blood clot), like a lump that's increasing in size.

Pain: New or worsening pain at the puncture site or in the leg that was treated.

Fever or Chills: A fever or chills could indicate an infection.

Changes in Leg Appearance or Sensation: Changes such as paleness, blueness, coolness, numbness, tingling, or weakness in the leg that was treated.

Changes in Pulse: An absent or decreased pulse in the leg that was treated.

Leg Swelling: Sudden swelling in the leg that was treated.

Difficulty Breathing or Chest Pain: While less common, these symptoms could indicate a more serious complication such as a heart problem or a blood clot that has traveled to the lungs (pulmonary embolism).

Remember, it's better to be safe and report any concerning symptoms to your healthcare provider promptly. In case of severe symptoms like severe chest pain, severe shortness of breath, or loss of consciousness, seek immediate medical attention.

8. Will I have a scar?

Percutaneous transluminal angioplasty (PTA) is a minimally invasive procedure, meaning it is done through a small puncture in the skin rather than a large incision. As a result, any scar that forms is typically small and may become less noticeable over time. The size and appearance of the scar can depend on factors such as your age, skin type, and how well you heal.

Immediately after the procedure, there may be a small wound or bruise at the catheter insertion site. Over time, this should heal and may eventually appear as a small scar.

It's important to care for the puncture site as instructed by your healthcare provider to promote healing and minimize scarring. This generally involves keeping the area clean and dry, and avoiding scratching or irritating the area.

If you have concerns about scarring, speak with your healthcare provider. They can provide advice tailored to your specific situation and may be able to suggest strategies or treatments to minimize scarring.

9. How long does it take to feel an improvement of my pAD after PTA?

The timeline for experiencing an improvement in symptoms of peripheral arterial disease (PAD) following percutaneous transluminal angioplasty (PTA) can vary greatly from person to person. It largely depends on the severity of your disease, the specific arteries that were treated, your overall health, and your body's healing process.

Many patients notice some improvement in symptoms such as leg pain or cramping (claudication) relatively soon after the procedure, often within a few days to weeks. However, for others, it may take longer to notice a difference, especially if they have severe PAD or other health conditions that impact blood flow.

Remember that while PTA can be very effective at improving blood flow and reducing symptoms, it is not a cure for PAD. Lifestyle changes, such as quitting smoking, eating a heart-healthy diet, exercising regularly, and managing other health conditions like diabetes or high blood pressure, are essential parts of managing PAD and maximizing the benefits of the procedure.

Always consult with your healthcare provider about what to expect following PTA. They can provide the most accurate timeline based on your specific circumstances.

10. How long does the improvement last?

The duration of improvement after a percutaneous transluminal angioplasty (PTA) procedure for peripheral arterial disease (PAD) can vary significantly between individuals. This largely depends on factors like the severity of the disease, the specific arteries treated, the patient's overall health, and the degree to which lifestyle changes are made and maintained.

While PTA can significantly improve blood flow and alleviate symptoms like leg pain and cramping, restenosis, or re-narrowing of the artery, can occur. If a stent was placed during the PTA, it can also become blocked over time, a condition known as in-stent restenosis. Studies have shown that restenosis can occur in a substantial proportion of patients within a year or two following the procedure.

However, maintaining a heart-healthy lifestyle plays a significant role in prolonging the benefits of the procedure. This includes regular exercise, a healthy diet, quitting smoking, and effectively managing other health conditions like diabetes and high blood pressure.

In some cases, repeat procedures may be necessary to maintain optimal blood flow if the artery narrows again. Regular follow-up appointments with your healthcare provider are important for monitoring your progress and catching any potential issues early.

Remember, your healthcare provider is the best source of information for your individual situation. They can give you a more specific idea of what to expect based on your health, the specifics of your procedure, and your ongoing management plan for PAD.

11. Do I need to take any new medications after PTA?

After percutaneous transluminal angioplasty (PTA), your healthcare provider may prescribe new medications or adjust your current ones to help improve your blood flow, prevent blood clots, and manage your peripheral arterial disease (PAD).

Common medications that might be prescribed include:

Antiplatelet or Anticoagulant Medications: These medications, such as aspirin or clopidogrel (Plavix), help to prevent blood clots from forming in your arteries or in a stent, if one was placed during your procedure.

Statins: These cholesterol-lowering medications can help slow the progression of atherosclerosis (hardening and narrowing of the arteries), reduce your risk of future cardiovascular events, and possibly stabilize existing plaque, making it less likely to cause a clot.

Medications for Controlling Symptoms of PAD: Medications such as cilostazol (Pletal) and pentoxifylline (Trental) might be used to improve walking distance in people with PAD, although their effectiveness can vary.

Blood Pressure Medications: If you have high blood pressure, medications may be prescribed or adjusted to help control it.

Antidiabetic Medications: If you have diabetes, it's important to keep your blood sugar levels under control to slow the progression of PAD and other cardiovascular diseases.

Pain Medications: Over-the-counter or prescription pain medications may be given to manage any discomfort following the procedure.

Remember, always take medications as directed by your healthcare provider, and make sure they are aware of any other medications or supplements you are taking, as some can interfere with the effectiveness of others. It's also important to discuss any side effects or concerns you have with your healthcare provider. Following a healthy lifestyle, including regular exercise, a balanced diet, and quitting smoking, also remains an essential part of managing PAD.

12. Can I undergo an MRI with a stent?

Most modern stents used in percutaneous transluminal angioplasty (PTA) are considered MRI-safe, meaning they should not be affected by or cause problems during an MRI scan. These stents are typically made of non- or weakly-ferromagnetic materials (such as certain types of stainless steel, cobalt-chromium, platinum-chromium, or nitinol) that do not significantly interact with the strong magnetic field of an MRI machine.

However, whether it's safe for you to have an MRI will depend on the specific type of stent that was used, how long ago it was placed, and the specific type of MRI scan you need. Some stents may cause image artifacts that could obscure the area being imaged.

Before you have an MRI, it's crucial to inform your healthcare provider and the MRI technician about your stent. If possible, provide them with specific information about the type of stent you have. They can then determine whether it's safe for you to undergo the MRI and make necessary adjustments to the scan to ensure the best possible image quality.

Lastly, other implanted devices or metallic objects within your body (such as pacemakers, certain types of heart valves, aneurysm clips, or metal fragments) can pose a risk during MRI. So, it's crucial to discuss all your medical history with your healthcare provider.

13. Will I need any follow-up appointments?

Yes, after a percutaneous transluminal angioplasty (PTA), you will need follow-up appointments with your healthcare provider. These appointments are an important part of your care and recovery, and allow your healthcare provider to:

Check the Puncture Site: They will inspect the area where the catheter was inserted to ensure it's healing properly and that there are no signs of infection or other complications.

Evaluate Your Symptoms: Your provider will ask about any symptoms you're experiencing, to determine how well the procedure has worked and to catch any potential problems early.

Assess Your General Health: This might include checking your blood pressure, heart rate, and other vital signs, as well as possibly ordering lab tests or imaging studies.

Monitor the Treated Artery: This is often done using non-invasive imaging methods such as ultrasound to ensure the artery remains open and that there's no sign of restenosis (re-narrowing of the artery).

Review or Adjust Medications: They will review the medications you're taking, including any new ones prescribed after the procedure, and make adjustments as necessary.

Discuss Lifestyle Changes: Your healthcare provider will also likely talk to you about lifestyle changes that can help manage your peripheral arterial disease and improve your overall health, such as exercising regularly, eating a heart-healthy diet, quitting smoking, and managing other health conditions like diabetes or high blood pressure.

The frequency and number of these follow-up appointments can depend on factors such as your overall health, the severity of your disease, how well you're recovering from the procedure, and whether or not a stent was placed. It's important to keep all scheduled follow-up appointments and to contact your healthcare provider if you have any concerns between appointments.

14. Are there any lifestyle changes or ongoing treatments I should consider to maintain the benefits of PTA?

Yes, lifestyle changes and ongoing treatments are crucial to managing peripheral arterial disease (PAD) and maintaining the benefits of percutaneous transluminal angioplasty (PTA). Here are some steps you can take:

Smoking Cessation: If you smoke, quitting is one of the most effective steps you can take to slow the progression of PAD and reduce your risk of complications. Smoking damages blood vessels, decreases your ability to exercise, and increases the risk of restenosis (re-narrowing of the artery) after PTA.

Regular Exercise: Regular physical activity can help improve circulation, increase the distance you can walk without pain, and promote overall cardiovascular health. Your healthcare provider or a physical therapist may be able to recommend an exercise program tailored to your needs and abilities.

Healthy Diet: Eating a heart-healthy diet can help control blood pressure, lower cholesterol, manage body weight, and control blood sugar levels. This typically includes lots of fruits, vegetables, whole grains, lean proteins, and healthy fats, while limiting saturated fats, trans fats, sodium (salt), and added sugars.

Manage Other Health Conditions: If you have other health conditions like diabetes or high blood pressure, managing these effectively is crucial to slowing the progression of PAD and preventing complications.

Medication: Continue to take any prescribed medication as directed by your healthcare provider. This may include drugs to prevent blood clots, lower cholesterol, manage symptoms of PAD, control blood pressure, or manage blood sugar levels.

Regular Follow-up Appointments: Regular check-ups with your healthcare provider are important for monitoring your progress, detecting any potential problems early, and making any necessary adjustments to your treatment plan.

Remember, your healthcare provider is your best source of information and guidance. Always discuss any lifestyle changes or treatments you're considering with them before starting. They can provide advice and recommendations based on your specific circumstances, health, and needs.
