# Supplementary material for: Feasibility of GPT-3 and GPT-4 for in-Depth Patient Education Prior to Interventional Radiological Procedures: A Comparative Analysis
Source: Cardiovasc Intervent Radiol. 2023 Oct 23;47(2):245–50. doi: 10.1007/s00270-023-03563-2 (PMC10844465; doi:10.1007/s00270-023-03563-2)
Supplement: Supplementary file 6 — Supplementary file6 (PDF 246 kb) [file 270_2023_3563_MOESM6_ESM.pdf]

# GPT-4 TACE

General questions:

1. What is a TACE?

Transarterial Chemoembolization (TACE) is a minimally invasive procedure primarily used to treat liver cancer. TACE is particularly useful for cancers that have spread to the liver from another part of the body or that have originated in the liver but cannot be surgically removed.

In this procedure, an interventional radiologist uses imaging guidance to insert a catheter into an artery in the groin or wrist and guides it into the artery that feeds the tumor in the liver. Once the catheter is in place, a combination of chemotherapy drugs and embolic agents (small particles or beads) is injected directly into the tumor.

The chemotherapy drugs help to kill the cancer cells, while the embolic agents block the blood flow (embolize) to the tumor, which deprives it of the oxygen and nutrients it needs to grow. This dual action both directly kills the cancer cells and impedes their ability to continue growing and dividing.

The key advantage of TACE is that it allows a high dose of chemotherapy to be delivered directly to the tumor while limiting the drug's exposure to the rest of the body, thereby reducing the overall side effects usually associated with chemotherapy. Additionally, by embolizing the artery, the chemotherapy drug is trapped in the area and its effect on the tumor is prolonged.

2. What can be treated with TACE?

Transarterial Chemoembolization (TACE) is most commonly used to treat cancers that have originated in or spread to the liver. This includes:

Hepatocellular Carcinoma (HCC): HCC is the most common form of primary liver cancer (cancer that starts in the liver). TACE is often used when the cancer is too large for surgery or a liver transplant, or when these options aren't suitable for the patient for other reasons.

Metastatic liver disease: TACE can be used to treat tumors that have spread to the liver from another part of the body, particularly from cancers such as colorectal cancer, neuroendocrine tumors, and certain types of breast cancer.

The effectiveness of TACE may depend on the type and stage of the cancer, the overall health of the patient's liver, and the patient's general health. TACE is often used as part of a multimodal treatment

plan that can include other forms of therapy, such as surgery, radiation, systemic chemotherapy, or targeted therapies.

However, not all liver tumors are suitable for TACE, especially tumors that have extensive involvement of the liver or those in patients with severe underlying liver disease. The decision to use TACE is made after careful evaluation by a multidisciplinary team including hepatologists, oncologists, interventional radiologists, and often surgeons.

3. Which chemotherapeutic agents are used?

The choice of chemotherapeutic agents used in Transarterial Chemoembolization (TACE) can depend on a number of factors, including the type and stage of the cancer, the patient's overall health, and the preferences and experiences of the treating physicians.

That being said, common chemotherapeutic agents used in TACE procedures include:

Doxorubicin: This is a commonly used drug in TACE procedures, especially for hepatocellular carcinoma. It's an anthracycline antibiotic that works by intercalating into DNA, disrupting its function and preventing cancer cells from dividing and growing.

Cisplatin: Cisplatin is a platinum-containing drug that can cause DNA damage in cancer cells, leading to cell death.

Mitomycin C: This drug inhibits DNA synthesis, which blocks cell division.

Gemcitabine: Used primarily for the treatment of pancreatic cancer, it can also be used in TACE for liver metastases from pancreatic cancer.

Often, a combination of drugs is used in order to enhance the anticancer effect and to cover a broad spectrum of potential cancer cell types. The chemotherapy is typically mixed with lipiodol, an oily contrast agent that helps to keep the chemotherapy in the tumor area for a longer time, increasing the effectiveness of the treatment.

It's important to note that the choice of chemotherapy drugs is highly individualized and depends on the specific circumstances of each patient.

4. What is used for embolization?

Embolization in Transarterial Chemoembolization (TACE) involves blocking the blood supply to the cancer cells in the liver. This is accomplished by injecting embolic agents into the blood vessels that

supply the tumor. These agents essentially plug up the vessels and help to trap the chemotherapy agents within the tumor. Various materials can be used for this purpose:

Ethiodized Oil (Lipiodol): Lipiodol is a type of iodine-rich poppyseed oil that is often mixed with the chemotherapy drugs before being injected into the liver. It not only helps to block the vessels, but also enhances the delivery and retention of the chemotherapy drugs in the tumor.

Embolization Particles: These can be gelatin sponge particles, polyvinyl alcohol (PVA) particles, or specially designed microspheres. They are designed to block the small blood vessels within the tumor.

Drug-Eluting Beads: These are small beads that are loaded with chemotherapy drugs. They provide both embolization and sustained, controlled release of the chemotherapy drugs into the tumor.

The choice of embolic agent may depend on the specifics of the patient's case, the nature of the tumor, and the preferences of the treating physician. The goal is to achieve effective blockage of the tumor's blood supply while maintaining the health of the surrounding liver tissue.

5. What are the success rates of TACE?

The success rate of Transarterial Chemoembolization (TACE) can depend on a number of factors, including the type and stage of cancer, the size and location of the tumors, the overall health of the patient's liver, and the patient's general health. It's also important to remember that "success" can mean different things in different contexts: for some patients, the goal might be to shrink the tumor before surgery, while for others it might be to extend life or improve quality of life.

With that said, studies have shown that TACE can be effective in certain situations. For instance, it is widely accepted that TACE can improve survival for patients with intermediate-stage hepatocellular carcinoma (HCC, the most common type of liver cancer) who are not candidates for curative treatments like resection, ablation or transplantation.

According to a widely-cited randomized controlled trial published in the Lancet in 2002, TACE improved 2-year survival rates in patients with unresectable HCC from 63% (for patients who did not receive TACE) to 75% (for those who did).

In terms of tumor response, some studies have reported that up to 50-60% of HCC tumors show a partial response to TACE (meaning the tumor shrinks but does not completely disappear), and a smaller percentage may show a complete response.

For metastatic liver cancer, the success of TACE is highly variable and depends largely on the type and aggressiveness of the primary tumor.

It's important to note that these are average numbers and the actual outcome can vary significantly from patient to patient. The decision to use TACE should be made in consultation with a multidisciplinary team of medical professionals, who can help assess the potential risks and benefits in the context of the individual patient's case.

6. What are the contraindications for TACE?

While TACE (Transarterial Chemoembolization) is a generally safe and effective treatment option for liver cancer, there are certain contraindications or situations in which the procedure may not be suitable. Contraindications can vary depending on the specific patient and clinical factors, but here are some common contraindications for TACE:

Severe liver dysfunction: Patients with significantly impaired liver function, such as advanced cirrhosis or severe liver failure, may not be suitable candidates for TACE. The procedure requires adequate liver function to tolerate the effects of chemotherapy and embolization.

Inadequate liver reserve: TACE may not be recommended if the patient has limited liver reserve, indicated by poor synthetic function, severe portal hypertension, or significant ascites. Insufficient liver function can increase the risk of complications and limit the potential benefits of the procedure.

Vascular anatomy unsuitable for embolization: The presence of abnormal blood vessel anatomy or extensive collateral circulation may make it difficult to perform effective embolization. In such cases, TACE may not be feasible or may have limited efficacy.

Extrahepatic disease: TACE is primarily intended for treating liver tumors. If the cancer has spread extensively outside the liver (such as to distant organs), TACE may not provide substantial benefit and alternative treatment options may be considered.

Poor performance status: Patients with a significantly compromised overall health status, such as those with a very poor performance status or multiple comorbidities, may not be able to tolerate the procedure or may have limited life expectancy.

Allergy or intolerance to the chemotherapy agents or contrast media: If a patient has a known allergy or severe intolerance to the chemotherapy drugs used in TACE or the contrast media required for imaging, alternative treatment options should be explored.

7. What are alternatives to TACE?

There are several alternatives to Transarterial Chemoembolization (TACE) for treating liver cancer, and the best approach can depend on a number of factors, including the type and stage of the cancer, the patient's overall health, and the overall health of the patient's liver.

Surgical Resection: This is often the first-line treatment for liver cancer if the disease is localized and the patient is healthy enough to undergo surgery. This involves surgically removing the tumor and a margin of healthy tissue around it.

Liver Transplantation: In patients with early-stage liver cancer who have significant underlying liver disease, a liver transplant can be considered. This involves replacing the diseased liver with a healthy liver from a donor.

Radiofrequency Ablation (RFA) or Microwave Ablation (MWA): These are minimally invasive procedures that use heat to destroy the cancer cells. They are often used for small tumors when surgery is not an option.

Cryoablation: This technique uses extreme cold to destroy the tumor.

Transarterial Radioembolization (TARE) or Selective Internal Radiation Therapy (SIRT): These are similar to TACE, but instead of chemotherapy, radioactive microspheres are injected into the arteries that feed the tumor.

Systemic Chemotherapy: This involves administering chemotherapy drugs that circulate throughout the body. This is often used for cancer that has spread beyond the liver or in cases where TACE or other local therapies are not an option.

Targeted Therapies or Immunotherapies: These are newer types of drugs that specifically target the cancer cells or boost the body's immune system to fight the cancer.

Percutaneous Ethanol Injection (PEI): This technique involves injecting alcohol directly into the tumor to kill cancer cells.

Each of these treatments has its own advantages and disadvantages, and the choice between them will depend on the specifics of the patient's case. In many cases, a combination of treatments will be used. It's also important to note that advances in medical research may lead to the development of new treatments over time. The decision on the most suitable treatment should be made in discussion with a multidisciplinary team of healthcare providers.

8. What are treatment goals of TACE?

The treatment goals of Transarterial Chemoembolization (TACE) can vary depending on the specifics of each patient's case, but generally include the following:

Tumor Shrinkage: By delivering a high dose of chemotherapy directly to the tumor and then blocking off its blood supply, TACE can often shrink tumors significantly. This can be particularly beneficial in cases where the patient might become eligible for curative treatments (like surgical resection or liver transplantation) if the tumor can be reduced in size.

Disease Control: For some patients, the goal might not necessarily be to shrink the tumor, but rather to prevent it from growing or spreading further. This can help to maintain the patient's quality of life and potentially extend their lifespan.

Symptom Management: In some cases, TACE can be used to help manage symptoms caused by liver tumors, such as pain or discomfort. By shrinking the tumor, TACE can alleviate these symptoms and improve the patient's quality of life.

Bridging or Downstaging to Transplant: In certain patients with hepatocellular carcinoma (HCC) awaiting liver transplantation, TACE is used as a bridge to transplant (to prevent tumor progression during the waiting time) or for downstaging (to reduce the tumor burden to meet transplantation criteria).

Combination Therapy: TACE can also be used in combination with other treatments, such as systemic therapies (chemotherapy, targeted therapies, immunotherapy) or radiation therapy, with the goal of enhancing the overall effectiveness of the treatment plan.

The specific goals of TACE are usually discussed with the patient by their medical team before the procedure, so that the patient has a clear understanding of what the treatment is expected to achieve.

9. Can TACE cure cancer?

Transarterial Chemoembolization (TACE) is generally not considered a curative treatment for liver cancer, but it can be a valuable part of the overall treatment strategy in certain cases.

TACE is mainly used as a palliative treatment, which means it's designed to reduce the size of the tumor, slow its growth, or relieve symptoms, rather than to completely eliminate the cancer. It's often used in cases where the cancer is not suitable for potentially curative treatments like surgical

resection or liver transplantation, either because the tumor is too large or the patient's overall health is not strong enough to withstand these procedures.

In some cases, TACE can be used to shrink tumors to a size where they can be surgically removed or treated with other therapies, or to bridge patients to liver transplantation. In these cases, TACE can contribute to a cure when used as part of a multimodal treatment approach.

However, while TACE can effectively treat the tumors it targets, it may not address cancer that has spread outside the liver or tiny metastases within the liver that aren't visible on imaging. Therefore, TACE is often used in combination with systemic therapies that can address cancer throughout the body.

As always, the goal of TACE and the potential for a cure depend on many individual factors including the type and stage of cancer, the patient's overall health, the health of the liver, and other factors. These should be discussed with the treating physician.

10. Can TACE be performed in addition to regular chemotherapy?

Yes, Transarterial Chemoembolization (TACE) can often be performed in addition to regular, systemic chemotherapy. This is known as combination therapy. The aim of this approach is to enhance the overall effectiveness of treatment by attacking the cancer from multiple angles.

TACE specifically targets the tumors in the liver, delivering a high dose of chemotherapy directly to the tumor and blocking off its blood supply. This can shrink the tumors and help to control the disease in the liver.

Systemic chemotherapy, on the other hand, involves administering chemotherapy drugs that circulate throughout the entire body. This can be beneficial for controlling cancer that has spread beyond the liver or to other parts of the body.

Using TACE in combination with systemic chemotherapy can sometimes lead to improved outcomes compared to using either treatment alone. However, it's important to note that this approach might not be suitable for everyone, and the potential benefits need to be weighed against the risk of increased side effects.

The decision to use TACE in combination with systemic chemotherapy should be made in consultation with a multidisciplinary team of medical professionals, who can help assess the potential risks and benefits in the context of the individual patient's case.

Preparation and procedure:

1. How should I prepare for TACE?

Your doctor or healthcare provider will give you specific instructions on how to prepare for your Transarterial Chemoembolization (TACE) procedure, but here are some general guidelines:

Medications: You may be asked to stop taking certain medications several days before the procedure, particularly medications that affect blood clotting, such as aspirin, warfarin, or other blood thinners. However, never stop taking any medication without first consulting with your healthcare provider.

Fasting: You'll typically be asked to not eat or drink anything for a few hours before the procedure, usually after midnight on the night before the procedure.

Pre-procedure Testing: Your healthcare provider may order certain tests before your procedure, such as blood tests or imaging studies. These tests help your doctor assess your overall health and plan the procedure.

Transportation: As you will likely be sedated for the procedure, plan to have someone drive you home afterwards.

Hospital Stay: Depending on the specifics of your case and how the procedure goes, you may need to stay in the hospital overnight for observation after TACE.

Allergies: Inform your doctor if you have any allergies, especially to iodine or any contrast materials which are often used in TACE procedures.

Remember to follow your doctor's specific instructions carefully and ask any questions you might have about what you need to do to prepare for the procedure. Always consult your healthcare provider if you're unsure about any aspect of the preparation process.

2. Will I need to fast before the procedure?

Yes, you will typically be asked to fast before a Transarterial Chemoembolization (TACE) procedure. This usually means not eating or drinking anything for a certain number of hours before the procedure. Often, you're instructed to stop eating and drinking after midnight on the night before the procedure.

The exact length of time you need to fast can depend on a number of factors, including the specifics of your procedure and your overall health, so it's important to follow the specific instructions given to you by your healthcare provider.

Fasting before a procedure like TACE is important because the procedure typically involves sedation or anesthesia, which can affect the reflexes that normally prevent you from aspirating food or liquid into your lungs.

If you have any questions about how to prepare for your procedure, be sure to ask your healthcare provider.

3. Should I continue taking my medications before TACE?

Whether or not you should continue taking your medications before undergoing Transarterial Chemoembolization (TACE) will depend on the specific medications you're taking and the potential impact they could have on the procedure.

Blood thinners: If you're on blood thinning medications (anticoagulants) like warfarin, clopidogrel, or daily aspirin, you might be asked to stop taking these for several days before the procedure, as they can increase the risk of bleeding. However, it's important that this is done under the guidance of your doctor, as stopping these medications may increase the risk of a clot or stroke in certain patients.

Diabetes medications: Patients with diabetes might need to adjust their medication regimen due to the fasting required before the procedure. Your doctor will provide specific instructions based on your situation.

Other medications: For most other medications, including blood pressure medications and drugs for other chronic conditions, you'll likely be advised to continue taking them as usual.

In all cases, it's critical that you discuss all of the medications you're currently taking with your healthcare provider well in advance of your TACE procedure. This includes prescription medications, over-the-counter drugs, herbal supplements, and vitamins. They will provide specific instructions on what you should and shouldn't take before the procedure.

Never stop taking any medication without first consulting with your healthcare provider.

4. Will I be awake during the procedure?

During a Transarterial Chemoembolization (TACE) procedure, you'll typically be given conscious sedation. This means that you'll be relaxed and drowsy, but not fully unconscious as you would be under general anesthesia. You might not remember much, if anything, about the procedure afterward.

Conscious sedation is typically used for TACE because it allows the patient to be comfortable and reduces anxiety during the procedure, but doesn't require the same level of monitoring as general anesthesia and typically has fewer risks and side effects.

The exact type of sedation or anesthesia you receive will depend on the specifics of your case, including your overall health and the policies and practices of the facility where you're having the procedure. You should discuss this with your doctor or anesthesiologist to make sure you understand what to expect during your TACE procedure.

5. How is the procedure performed?

Transarterial Chemoembolization (TACE) is a minimally invasive procedure performed by an interventional radiologist. Here's a basic overview of how the procedure is typically performed:

Preparation: You'll be positioned on the procedure table. Monitors are attached to your body to track vital signs like heart rate, blood pressure, and oxygen levels throughout the procedure. You'll then be given a sedative to help you relax and local anesthesia to numb the area where the catheter will be inserted, typically in the groin.

Accessing the Artery: The radiologist will make a small incision in the skin and insert a thin, flexible tube called a catheter into the femoral artery, a large artery in your thigh.

Guiding the Catheter: Using X-ray guidance, the radiologist will guide the catheter up through the artery and into the liver. Contrast dye will be injected through the catheter and X-ray images (fluoroscopy) will be taken to map out the blood vessels and to identify the ones feeding the tumor.

Administering the Treatment: Once the catheter is in place, the chemotherapy drug, mixed with lipiodol (an oily contrast agent), is delivered directly into the tumor. An embolizing agent is then injected, which blocks the blood flow to the tumor.

End of Procedure: After the treatment is administered, the catheter is carefully removed and the incision site is closed and bandaged.

Recovery: You'll be monitored in a recovery area for a few hours. Most patients stay in the hospital overnight for observation, although the length of the hospital stay can vary.

TACE can take several hours to complete. The specifics can vary based on the individual patient, the size and location of the tumor, and the exact techniques used by the radiologist.

6. How long will the procedure take?

The duration of a Transarterial Chemoembolization (TACE) procedure can vary widely based on a number of factors, including the specifics of the patient's case, the number and location of the tumors being treated, and the techniques used by the physician. On average, the procedure itself can take anywhere from one to three hours.

However, you'll need to factor in additional time for preparation before the procedure and recovery afterward. Before the procedure, you'll be prepped for the procedure and given sedation. Afterward, you'll be monitored in a recovery area while the sedation wears off, which can take a few hours. Most patients will also need to stay in the hospital overnight for observation, although the exact length of the hospital stay can depend on your individual circumstances and how you recover from the procedure.

Your healthcare provider can give you a more accurate estimate based on your specific situation.

7. Will I feel any pain during TACE?

Transarterial Chemoembolization (TACE) is performed under sedation and local anesthesia, so you should not feel pain during the actual procedure. The local anesthesia will numb the area where the catheter is inserted, typically in your groin, and the sedation will help you relax and may make you drowsy or even sleep.

However, it's possible to experience some discomfort during the procedure. For example, you may feel some pressure when the catheter is inserted and guided through the artery, although this should not be painful.

After the procedure, as the sedation and anesthesia wear off, you may feel some pain or discomfort in the area where the catheter was inserted. You may also experience post-embolization syndrome, a common side effect of TACE, which includes symptoms like fever, nausea, vomiting, loss of appetite, and abdominal pain. This is due to the death of tumor cells and generally subsides within a few days. Pain medication can be given to manage these symptoms.

It's important to remember that everyone's experience is different, and your healthcare provider will work to ensure your comfort throughout the procedure and during your recovery. If you have any concerns about pain or discomfort, don't hesitate to discuss them with your healthcare provider.

8. Will I be able to feel or sense anything during the TACE?

During the Transarterial Chemoembolization (TACE) procedure, you will be under conscious sedation, meaning you'll be relaxed and drowsy but not fully unconscious. As a result, you may not remember much, if anything, about the procedure.

The local anesthetic used should numb the area where the catheter is inserted, typically in the groin, so you should not feel any pain in that area during the procedure. Some patients may feel a sensation of pressure or movement as the catheter is guided through the artery, but this is usually not painful.

After the sedation and local anesthesia are administered, you should be comfortable and mostly unaware of the details of the procedure. Your healthcare team will monitor you throughout the procedure to ensure that you're comfortable and to manage any discomfort that might arise.

After the procedure, you might experience some side effects such as post-embolization syndrome, which includes symptoms like fever, nausea, vomiting, loss of appetite, and abdominal pain. This is due to the death of the tumor cells and generally subsides within a few days. Pain medication can be given to manage these symptoms.

It's important to remember that everyone's experience with TACE is different. If you have any concerns, be sure to discuss them with your healthcare provider.

9. What type of anesthesia or sedation can be used during TACE?

During a Transarterial Chemoembolization (TACE) procedure, a combination of local anesthesia and conscious sedation is typically used.

Local anesthesia is used to numb the area where the catheter is inserted, often the groin area. This is typically a local anesthetic medication such as lidocaine.

Conscious sedation, also called moderate sedation or procedural sedation, is a combination of medicines to help you relax and to block pain during the procedure. It doesn't make you fully unconscious, but it does make you sleepy and you might not remember the procedure afterward. Sedation medications can include drugs like benzodiazepines (for example, midazolam) for relaxation and opioids (for example, fentanyl) for pain relief.

While under conscious sedation, you'll be able to breathe on your own without the need for a breathing tube, and your heart rate, blood pressure, and oxygen levels will be monitored continuously.

The exact medications used can vary based on your individual health condition, your reaction to medications, the policy of the healthcare facility, and the preference of the physician. You should discuss this with your doctor or anesthesiologist to understand what will be used for your procedure.

10. How will I be monitored during the procedure?

During a Transarterial Chemoembolization (TACE) procedure, your vital signs will be monitored closely to ensure your safety and comfort. Here's how you'll typically be monitored:

Heart Rate and Rhythm: An electrocardiogram (ECG or EKG) will be used to monitor your heart rate and rhythm. Sticky electrode patches will be placed on your skin to transmit information about your heart's electrical activity to the monitor.

Blood Pressure: A blood pressure cuff will be placed on your arm and will automatically inflate at regular intervals to measure your blood pressure.

Oxygen Levels: A pulse oximeter, a small device that clips onto your finger or earlobe, will be used to monitor the level of oxygen in your blood. It works by shining a light through your skin and measuring the amount of light that's absorbed.

Breathing Rate: Your breathing rate will also be monitored, often simply by observing your chest movements.

Consciousness Level: Although you will be sedated, the medical team will monitor your level of consciousness and can adjust the sedation as necessary.

Body Temperature: In some cases, your body temperature might also be monitored.

The healthcare team will be there throughout the procedure to watch these monitors and ensure you're safe and comfortable. They can intervene if any problems arise. After the procedure, you'll continue to be monitored until the sedation wears off and you're stable.

Risks and Complications:

1. What are the possible risks and complications of TACE?

As with any medical procedure, Transarterial Chemoembolization (TACE) carries potential risks and complications. While TACE is generally safe and well-tolerated, it's important to be aware of the potential complications. These can include:

Post-Embolization Syndrome: This is the most common side effect of TACE and includes symptoms like fever, nausea, vomiting, loss of appetite, and abdominal pain. This is usually due to the death of the tumor cells and generally subsides within a few days. It can be managed with medications.

Bleeding or Hematoma: As with any procedure that involves the puncture of a blood vessel, there's a risk of bleeding or hematoma (a collection of blood) at the site where the catheter was inserted.

Infection: Although rare, there's a risk of infection at the site where the catheter was inserted or systemically.

Damage to the Liver: TACE can cause damage to the liver, especially in patients whose liver function is already compromised.

Non-target Embolization: There's a risk that the chemotherapy drug or the embolizing agent could affect healthy tissues, either in the liver or elsewhere in the body.

Allergic Reaction: There's a risk of allergic reaction to the contrast dye or the embolizing agent used in the procedure.

Gallbladder Inflammation (Cholecystitis): In rare cases, TACE can lead to inflammation of the gallbladder.

Kidney Damage: The contrast dye used in TACE can, in rare cases, cause kidney damage, particularly in patients with pre-existing kidney disease.

It's important to discuss these potential risks with your doctor so that you can make an informed decision about your treatment. Your doctor will also take steps to minimize these risks, such as prescribing antibiotics to prevent infection or administering medications to help manage post-embolization syndrome.

2. How common are these risks and complications?

The frequency of risks and complications from Transarterial Chemoembolization (TACE) can vary based on the specific patient, their overall health, the size and location of their tumor(s), and other factors. Here are general estimates for some of the most common complications:

Post-Embolization Syndrome: This is the most common side effect of TACE and can occur in up to 80% of patients. However, these symptoms are usually mild to moderate and can be managed with medications.

Bleeding or Hematoma: The risk of significant bleeding or hematoma at the puncture site is low, occurring in less than 5% of cases in general.

Infection: The risk of infection is quite low, estimated to be less than 1% for most patients.

Liver Damage: The risk of liver damage varies widely based on the patient's pre-existing liver function and the specifics of their treatment. For most patients, any liver damage is temporary and reversible.

Non-target Embolization: The risk of non-target embolization is low due to the careful targeting of the chemotherapy drug and embolizing agent. The exact rate can vary based on the techniques used.

Allergic Reaction: The risk of allergic reaction to the contrast dye or the embolizing agent is low, but can be higher in patients with a known allergy to these substances.

Gallbladder Inflammation (Cholecystitis): This is a rare complication, occurring in less than 5% of cases.

Kidney Damage: The risk of kidney damage is low for most patients but can be higher in patients with pre-existing kidney disease.

These are general estimates, and the actual risk can vary based on individual circumstances. Your doctor can provide more specific information based on your individual health condition and the specifics of your treatment plan.

3. Are there any specific factors that increase my risk of complications?

Yes, certain factors can increase the risk of complications from Transarterial Chemoembolization (TACE). These include:

Poor Liver Function: Patients with advanced liver disease or poor liver function are at a higher risk for complications. The liver plays a crucial role in metabolizing and clearing the chemotherapeutic agents used in TACE, so any existing liver dysfunction can make this process more difficult and potentially harmful.

Kidney Disease: Patients with kidney disease may be at a higher risk for complications due to the use of contrast dye in the procedure, which is processed by the kidneys.

Cardiovascular Disease: Patients with heart disease or other cardiovascular conditions may have an increased risk of complications from the sedation or anesthesia used during the procedure, as well as from the stress of the procedure on the cardiovascular system.

Bleeding Disorders or Anticoagulant Therapy: Patients with bleeding disorders or those taking blood-thinning medications are at an increased risk of bleeding complications.

Multiple Tumors or Large Tumors: Patients with multiple tumors or large tumors may be at a higher risk for complications due to the larger amount of chemotherapeutic agents and embolic material needed, which can increase the stress on the liver and other organs.

Previous Allergic Reactions: Patients who have had previous allergic reactions to contrast dye, the chemotherapeutic agents, or embolic materials used in TACE may have an increased risk of allergic reactions during the procedure.

Advanced Age or General Health: Older patients or those in poor general health may have a higher risk of complications simply because their bodies may have more difficulty tolerating the procedure and recovering afterward.

Your doctor will consider all these factors when deciding whether TACE is the right treatment for you and when planning your procedure to minimize the risk of complications.

4. What steps are taken to minimize the risks during the procedure?

Several steps are taken to minimize the risks and potential complications during a Transarterial Chemoembolization (TACE) procedure:

Patient Evaluation: Before the procedure, the healthcare team conducts a thorough evaluation of the patient's overall health, medical history, and current health status. This can include blood tests, imaging studies, and other diagnostic tests to assess liver function, kidney function, heart function, and the location and extent of the cancer. This helps ensure the patient is a good candidate for TACE and allows the team to plan the procedure carefully.

Preparation: Patients may be given medications to prevent nausea and pain, and in some cases, antibiotics to prevent infection. If a patient is known to have allergies to contrast dye, medications can be given beforehand to mitigate the allergic response.

Technique: The interventional radiologist uses imaging guidance to precisely target the chemotherapy drug and embolizing agent to the tumor, minimizing the exposure of healthy tissues.

Monitoring: During the procedure, the patient's vital signs (heart rate, blood pressure, oxygen levels, etc.) are monitored closely. This allows the team to respond quickly if any problems arise.

Aftercare: After the procedure, patients are monitored in a recovery room until the sedation wears off. They're typically observed overnight in the hospital. The healthcare team manages any pain or discomfort, and monitors for any signs of complications.

Follow-up: Patients have follow-up appointments to check their recovery progress, manage any side effects, and assess the effectiveness of the treatment. Additional imaging studies may be done to evaluate the response of the tumor to the treatment.

These steps help ensure that TACE is as safe and effective as possible. However, like all medical procedures, TACE does carry some risk, and it's important for patients to discuss these risks with their doctor.

5. Can my liver be damaged?

Yes, one potential risk of Transarterial Chemoembolization (TACE) is damage to the liver. However, this is usually a temporary and reversible condition, especially when the procedure is carefully planned and executed by a skilled interventional radiologist.

In TACE, the aim is to deliver high doses of chemotherapy drugs directly to the tumor while minimizing exposure to the rest of the liver and the body. However, even with this precision, there can be some degree of injury to the non-cancerous parts of the liver. This is because the embolization material also blocks the blood supply to some healthy liver tissue.

Patients with pre-existing liver disease or poor liver function are at a higher risk of liver damage from TACE. This is because their livers may be less able to tolerate the procedure and recover afterwards.

Liver damage after TACE can manifest in several ways, including changes in liver function tests, symptoms of liver failure (such as jaundice, ascites, and encephalopathy), and in severe cases, liver necrosis or abscess.

While liver damage is a concern, TACE has been shown to be safe and effective for many patients, even those with some degree of liver dysfunction. The benefits of controlling the cancer often outweigh the potential risks. Each patient's individual risk profile and treatment plan should be discussed thoroughly with their healthcare provider.

6. Will my liver function be impaired after the procedure?

Following Transarterial Chemoembolization (TACE), there can be a temporary impairment of liver function. This is largely due to the high dose of chemotherapy and the embolizing agents delivered directly to the liver. Some degree of liver injury can occur, as the procedure involves blocking blood vessels that not only feed the tumor but also some healthy liver tissue.

Immediately after the procedure, there can be changes in liver function tests, and some patients may experience symptoms such as fatigue or mild jaundice. These changes are usually temporary and most patients recover their liver function over time.

Patients with pre-existing liver disease or compromised liver function are at a higher risk for more significant liver function impairment. This is why TACE is typically recommended for patients with relatively preserved liver function.

To monitor the liver's health after TACE, doctors will typically order liver function tests. These blood tests measure levels of certain proteins and enzymes that indicate how well the liver is working.

It's important to have a thorough discussion with your doctor about the potential risks and benefits of TACE in your particular situation. This will help you make an informed decision about your treatment.

7. Can I die during the procedure?

While serious complications or death can occur with any medical procedure, it's important to note that the risk of death during or immediately following Transarterial Chemoembolization (TACE) is very low. TACE is generally considered to be a safe procedure, particularly when performed by a skilled and experienced interventional radiologist in a well-equipped facility.

The majority of complications that do occur are typically manageable and non-fatal, such as post-embolization syndrome (a group of symptoms including fever, abdominal pain, and nausea), local hematoma or bleeding at the catheter insertion site, or temporary changes in liver function.

Serious complications, such as liver failure, severe infection, or major bleeding, are rare. However, the risk can be increased in patients with significant underlying health issues, such as severe liver disease or heart disease.

Your healthcare team will take numerous precautions to minimize the risks, including thorough pre-procedure evaluation and preparation, careful procedural techniques, and comprehensive post-procedure care and monitoring.

Despite these assurances, it's natural to feel anxious about a procedure like TACE. I encourage you to discuss any concerns with your healthcare provider. They can provide detailed information about the procedure, the risks, and how those risks apply to your personal health status.

8. Are there any complications that can occur after the procedure?

Yes, complications can occur after Transarterial Chemoembolization (TACE), although they're generally rare. The most common complication is post-embolization syndrome, which includes symptoms like fever, nausea, vomiting, and abdominal pain. This is due to the death of the tumor cells and generally subsides within a few days. It can be managed with medications.

However, other potential complications can include:

Liver Damage: Although TACE targets the tumor, it can also affect some of the healthy liver tissue, potentially leading to temporary or permanent liver damage.

Infection: As with any invasive procedure, there is a risk of infection at the catheter insertion site or in the bloodstream. In rare cases, abscesses can form in the liver.

Bleeding: While rare, there can be bleeding at the site where the catheter was inserted, or less commonly, internally.

Gallbladder Inflammation (Cholecystitis): In rare cases, TACE can lead to inflammation of the gallbladder.

Non-target Embolization: Though the procedure is guided by imaging, there's a small chance that the chemotherapy drug or embolizing agent could affect healthy tissues, either in the liver or elsewhere in the body.

Allergic Reactions: Some people may have an allergic reaction to the contrast dye or the embolizing agent used in the procedure.

Kidney Damage: The contrast dye used in TACE can, in rare cases, cause kidney damage, particularly in patients with pre-existing kidney disease.

After the procedure, you will be closely monitored for any signs of these complications. If you experience any unexpected symptoms or feel unwell, it's important to seek medical attention promptly. In many cases, complications can be effectively managed if they are detected and treated early.

9. Are there any long-term risks or complications associated with TACE?

While Transarterial Chemoembolization (TACE) is generally safe, it can potentially lead to some long-term risks or complications, although these are relatively rare. Here are a few potential long-term risks:

Chronic Liver Damage: While TACE specifically targets the cancer, it can also cause some damage to healthy liver tissue. This can potentially lead to long-term liver damage, especially in patients who have pre-existing liver disease. However, it's important to note that careful patient selection and procedure planning can help minimize this risk.

Kidney Damage: The contrast material used in the procedure can, in rare cases, lead to kidney damage, especially in patients with pre-existing kidney disease. This is usually a more immediate complication, but in some cases, it could have long-term effects.

Bile Duct Injury: In rare instances, TACE can cause injury to the bile ducts within the liver, which can lead to chronic problems such as strictures (narrowing) or leaks.

Cancer Progression: While TACE is generally effective at controlling liver cancer, it does not cure the disease. Therefore, there is always a risk that the cancer may progress over time, either within the liver or elsewhere in the body.

Your healthcare provider will discuss all potential risks and complications with you before you decide to proceed with TACE. They will also closely monitor your health both immediately after the procedure and in the long term to detect and manage any potential complications as early as possible. It's important to maintain regular follow-up appointments after TACE, even if you're feeling well, to ensure that any potential problems are caught early.

10. Does the cytostatic medication have a systemic effect?

Transarterial Chemoembolization (TACE) is designed to deliver high concentrations of chemotherapy (cytostatic) drugs directly to the tumor in the liver while limiting systemic exposure. The chemotherapy is mixed with an embolic agent that helps block the arterial blood supply to the tumor, trapping the chemotherapy within the tumor and reducing its spread to the rest of the body.

However, despite these measures, a small amount of the chemotherapy drug may still escape into the systemic circulation. The amount is typically much less than what would be seen with systemic chemotherapy administration, and therefore the side effects are usually much less severe.

In general, the systemic effects of the chemotherapy used in TACE are minimal and often temporary. If patients do experience systemic side effects, they are typically mild and may include fatigue, mild nausea, or temporary changes in blood counts. It is important to discuss any side effects or concerns with your healthcare provider, who can provide guidance and potential solutions.

11. Will I lose my hair?

Hair loss is a common side effect of systemic chemotherapy, which circulates throughout the body and affects rapidly dividing cells, including those in hair follicles. However, in Transarterial Chemoembolization (TACE), chemotherapy is delivered directly into the blood vessels supplying the tumor, which reduces the amount of drug exposure to the rest of the body.

Because the chemotherapy is targeted to the tumor and the amount of drug that enters the systemic circulation is relatively small, hair loss is not a common side effect of TACE. The systemic side effects are typically much less severe than those seen with systemic chemotherapy.

That being said, every patient responds differently, and side effects can vary. If you experience any unexpected side effects, it's important to discuss them with your healthcare provider. They can provide guidance and potential solutions to manage these issues.

Postinterventional aftercare:

1. What should I consider after TACE?

After undergoing Transarterial Chemoembolization (TACE), there are several things you should keep in mind:

Follow-up Appointments: Regular follow-up appointments with your healthcare provider are essential. These appointments allow your healthcare provider to monitor your recovery, manage any side effects or complications, and assess the effectiveness of the treatment.

Monitoring Side Effects: You may experience side effects following TACE, such as post-embolization syndrome (characterized by fever, pain, nausea, and vomiting), fatigue, and loss of appetite. These are typically temporary, but if they persist or worsen, contact your healthcare provider.

Pain Management: You might experience some pain or discomfort after the procedure. Your healthcare provider will likely prescribe pain medication to help manage this.

Activity Levels: You may feel tired after the procedure. It's important to rest and recover, but also to gradually increase your activity levels as tolerated.

Wound Care: You will need to care for the wound at the catheter insertion site according to your healthcare provider's instructions to prevent infection.

Medications: You may be prescribed medications to manage side effects, and it's important to continue taking your regular medications unless advised otherwise by your doctor.

Healthy Lifestyle: Maintaining a healthy lifestyle can help your recovery. This includes eating a balanced diet, staying hydrated, getting regular exercise as tolerated, and avoiding alcohol and tobacco.

Emotional Health: Dealing with cancer can be emotionally challenging. Reach out to supportive friends, family members, or professional counselors to help manage stress and anxiety.

Always consult your healthcare provider for advice tailored to your specific circumstances. They can provide the most accurate information based on your personal health status and treatment plan.

2. Will I experience pain after TACE?

Pain is a common side effect after Transarterial Chemoembolization (TACE). This is often part of a condition known as post-embolization syndrome, which includes symptoms such as abdominal pain, nausea, vomiting, and fever. The pain typically results from the inflammation and necrosis (cell death) of the tumor tissue due to the embolization and chemotherapy.

The level of pain varies between individuals. Some people may experience mild discomfort, while others may have more significant pain. This discomfort is usually most pronounced in the first few days after the procedure but typically improves over time.

Your healthcare team will provide you with pain management strategies, which may include prescription medications or over-the-counter pain relievers. It's important to use these as directed and to communicate with your healthcare provider if the pain is not well controlled or if it worsens over time. They can adjust your pain management plan as necessary to ensure your comfort.

It's also worth noting that pain after TACE is typically localized to the abdomen or right upper quadrant, where the liver is located. If you experience pain in other areas, or if the pain is associated with other concerning symptoms (such as chest pain or shortness of breath), you should seek medical attention promptly.

3. What pain medication can I take after the TACE?

After a Transarterial Chemoembolization (TACE) procedure, your healthcare provider will likely prescribe you a pain medication to help manage any discomfort you may be feeling. This can range from over-the-counter analgesics, such as acetaminophen (Tylenol), to prescription strength medications like opioids, depending on the intensity of your pain.

Nonsteroidal anti-inflammatory drugs (NSAIDs), such as ibuprofen, are often used for mild to moderate pain, but they should be used cautiously or avoided in some patients, particularly those with impaired kidney function or gastrointestinal problems, due to potential side effects.

If the pain is severe, your doctor may prescribe a stronger opioid painkiller such as morphine or oxycodone. These drugs are effective for severe pain, but they can have side effects like drowsiness, constipation, and risk of dependence if used for extended periods.

In some cases, medications to manage other symptoms of post-embolization syndrome, such as nausea and fever, might also be prescribed.

It's important to take your medications exactly as directed by your healthcare provider. If you're still experiencing significant pain despite taking the medication, or if you have any concerns about the medication, you should reach out to your healthcare provider. They can adjust your pain management plan as necessary to ensure your comfort.

Please note that while I strive to provide accurate information, you should always consult with your healthcare provider for personalized advice.

4. What kind of care should I provide for the puncture site or incision?

After Transarterial Chemoembolization (TACE), care for the puncture site or incision is an important part of your post-procedure recovery. Here are some general guidelines:

Keep the Area Clean and Dry: For the first day or two, it's usually recommended to keep the area clean and dry. You can usually shower after 24-48 hours, but it's best to avoid baths, swimming, or soaking the area until it has fully healed.

Dressing Changes: If there is a dressing over the puncture site or incision, you will be given instructions on how and when to change it. This typically involves gently removing the old dressing, cleaning the area with mild soap and water, and applying a new sterile dressing.

Observe for Infection: Monitor the puncture site for signs of infection, which may include increased redness, swelling, warmth, pain, or discharge (such as pus). Also, watch for systemic signs of infection, such as fever or chills.

Avoid Strain: Avoid strenuous activities or heavy lifting for a few days to a week after the procedure to prevent strain or injury to the site.

Pain Management: There may be some tenderness or discomfort at the puncture site. Over-the-counter pain relievers can usually manage this, but if the pain is severe or worsens over time, contact your healthcare provider.

Follow-Up Care: Attend all follow-up appointments with your healthcare provider, as they can check the puncture site or incision and monitor your overall recovery.

These are general guidelines and your healthcare provider may give you specific instructions based on your individual circumstances and overall health condition. Always follow the instructions given to you by your healthcare provider.

5. Are there any specific restrictions or limitations on activities following TACE?

After Transarterial Chemoembolization (TACE), you'll need to take it easy for a few days to allow your body to recover. While exact guidelines can vary based on individual health status and your healthcare provider's recommendations, here are some general post-procedure considerations:

Rest and Recovery: You may feel tired and less energetic following the procedure due to the effects of sedation, the procedure itself, and post-embolization syndrome. It's important to rest and allow your body to recover.

Physical Activity: Strenuous activities and heavy lifting should be avoided for a period of time (often about one week) following the procedure. Light activities, such as walking, can often be resumed quite quickly, but you should listen to your body and avoid pushing yourself too hard.

Driving and Operating Machinery: If you received sedation during the procedure, you should avoid driving or operating heavy machinery for at least 24 hours after the procedure.

Alcohol and Tobacco: Avoid alcohol and tobacco, as both can hinder the healing process and negatively impact your overall health.

Diet: You should be able to resume your normal diet after TACE, but some patients may initially experience nausea or lack of appetite. Try to eat balanced, nutritious meals to support your recovery.

Remember that these are general recommendations and your healthcare provider may give specific instructions based on your individual health status. Always follow their guidance for post-procedure care.

6. How long do I need to stay lying down after the procedure?

After Transarterial Chemoembolization (TACE), you'll likely need to lie flat and rest for several hours. This helps to minimize the risk of bleeding from the catheter insertion site, usually in the groin, where the blood vessels were accessed to perform the procedure.

The exact length of time can vary, but it's typically in the range of 4-6 hours. During this time, it's important to keep the leg that was used for the procedure straight. Your healthcare team will monitor you during this period and ensure that the puncture site is healing properly.

After this initial rest period, you'll gradually be able to get up and move around, initially with assistance, until you're able to move independently. This is usually by the next day, although it can vary depending on your individual health status and how quickly you recover from the procedure.

Your healthcare team will provide specific instructions based on your individual situation, and it's important to follow these instructions closely. If you have any questions or concerns, be sure to discuss them with your healthcare provider.

7. Will I be admitted as an inpatient?

Whether you will be admitted as an inpatient following Transarterial Chemoembolization (TACE) largely depends on your individual health status and the specific practices of your healthcare institution.

In many cases, TACE is performed as an outpatient procedure, which means you would come in for the procedure and go home the same day once you've recovered from the anesthesia and your medical team has determined that it's safe for you to leave.

However, in some cases, particularly if you have other significant health concerns or if complications arise during the procedure, you may need to be admitted as an inpatient for further observation or treatment. This could involve staying in the hospital overnight or for several days.

The decision about outpatient versus inpatient treatment will be made by your healthcare team based on a variety of factors, including the specifics of your case, your overall health, the success of the procedure, and your recovery immediately after the procedure.

Always consult with your healthcare provider to understand the specifics of your care plan.

8. What signs or symptoms should I watch for that may indicate a problem or complication?

After Transarterial Chemoembolization (TACE), it's normal to experience some discomfort and side effects, but certain signs and symptoms could indicate a complication. Contact your healthcare provider immediately if you notice any of the following:

Severe Pain: Some pain is expected after the procedure, but severe or increasing pain may indicate a problem.

Fever and Chills: These could be signs of infection.

Signs of Infection at the Catheter Insertion Site: Increased redness, swelling, warmth, or drainage (such as pus) from the site where the catheter was inserted can be signs of an infection.

Increased Bleeding or Bruising at the Catheter Site: Some bruising is normal, but if you notice increasing bleeding, large or expanding bruises, or if the site becomes very swollen or painful, these could be signs of a hematoma or a bleed.

Change in Color, Temperature, or Sensation in the Leg Used for the Procedure: These could indicate a problem with blood flow to the leg.

Chest Pain or Shortness of Breath: These could potentially be signs of a heart problem or a pulmonary embolism, which is a serious but rare complication.

Severe Nausea or Vomiting: While mild nausea can be a side effect of the procedure or the anesthesia, severe or persistent nausea or vomiting may indicate a complication.

Severe Fatigue or Confusion: These could be signs of a serious complication like liver failure or a systemic infection.

Jaundice: Yellowing of the skin or eyes could indicate liver damage or other liver problems.

These signs don't necessarily mean there is a serious complication, but they should be evaluated by a healthcare provider promptly. It's always better to err on the side of caution when it comes to potential complications.

9. For how long will I typically need to stay in hospital after TACE?

The length of hospital stay after Transarterial Chemoembolization (TACE) varies depending on the individual's overall health status, the complexity of the procedure, the institution's protocol, and whether any complications occur.

TACE can be performed as an outpatient procedure, which means you might be able to go home on the same day of the procedure, typically after a period of observation to ensure there are no immediate complications. However, some patients may be kept overnight for monitoring, especially if they have other significant health concerns, experience severe post-procedure symptoms, or if any complications arise during the procedure.

In some cases, a longer stay of a few days may be required. This is usually if there are complications, or if the patient has other serious health issues that need monitoring or treatment.

Your healthcare provider will be able to give you the most accurate estimate based on your personal medical history and the specifics of your procedure. It's important to follow all discharge instructions and attend all follow-up appointments to ensure the best possible outcome.

10. Will I need any follow-up appointments?

Yes, you will need follow-up appointments after a Transarterial Chemoembolization (TACE) procedure. These appointments allow your healthcare provider to monitor your recovery, manage any side effects or complications, and assess the effectiveness of the treatment.

Here's a general idea of what to expect, although the specifics may vary based on your individual situation and your healthcare provider's protocols:

Short-term follow-up: A follow-up appointment is typically scheduled a few weeks after the procedure. This appointment is to check on your recovery, manage any side effects, and monitor your overall health. This may involve blood tests or imaging studies.

Long-term follow-up: Regular follow-up appointments will be scheduled for months to years after the procedure. The frequency of these appointments will depend on various factors, including the type and stage of your cancer, your overall health, and your response to the treatment. These appointments will typically involve imaging tests like CT or MRI to assess the size of the tumor and determine the effectiveness of the treatment.

Treatment adjustments: If the tumor is not responding to treatment, or if new tumors appear, the treatment plan may need to be adjusted. This could potentially involve additional TACE procedures, or other treatments may be considered.

It's very important to attend all scheduled follow-up appointments and to communicate openly with your healthcare provider. If you notice any new or worsening symptoms between appointments, you should contact your healthcare provider right away.

11. How often does TACE need to be repeated?

The frequency with which Transarterial Chemoembolization (TACE) needs to be repeated depends on several factors, including:

The Type and Stage of the Cancer: Some types of cancer respond better to TACE than others. Additionally, more advanced cancers may require more frequent treatments.

The Response to Treatment: If the tumor shrinks significantly after the first TACE procedure, fewer treatments may be necessary. However, if the tumor doesn't respond as well, more treatments might be needed.

The Patient's Overall Health: The patient's overall health and the function of their liver and other organs can also influence how often TACE can be safely repeated.

TACE can sometimes be performed in multiple sessions, with each session treating a different part of the liver. The sessions are usually spaced out over several weeks or months to allow the body time to recover.

In general, the decision to repeat TACE is made based on the patient's response to the treatment, the progression of the disease, and the patient's overall health status. Your healthcare provider will monitor your condition closely and make recommendations based on your specific situation.

Remember to consult with your healthcare provider for personalized advice.
